# Enhanced Anticancer Potential of Pd(II)-Thiosemicarbazone Complexes: Selectivity, Mechanisms, and 3D Models

**DOI:** 10.3390/pharmaceutics17070829

**Published:** 2025-06-25

**Authors:** Mauro A. Lima, Tamara Teixeira, Dario B. Fortaleza, George B. S. Pereira, Amos O. Akinyemi, Carlos André Ferreira Moraes, Moacir R. Forim, Alzir A. Batista, Jocely L. Dutra, João H. Araujo-Neto, Javier A. Ellena, Fillipe V. Rocha

**Affiliations:** 1 Departamento de Química, Universidade Federal de São Carlos, São Carlos 13565-905, São Paulo, Brazil; lima.mauroal@gmail.com (M.A.L.); tamara.teixeira.296@gmail.com (T.T.);; 2 Department of Toxicology and Cancer Biology, College of Medicine, University of Kentucky, Lexington, KY 40536, USA; 3 Departamento de Química Fundamental, Instituto de Química, Universidade de São Paulo, São Paulo 05508-000, São Paulo, Brazil; 4 Instituto de Física de São Carlos, Universidade de São Paulo, São Carlos 13566-590, São Paulo, Brazil

**Keywords:** Pd(II) complexes, thiosemicarbazones, cytotoxicity, ovarian cancer

## Abstract

**Background/Objectives:** Cancer remains a major global health challenge, driving the search for novel chemotherapeutic agents. This study aimed to evaluate the structural and biological properties of a series of **Pd(II)** complexes containing triphenylphosphine and thiosemicarbazone ligands, in order to assess their potential as anticancer agents. **Methods:** Six **Pd(II)** complexes with the general formula **[PdCl(PPh_3_)(TSC)]** were synthesized and fully characterized by NMR (^1^H, ^1^³C, ³^1^P), FTIR, mass spectrometry, and X-ray diffraction. Their cytotoxic effects were investigated through in vitro assays using 2D and 3D cancer cell models, including clonogenic, wound healing, cell cycle, and apoptosis assays via flow cytometry. **Results:** Complexes from the **B** family demonstrated significantly higher cytotoxicity than those from the C family, particularly against ovarian (*IC*_50_ < 1 µM) and breast (*IC*_50_~2 µM) cancer cell lines. These compounds exhibited superior potency and selectivity compared to cisplatin, with high selectivity indices toward non-tumor cells. Mechanistic studies revealed both cytotoxic and cytostatic effects depending on structural variations, with apoptosis identified as the primary mechanism of cell death. **PdB1**, in particular, induced a marked increase in late apoptotic populations and maintained its cytotoxic activity in 3D spheroid models by promoting disintegration, loss of cell adhesion, and nuclear fragmentation. **Conclusions:** The findings underscore the therapeutic promise of **Pd(II)** complexes, especially **PdB1**, as potent and selective antineoplastic agents capable of acting effectively in complex tumor environments and potentially overcoming chemoresistance.

## 1. Introduction

Cancer is characterized by the uncontrolled proliferation of cells caused by genetic and epigenetic alterations that disrupt normal regulatory mechanisms, including those that control apoptosis. These transformed cells can form primary tumors and, in malignant forms, invade surrounding tissues and metastasize to distant organs, driving disease progression and complicating treatment outcomes [1,2,3]. This complexity underscores the ongoing need for innovative therapeutic strategies to improve clinical outcomes. Chemotherapy remains a central component of treatment protocols, using drugs that interfere with cell division or induce programmed cell death [4,5,6].

Cisplatin, a platinum-based chemotherapeutic agent, is widely used in the treatment of a variety of solid tumors. Its cytotoxicity is due to the aquation of its chloride ligands, which enables covalent binding to DNA and the formation of intra- and interstrand crosslinks [7,8,9,10]. These adducts inhibit DNA replication and transcription, ultimately inducing apoptosis. Despite its efficacy, the long-term success of cisplatin is limited by several factors. In particular, the emergence of drug resistance, whether intrinsic or acquired, significantly reduces its therapeutic potential. Resistance mechanisms include increased drug efflux, enhanced DNA repair, inactivation by thiol containing biomolecules, and impaired apoptosis signaling [11]. In addition, severe side effects such as nephrotoxicity, neurotoxicity, and ototoxicity necessitate the development of alternative metal-based agents with improved safety and efficacy profiles.

In response to these limitations, researchers have explored a variety of metal ions as the basis for novel antitumor agents [12,13,14]. Palladium(II) stands out because of its chemical similarity to platinum(II), particularly in terms of coordination geometry and ligand exchange properties [15,16]. In contrast to cisplatin, **Pd(II)** complexes often exhibit faster ligand exchange kinetics, which can be modulated by appropriate ligand design. A notable example of a palladium-based drug is Tookad (padeliporfin), a Pd(II)-porphyrin complex approved for the treatment of prostate cancer via photodynamic therapy [17,18].

Thiosemicarbazones (**TSCs**) are versatile N,S-donor ligands derived from the condensation of thiosemicarbazides with aldehydes or ketones. Their ability to chelate metal ions combined with their intrinsic biological activity has led to extensive investigation of their antitumor, antiviral and antibacterial properties [19,20]. Among them, triapine is a clinically relevant **TSC** that is currently undergoing phase II and III trials for several types of cancer [21]. Structural modification of **TSCs**, particularly by substitution on the aromatic or aliphatic moieties, offers a powerful strategy to fine-tune pharmacological properties and metal-binding characteristics.

**Scheme 1:**
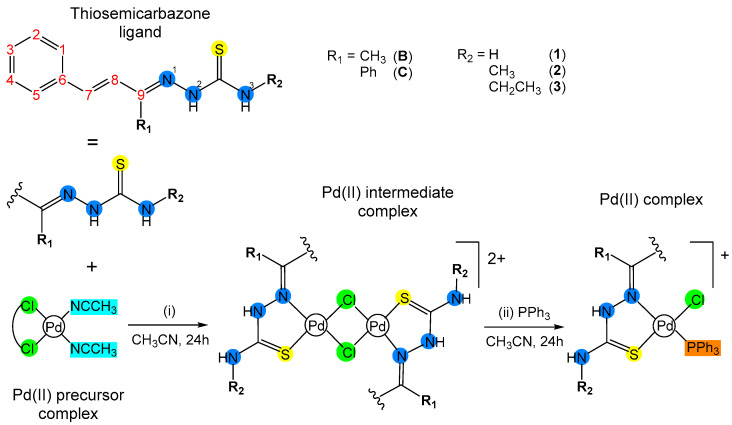
Reaction scheme for the formation of **Pd(II)** complexes.

In this context, the present study investigates the influence of structural variations in thiosemicarbazone ligands on the antitumor activity of **Pd(II)** complexes. Six **[PdCl(PPh_3_)(TSC)]** compounds were synthesized and fully characterized by NMR spectroscopy (^1^H, ^13^C, ^31^P), FTIR, high resolution mass spectrometry, and single crystal X-ray diffraction. The **TSC** ligands used in this work are derived from the ketones benzylideneacetone (**B**) and chalcone (**C**). The substituents on nitrogen 3 of the thiocarbazides are –H (**1**), –CH_3_ (**2**), and –CH_2_CH_3_ (**3**). The combination of these reagents results in the formation of ligands **B1**, **B2**, **B3**, **C1**, **C2**, and **C3**, and the complexation and addition of triphenylphosphine led to the formation of the complexes **PdB1**, **PdB2**, **PdB3**, **PdC1**, **PdC2**, and **PdC3**; see Scheme 1. It is important to mention that the PdB2 complex was reported in a previous work [22], and will have its biological studies extended in this work. Their cytotoxic potential was evaluated in both tumor and non-tumor cell lines, with further mechanistic studies addressing their ability to inhibit tumor cell proliferation, migration, and survival. Furthermore, 3D tumor models were investigated to gain deeper insight into their biological behavior and potential as next-generation antineoplastic agents [23].

## 2. Materials and Methods

### 2.1. Materials

The **PdB2** complex was synthesized previously by the author of this work, and its characterization is available in the literature [22]. The ligands used were provided by collaborators and the syntheses and characterizations are already reported [24].

The solvents used were from Labsynth, AcrosOrganics and Merck P.A. (Geel, Belgium). The reagents chloride palladium (II) (99%), triphenylphosphine (PPh_3_), Cesium iodide (99.99%), human serum albumin (HSA), calf thymus—DNA, DPPH, and MTT were purchased from Sigma-Aldrich (St. Louis, MO, USA).

### 2.2. Physical Measurements

^1^H, ^13^C{^1^H} and ^31^P{^1^H} NMR spectra were obtained using the BRUKER ARX 9.4T spectrometer (BRUKER, Billerica, MA, USA). Dept135, COSY, HSQC, and HMBC experiments were performed for the complex **PdC3**. All the data obtained was processed using the MestReNova 12.0 software.

The spectra in the infrared region were obtained on a SHIMADZU IRTracer-100 spectrophotometer (SHIMADZU, Kyoto, Japan), using CsI pellets as a support, and the data obtained was processed using Shimadzu IR Solution, 1.60 and Origin 9.0 software.

UHPLC-QTof-MS/MS was performed by direct infusion, using water (solvent A, 0.1% formic acid) and CH_3_CN (solvent C with 0.1% formic acid) as the mobile phase, in isocratic mode, in a ratio of 05:95 *v*/*v*, respectively. The mobile phase flow rate was 0.300 mL·min^−1^ and the injection volume was 3 µL. Quantitative analysis of the samples was performed using an Agilent 6545 qTOF MS system (Agilent Technologies, Santa Clara, CA, USA), equipped with a Jet electrospray interface (ESI) in positive mode. The error (in ppm) was calculated from the theoretical mass and the respective MS spectrum acquired. The calculation was carried out for the most intense isotope of the molecular ion [M]^+^. A range between 100 and 1000 Da was monitored, with a scan rate of 3 spectra s^−1^ and processed using Mass Hunter Workstation Software version B.08.00.

X-ray diffraction was obtained on a Rigaku XtaLAB mini II diffractometer, MoKα radiation (λ = 0.71073 Å). The SHELXT (2019) software was used to solve the structure using direct methods and successive Fourier difference maps, which allowed the non-hydrogen atoms to be located. Structural refinement was performed using the same software, using the least squares method. The unit cell parameters and absorption corrections were calculated using the CrysAlisPro171 software. Graphical representations of the molecules and crystallographic data tables were generated using the OLEX2 1.5 and MERCURY 4.2.0 software [25,26].

### 2.3. Synthesis of the Complexes

The palladium precursor complex, **[PdCl_2_(CH_3_CN)_2_]**, was obtained by slowly adding 500 mg of chloride palladium (II) to 50 mL of previously heated acetonitrile. After 4 h of reaction, the solution was concentrated by evaporating the solvent and the solid obtained was filtered. Yield = 80%.

To obtain the palladium complexes, 0.19 mmol of the precursor complex was solubilized in acetonitrile. Then, thiosemicarbazone ligand was added in a 1:1 ratio. After 24 h of reaction, at ambient temperature, 0.19 mmol of triphenylphosphine (**PPh_3_**) was added and the reaction medium was kept under agitation for a further 24 h. After this period, the volume of the solution was reduced and the solid obtained was filtered under vacuum.

[PdCl(**L-B1**)(PPh_3_)]Cl (**PdB1**) − Yield = 90%. C_29_H_28_Cl_2_N_3_PPdS. FTIR (cm^−1^): 3451-3237 νNH; 3050 νCH_sp_^2^; 1617 νC=CArH; 1542 δN-H; 1097 τ(P-CPh); 748 νC=S; 688 δC-HAr. ^1^H NMR (CDCl_3_, 400 MHz) δ(ppm): 13.51 (s, 1H, N(2)-H), 10.03 (s, 1H, N(3)-H), 8.55 (d, *J* = 16.1 Hz, 1H, H-8), 7.76 (m, 6H, o-HAr PPh_3_), 7.70 (m, 2H, H-1 + H-5), 7.57 (m, 3H, p-HAr PPh_3_), 7.48 (m, 6H, m-HAr PPh_3_), 7.37 (m, 3H, H-2 + H-3 + H-4), 7.24 (m, 1H, H-7), 6.10 (s, 1H, N(3)-H), 2.67 (s, 3H, R1(CH_3_)). ^31^P{^1^H} NMR (CDCl_3_, 162 MHz) δ(ppm) = 30.85. HRMS (QTof, positive mode) *m*/*z*: calculed: 622.0459; found: 622.0445 [M]^+^.

[PdCl(**L-B3**)(PPh_3_)]Cl (**PdB3**) − Yield = 85%. C_31_H_32_Cl_2_N_3_PPdS. FTIR (cm^−1^): 3408-3237 νNH; 3068-2916 νCH_sp_^2^; 1555 νC=CArH; 1514 δN-H; 1095 τ(P-CPh); 748 νC=S; 689 δC-HAr. ^1^H NMR (CDCl_3_, 400 MHz) δ(ppm): 12.92 (s, 1H, N(2)-H), 10.08 (s, 1H, N(3)-H), 8.50 (d, *J* = 16,1 Hz, 1H, H-8), 7.78 (m, 6H, o-HAr PPh3), 7.67 (m, 2H, H-1 + H-5), 7.57 (m, 3H, p-HAr PPh3), 7.49 (m, 6H, m-HAr PPh3), 7.36 (m, 3H, H-2 + H-3 + H-4), 7.18 (d, *J* = 16.1 Hz, 1H, H-7), 3.24 (q, dd *J* = 7.2; *J* = 5.0 Hz, H2, R2(CH_2_), 2.64 (s, 3H, R1(CH_3_), 1.22 (t, *J* = 7.1 Hz, 3H, R2(CH_3_)). ^31^P{^1^H} NMR (CDCl_3_, 162 MHz) δ(ppm) = 30.89. HRMS (QTof, positive mode) *m*/*z*: calculed: 650.0772; found: 650.0777 [M]^+^.

[PdCl(**L-C1**)(PPh_3_)]Cl (**PdC1**) − Yield = 65%. C_34_H_30_Cl_2_N_3_PPdS. FTIR (cm^−1^): 3379-3255 νNH; 3094-3002 νCH_sp_^2^; 1598 νC=CArH; 1533 δN-H; 1095 τ(P-CPh); 750 νC=S; 691 δC-HAr. ^1^H NMR (CDCl_3_, 400 MHz) δ(ppm): 8.07 (d, *J* = 16.1 Hz, 1H, H-8), 7.84 (m, 3H, R1(Ph)), 7.59 (m, 9H, H-1 + H-3 + H-5 + o-HAr PPh_3_), 7.52 (m, 2H, R1(Ph)), 7.46 (m, 3H, p-HAr PPh_3_), 7.38 (m, 6H, m-HAr PPh_3_), 7.36 (m, 2H, H-2 +H-4), 6.75 (d, *J* = 16.1 Hz, 1H, H-7). ^31^P{^1^H} NMR (CDCl_3_, 162 MHz) δ(ppm) = 29.83. HRMS (QTof, positive mode) *m*/*z*: calculed: 684.0616; found: 684.0612 [M]^+^.

[PdCl(**L-C2**)(PPh_3_)]Cl (**PdC2**) − Yield = 68%. C_35_H_32_Cl_2_N_3_PPdS. FTIR (cm^−1^): 3460-3250 νNH; 3068-2907 νCH_sp_^2^; 1581 νC=CArH; 1489 δN-H; 1095 τ(P-CPh); 745 νC=S; 692 δC-HAr. ^1^H NMR de (CDCl_3_, 400 MHz) δ(ppm): 13.67 (s, 1H, N(2)-H), 10.18 (s, 1H, N(3)-H), 8.15 (d, *J* = 16.1 Hz, 1H, H-8), 7.84 (m, 2H, R1(Ph)), 7.77 (m, 2H, R1(Ph)), 7.64 (m, 3H, H-1 + H-5 + R1(Ph)), 7.57 (m, 6H, o-HAr PPh3), 7.51 (m, 3H, p-HAr PPh_3_), 7.42 (m, 9H, H-2 + H-3 + H-4 + m-HAr PPh_3_), 6.91 (d, *J* = 16.1 Hz, 1H, H-7), 2.91 (s, 3H, R2(CH_3_)). ^31^P{^1^H} NMR (CDCl_3_, 162 MHz) δ(ppm) = 31.70. HRMS (QTof, positive mode) *m*/*z*: calculed: 698.0772; found: 698.0773 [M]^+^.

[PdCl(**L-C3**)(PPh_3_)]Cl (**PdC3**) − Yield = 69%. C_36_H_34_Cl_2_N_3_PPdS. FTIR (cm^−1^): 3434-3236 νNH; 3054-2929 νCH_sp_^2^; 1587 νC=CArH; 1520 δN-H; 1097 τ(P-CPh); 745 νC=S; 685 δC-HAr. ^1^H NMR (CDCl_3_, 400 MHz) δ(ppm): 13.67 (s, 1H, N(2)-H), 10.27 (s, 1H, N(3)-H), 8.18 (d, *J* = 16.1 Hz, 1H, H-8), 7.86 (m, 2H, R1(Ph_3_)), 7.79 (m, 2H, R1(Ph_3_)), 7.62 (m, 3H, H-1 + H-5 + R1(Ph_3_)), 7.58 (m, 6H, o-HAr PPh_3_), 7.51 (m, 3H, p-HAr PPh_3_), 7.42 (m, 9H, H-2 + H-3 + H-4 + m-HAr PPh_3_), 6.90 (d, *J*=16.1 Hz, 1H, H-7) 3.29 (dd *J* = 7.2; *J* = 5.0 Hz, 2H, R2(-CH_2_)), 1.23 (t, *J* = 7.2 Hz, 3H, R2(-CH_3_)). ^31^P{^1^H} NMR (CDCl_3_, 162 MHz) δ(ppm) = 31.71. HRMS (QTof, positive mode) *m*/*z*: calculed: 712.0929; found: 712.0931 [M]^+^.

### 2.4. Cell Culture Tests (In Vitro)

The cell lines used in the tests were A2780 (ovarian cancer), A2780cis (ovarian cisplatin resistant cancer), A549 (lung cancer), MRC5 (non-tumoral lung cancer), MDA-MB-231 (triple-negative breast cancer), MCF-7 (breast cancer), SK-BR-3 (breast cancer), MCF-10A (non-tumoral breast cancer), and A375 (skin cancer—melanoma).

The MCF-10A cell line was grown in DMEM/F-12 culture medium, supplemented with 5% horse serum, human epidermal growth factor (EGF) (20 ng/mL), hydrocortisone (0.05 mg/mL), insulin (0.01 mg/mL), penicillin (1%), streptomycin (1%), and L-glutamine (2 mM). The A2780, A2780cis, and MCF-7 cell lines were cultured in RPMI-1640 medium, and the other lines in DMEM medium; both culture media were supplemented with 10% (*v*/*v*) FBS (fetal bovine serum), antibiotics, and antimycotics. Cultivation was carried out in culture flasks stored in a humid incubator at 37 °C, with an atmosphere of 5% CO_2_.

### 2.5. Cell Viability (IC_50_)

The cell viability test to obtain the minimum inhibitory concentration of 50% (IC_50_) was made using a 96-well microplate, in which a 150 μL solution containing 1.5 × 10^4^ cells suspended in culture medium was added to each well. The plates were kept for 24 h in a humidified incubator (37 °C, 5% CO_2_), after which 0.75 μL of a DMSO solution (0.5% *v*/*v*) containing the compounds of interest was added to each well. The plates were then incubated for a further 48 h. The ratio of viability of cells was obtained using the MTT method. Through its reduction via the mitochondrial process, purple formazan is formed, which is proportional to cell viability. After incubating the microplates, 50 μL of a solution of MTT (1 mg∙mL^−1^) in PBS was added to each well and the cells were incubated again in the oven for 4 h. Finally, the solution was removed from each well and 100 μL of isopropyl alcohol with 10% DMSO was added to solubilize the Formazan crystals. Absorbance measurements (540 nm) were then taken in each well using a BioTek EPOCH spectrophotometer (BioTek, Winooski, WT, USA). The data obtained was analyzed using Excel 360 and GraphPadPrism 8.0.2.

### 2.6. Clonogenic Assay

For this test, 600 cells of the A2780cis strain were added to each well of a six-well microplate. After incubating for 24 h in a 5% CO_2_ atmosphere, different concentrations of the compounds were added to the wells and incubated again for five days. After this period, the culture medium was changed and the plates were incubated for a further five days. Finally, the culture medium was removed and washed with PBS. Subsequently, a solution of methanol and acetic acid (3:1 *v*/*v*) was added for five minutes, and then the cells were stained with methyl violet solution. The plates were washed with distilled water. After drying, they were photographed using the iBright™ FL1500 (ThermoFisher, Waltham, MA, USA) and analyzed using ImageJ 1.53 software.

### 2.7. Cell Morphology Assay

1.2 × 10^5^ A2780cis cells were added to each well of a twelve-well microplate. After incubating for 24 h in a 5% CO_2_ atmosphere, different concentrations of the compounds were added to the wells and the plates were incubated again for 48 h. Using a Nikon Eclipse TS 100 inverted microscope (Nikon, Minato, Tokyo) coupled to a Moticam digital camera (1.3 Megapixels; Moticam, Xiamen, China), the plate wells were photographed at 0, 24, and 48 h, and the morphological changes observed were analyzed.

For morphological analysis by fluorescence with Green Plasma and DAPI, 1 × 10^4^ cells/well of A2780cis were added to 96-well plates and incubated for 24 h. After this, the cells were treated with different concentrations of the compound and incubated for 48 h. Then the medium was removed and the cells were fixed with methanol for 5 min, and 50 μL of CellMasK Green Plasma membrane was added. After 5 min, 50 µL of DAPI was added and incubated at room temperature for a further 10 min. Images were taken using a fluorescence microscope (CELENA^®^ S Logos Biosystems, Anyang-si, Republic of Korea) with the appropriate filters.

### 2.8. Cell Viability (Kit: LIVE/DEAD^®^)

1.0 × 10^4^ A2780cis cells were added to each well in a 96-well plate. After 24 h, the cells were treated with different concentrations of the compound and incubated again for 48 h. Then 100 µL of the LIVE/DEAD^®^ solution prepared as described by the manufacturer was added. The images were acquired using a fluorescence microscope (CELENA^®^ S logos biosystems) with a 4× objective, carried out in triplicate. Live and dead cells were counted using ImageJ 1.53 software and the IC_50_ value was calculated using GraphPad Prism 8.0.2.

### 2.9. Cell Migration Assay—“Wound Healing”

4.0 × 10^5^ A2780cis cells were added to each well of a 12-well plate. The plate was incubated for 24 h and the formation of a monolayer of cells was observed. Using a micropipette tip, a scratch (wound) was made in the center of the well and culture medium and different compound solutions were added.

Using the digital camera attached to the microscope, the wells were photographed at 0, 24, and 30 h. The images obtained were analyzed using ImageJ software. It was then possible to calculate the rate of wound reduction in relation to time and the values obtained were analyzed using GraphPadPrism 8.0.2 software.

### 2.10. Cell Cycle Analysis

A2780cis cells (1 × 10^5^ cells/well) were seeded in 12-well plates. After incubation in CO_2_ atmosphere at 37 °C for 24 h, the cells were treated with different concentrations of complex **PdB1** for 24 h. Then, the cells were collected, washed with ice-cold PBS, and fixed in 70% ethanol at −20 °C for 24 h. After fixation, the cells were centrifuged at 2000 rpm and 4 °C for 5 min, resuspended in 150 μL of PBS buffer containing RNase A (0.2 mg/mL) and hypotonic fluorochrome solution (PI 5 μg/ mL), and incubated for 30 min before being analyzed on an Accuri C6 (BD Biosciences, Franklin Lakes, NJ, USA) flow cytometer that recorded 10,000 events. The cell cycle phase distribution was analyzed in triplicate by using the FlowJo 10.8 Software. Untreated cells were the negative control.

### 2.11. Apoptosis Assay

Apoptosis in A2780cis cells induced by the complexes was analyzed by flow cytometry; a PE-Annexin-V Apoptosis Detection kit (BD Biosciences) was employed. The cells (1 × 10^5^ cells/well) were seeded in a 24-well plate. After incubation for 24 h, the cells were exposed to increasing concentrations of complex **PdB1** for 24 h. After treatment, the cells were collected, centrifuged at 2000 rpm and 4 °C for 5 min, washed with ice-cold PBS, and resuspended in 150 µL of binding buffer. PE-Annexin V (2.5 µL) and 7ADD (2.5 µL) were added, which was followed by incubation in the dark at room temperature for 20 min. Then, the cells were centrifuged at 2000 rpm and 4 °C for 5 min, resuspended in 400 µL of binding buffer, and analyzed on an Accuri C6 (BD Biosciences) flow cytometer; 10,000 events were recorded. Fluorescence was quantified in triplicate with the Cell Quest software (BD Biosciences). Untreated cells were the negative control.

### 2.12. Three-Dimensional Assay

The 3D culture assay was performed with the A2780cis cell line using the Greiner Bio-One 96-well magnetic bioprinting kit. In total, 150 µL of magnetic nanoparticles were added to the cells grown in a 25 cm^2^ bottle and incubated for 24 h. Then, 3750 cells/well were added to repellent 96-well plates, taken to the magnetic drive, and incubated for 4 days for the formation of steroids. Once the spheroids had been obtained, they were treated with the compound **PdB1** at different concentrations. Finally, images of the spheroids were recorded at 0 and 48 h, and images with fluorescence markers (DAPI and PI) were also obtained for the 48 h period using a logos biosystems CELENA^®^ S fluorescence microscope.

## 3. Results and Discussion

### 3.1. Synthesis and Characterization

The **Pd(II)** complexes were synthesized using the substitution reaction of the ligands present in the precursor **[Pd(Cl)_2_(CH_3_CN)_2_]**, as shown in Scheme 1. Substitution of the acetonitriles coordinated to the **Pd(II)** ion occurs by an activated associative mechanism, in which the **TSC** ligand approaches the metal center via the axial position (d_z_^2^), and the penta-coordinated intermediate was formed, followed by the exit of CH_3_CN. After the first substitution, the ligand attacks the metal center again and substitutes the second acetonitrile, forming a five-membered chelated ring [22].

Once the acetonitrile ligands are replaced and the chelate ring is formed, the **palladium (II)** complexes form a bimetallic intermediate in the solution, which is constituted by the chelate rings formed and two clorides in bridge [27]. The intermediate is broken up with the addition of triphenylphosphine after 24 h of reaction.

According to the ^1^H NMR spectra of the complexes, the signals from the CH_3_ and CH_2_CH_3_ groups are found in the more protected region of the spectrum. The signals found in the region higher than 10 ppm refer to H-N(2) and H-N(3). The H-N(2) signal was the most acidic of the amines, and there was a shift from 8.37 ppm of the free ligand (**L-C3**) to 13.66 ppm of the complex (**PdC3**). Thus, coordination to the metal center represented a reduction in the electronic density around this bond.

Due to the great structural similarity between the **Pd(II)** compounds synthesized, a detailed evaluation was made of **PdC3**, which is the coordination compound with the greatest structural complexity in the series (all the other ^1^H NMR spectra are available in Figures S2–S7). The H signals of the aromatic rings were found between the signals of the vinyl hydrogens. In the **L-C3** ligand, the H(7) and H(8) signals were observed at 6.33 and 7.02 ppm, respectively; however, with coordination to **Pd(II)**, there was a shift in the electronic density of the ligand to supply the positive charge of the metal, generating a change in the chemical environment of the vinyl signals, which were shifted to 6.90 [H(7)] and 8.18 [H(8)] ppm. The ^1^H-^1^H COSY experiment was performed for the **PdC3** complex, allowing the observation of H–H correlations from both the **TSC** ligand and the triphenylphosphine coordinated to the metal center (Figure S8).

In the ^13^C{^1^H} NMR spectrum (100 MHz) obtained for the **PdC3** complex (Figure S9), it was possible to observe the signals for the aliphatic carbons at 13.69 and 40.27 ppm, which corresponded to the *N*-ethyl substituent of the **TSC** ligand. To identify the C17 signal, the DEPT-135 experiment was carried out (Figure S10). The signals referring to the carbons of the vinyl group (C7 and C8) were found at 119.70 and 150.18 ppm, and then the most unprotected signals were assigned to C9 (166.04 ppm) and C16 (207.12 ppm), which correspond to the carbons bonded directly to the N and S atoms, respectively, which participate in the coordination to the **Pd(II)** metal in the formation of the complex. The aromatic carbons are found in the 127 to 135 ppm region, where it was possible to identify the signals relating to the phenyl groups. Additionally, the ^1^H-^13^C-HSQC spectrum was obtained for the **PdC3** complex (Figure S11). The experiment corroborates the proposed structure.

The ^31^P{^1^H} NMR experiment was also carried out and the chemical shift values of the different complexes synthesized were similar to each other. There was a shift in the free PPh_3_ signal from −6.81 ppm to 30 ppm (Figure S12), indicating a reduction in the electronic density at the P of the triphenylphosphine due to coordination with **Pd(II)**. Also, the **PdB3** complex was tested for stability in DMSO. Using ^31^P{^1^H} NMR, the complex analyzed remained stable after 48 h and showed no change in chemical shift (Figure S13).

According to the infrared spectra (Figure S14), in the region between 3500 and 3200 cm^−1^, bands referring to the νN-H stretching are found. In the region of 3100 cm^−1^, the νC-H stretching bands of sp^2^ hybridization were assigned. Bands in the region below 3000 cm^−1^ were observed for all the complexes, with the exception of the **PdC1** complex, which has no carbons with sp^3^ hybridization. In the 1600–1400 cm^−1^ region, strong intensity bands were detected referring to the νC=C and νC=N stretches. The band at 1090 cm^−1^, referring to the asymmetric deformation τ(P-CPh), indicates the coordination of the **PPh_3_** ligand to the metal center. The band referring to the ν_S_C=S stretch was found in the free **TSC** ligands at 750 cm^−1^ and in the spectra of the complexes around 710 cm^−1^, indicating the weakening of the C=S bond [28]. In addition, the band referring to the νC=N stretch was strengthened, showing that the coordination of the **TSC** ligand to **Pd(II)** resulted in changes around the thioamide bonds.

The mass spectrometry technique verified the presence of the complexes in their cationic form, as well as detecting the cation related to the labilization of Cl^−^ and H^+^ (Figures S15–S20). Both cations detected showed the Pd isotopic pattern, with the two most intense peaks referring to the ^106^Pd and ^108^Pd isotopes, which is in line with the isotopic abundance described in the literature [29,30]. The errors (in ppm) calculated from the theoretical masses and the experimental results did not exceed 3 ppm, which is a clear indication that the proposed structures were obtained. The calculation was carried out for the most intense isotope of the molecular ion [M]^+^.

According to the XRD data, the complexes could be divided into those that crystallized in a cationic and neutral form, and all the structural representations are available in Figures S21–S26 and the crystallographic data in Tables S1–S3. In all the structures obtained, the distorted square-planar geometry around the metal center was observed, and the complexes that crystallized in the neutral form showed a shorter N(2)-C(10) bond distance and a longer S-C(10) bond distance, which was an expected change due to the deprotonation of N(2), which generates a change in the electronic density of the complexes and, consequently, an increase in the double bond character between N and C(10). The effects of deprotonation could be seen in the Pd-S bond, wherein the neutral species this value is less than 2.261 Å, and in the cationic species it has more elongated bonds (2.267–2.279 Å). In the **PdC3** system, the Pd-S bond difference between the neutral and protonated species was 0.036 Å.

**Figure 1:**
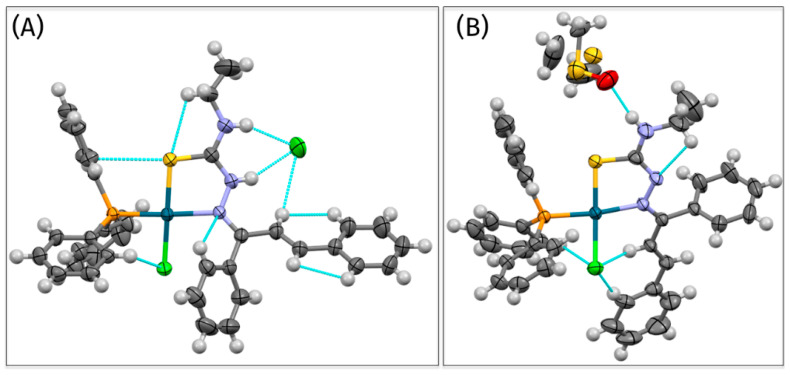
Three-dimensional structural representations of the **PdC3** complex obtained for the respective single crystals, ellipsoids of 50% thermal probability. (**A**) **PdC3-Cl** crystallized in acetonitrile/chloroform. (**B**) **PdC3-DMSO** crystallized in DMSO.

Single crystals of the **PdC3** complex were obtained in two different structures. The structure named **PdC3-Cl** was obtained from the reaction solution and had the **TSC** ligand as protonated and Cl^−^ as the counter-ion. In turn, the **PdC3-DMSO** structure had its single crystal obtained in a complex-DMSO solution and showed the **TSC** ligand deprotonated with the presence of the DMSO solvent in its three-dimensional structure and with the chloride ligand remaining bound to the **Pd(II)** complex. The crystallographic structures obtained for this same complex showed different conformations (Figure 1). This behavior is related to the characteristics of each solvent. The chloroform/acetonitrile solution is less basic than DMSO. Therefore, in DMSO, the complex deprotonates, while in the chloroform/acetonitrile solution it does not deprotonate. It is important to mention that this behavior does not influence the cytotoxicity of the compound, since the tests are carried out in buffered solutions.

### 3.2. Cell Tests

#### 3.2.1. Cell Viability (*IC*_50_)

The cytotoxic activity of the complexes was assessed against a range of tumor and non-tumor cell lines. For clarity, the cell viability results are grouped according to breast (Table 1), ovarian (Table 2), and lung and skin (Table 3) cell lines. The thiosemicarbazone ligands and **[PdCl_2_(MeCN)_2_]** were tested against the MDA-MB-231, A549, MRC5, and A2780cis cell lines. For these cell lines, the **TSC** ligands and **Pd(II)** precursor showed no cytotoxic effect at the tested concentrations, generally presenting *IC*_50_ values higher than 50 µmol·L^−1^.

**Figure 2:**
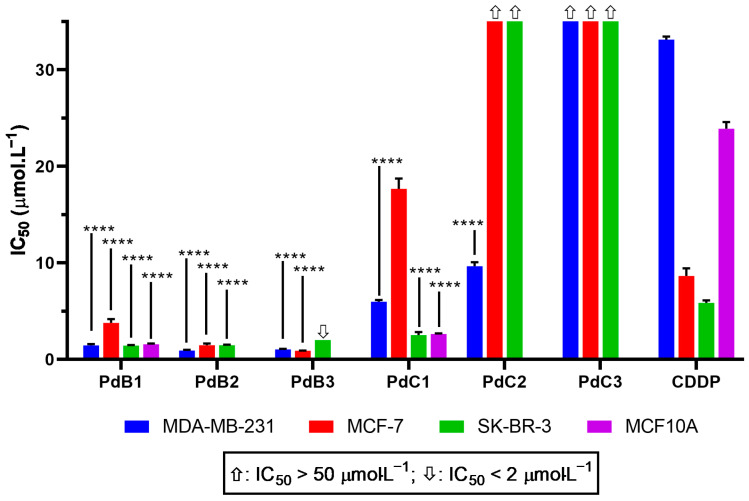
*IC*_50_ values (µmol·L^−1^) of **Pd(II)** complexes (**PdB1**, **PdB2**, **PdB3, PdC1**, **PdC2**, **PdC3**) and cisplatin (**CDDP**) against breast cancer cell lines MDA-MB-231, MCF-7, SK-BR-3, and the non-tumorigenic epithelial cell line MCF10A. Data are presented as mean ± standard deviation. Statistical analysis was performed using one-way ANOVA, comparing the *IC*_50_ values of each **Pd(II)** complex to the respective *IC*_50_ value of cisplatin (**CDDP**) within each cell line. Significance levels are indicated as **** *p* < 0.0001. Arrows indicate: ↑ *IC*_50_ > 50 µmol·L^−1^; ↓ *IC*_50_ < 2 µmol·L^−1^.

For breast cancer, the tumor cell lines MDA-MB-231, MCF-7, and SK-BR-3 were tested, along with the non-tumorigenic line MCF-10A, which was included only for the **PdB1** and **PdC1** complexes. Overall, the compounds demonstrated high cytotoxicity, with low *IC*_50_ values in most of the tested lines, except for those in the **C** series. Specifically, **PdC2** and **PdC3** were largely inactive in breast cancer lines, while **PdC1** exhibited significantly lower *IC*_50_ values compared to the standard drug cisplatin, except for the MCF-7 cell line (Figure 2).

Among all tested compounds, those in the **B** series were the most potent, consistently showing the lowest *IC*_50_ values towards both tumor and non-tumor breast cell lines. The compounds also overcome cisplatin, which was used as a reference drug. Notably, their activity against MDA-MB-231 and SK-BR-3 was constant despite structural variations in the **R_2_** substituent, suggesting that these changes had little effect on the efficacy of these lines. In contrast, for MCF-7 cells, increasing the size of the **R_2_** group increased activity, with **PdB3** yielding the lowest *IC*_50_ value in this line.

To further evaluate the potential of these complexes, their activity was also tested in ovarian cancer cell lines A2780 and A2780cis, the latter being resistant to cisplatin. This comparison aimed to determine whether resistance mechanisms affecting cisplatin would also impact the new compounds.

Interestingly, *IC*_50_ values in ovarian cell lines were generally lower than in other tumor types, indicating strong cytotoxic effects, as well as other recently reported palladium complexes [31,32,33]. The resistance factor (RF), calculated as *IC*_50_(A2780cis)/IC_50_(A2780), revealed that **B** series complexes were more effective against the non-resistant A2780 line (RF > 1), suggesting reduced efficacy in resistant cells. In contrast, the **C** series showed mixed behavior, with some compounds maintaining activity in both lines.

Within the **C** series, an inverse correlation emerged between the size (or molecular weight) of the **R_2_** substituent and cytotoxic activity: larger substituents led to higher *IC*_50_ values. On the other hand, the **B** complexes remained largely unaffected by changes to **R_2_**, exhibiting a more consistent cytotoxic profile.

Contrary to the strong activity observed in breast and ovarian cell lines, the complexes exhibited lower cytotoxicity against the A549 lung cancer cell line. Of the series tested, only the **B** family showed measurable *IC*_50_ values within the concentration range evaluated. Interestingly, an inverse trend was observed for A549 compared to the MCF-7 cell line: the complex with the largest **R_2_** substituent (**PdB3**) showed the highest activity and yielded the lowest *IC*_50_ within this series. Nevertheless, the overall *IC*_50_ values for A549 remained higher than those observed for other tumor models, indicating a generally reduced sensitivity.

For the non-tumor lung fibroblast line MRC-5, the cytotoxic profile followed previous trends, with the **B** series again showing superior activity. In this case, however, the lowest IC_50_ was observed for the complex with the smallest R_2_ group (**PdB1**), suggesting that bulkier substituents may reduce efficacy in non-tumor lung cells.

In addition, two representative complexes, **PdB1** and **PdC1**, were evaluated against the A375 melanoma cell line. Of the two, only **PdB1** had a significant effect on cell viability, further confirming the higher potency and broader spectrum of activity of the **B** compounds across multiple tumor types.

In summary, the evaluation of cytotoxic activity in various tumor and non-tumor cell lines revealed consistent trends in structure–activity relationships, particularly highlighting the superior performance of the **B**-Series. A key observation in multiple cell lines was the increased cytotoxicity associated with the presence of a terminal methyl group (-CH_3_) in the **R_2_** substituent. This is following the literature reports suggesting that methylation of bioactive molecules can enhance antiproliferative effects and inhibit cell migration, possibly by altering membrane permeability or interaction with intracellular targets [34,35,36].

To evaluate the therapeutic potential of the complexes, the selectivity index (SI) was calculated (Table 4) as the ratio between the *IC*_50_ values for the non-tumor MRC-5 line and those for the tumor cell lines. For synthetic organic and organometallic compounds, SI values greater than 10 are considered indicative of promising selectivity [37,38,39]. Notably, the **PdC1** complex exhibited exceptionally high SI values of 75.4 for the resistant A2780cis and 116.9 for the non-resistant A2780 line, suggesting strong selectivity for ovarian tumor cells.

Based on these findings, the **PdC1** complex was selected for further in-depth studies to elucidate its mechanism of action in the in vitro tumor microenvironment. The **PdB1** complex was also included in the subsequent experiments due to its close structural similarity to **PdC1** and its consistent cytotoxic profile, providing a complementary perspective to understand the influence of structural variations on biological activity.

#### 3.2.2. Clonogenic Assay

This assay allows evaluation of the effects of compounds on the long-term proliferative potential of cancer cells, specifically their ability to survive, divide, and form colonies after treatment. Colony formation assays reveal whether cells can recover and proliferate after exposure to compounds, thus providing insight into both cytotoxic and cytostatic effects.

**Figure 3:**
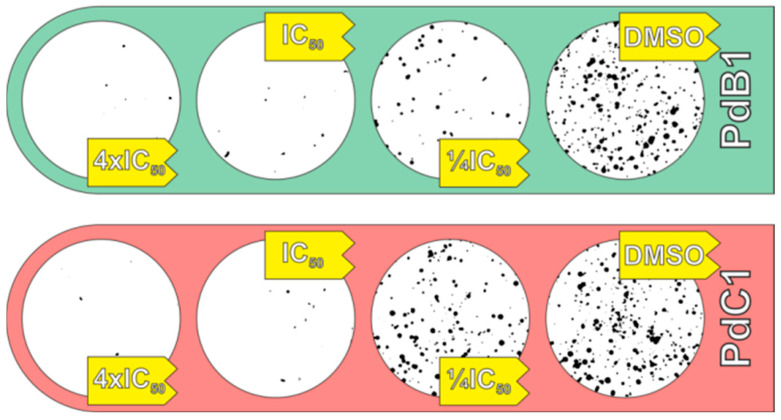
Images of the clonogenic assay carried out with A2780cis cells (600 cells/well); the cells were incubated in the presence of the complexes **PdB1** and **PdC1** at different concentrations for five days of treatment. Each circular area in the image corresponds to a 35 mm well of the 6-well plate.

**Figure 4:**
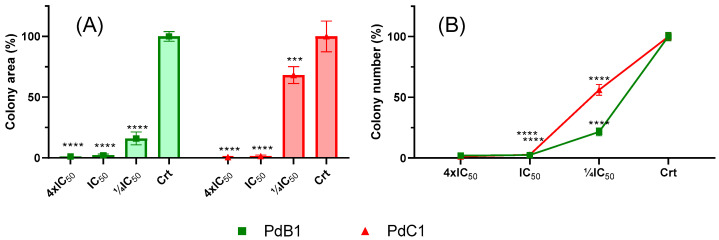
(**A**) Bar graph of the mean colony areas and (**B**) graph of the number of colonies observed for the A2780cis cell with the treatment of the compounds in concentration in relation to the *IC*_50_ of each complex (*** *p* < 0.001 and **** *p* < 0.0001).

In this context, both the **PdB1** and **PdC1** complexes exhibited a predominantly cytotoxic profile (Figure 3 and Figure 4). In particular, the **PdB1** complex significantly reduced colony formation even at sub-*IC*_50_ concentrations, suggesting that it interferes with essential cellular functions required for survival and regrowth. This suggests a potent effect not only on rapidly dividing cells, but potentially on the clonogenic potential of tumor cells, which is closely linked to tumor progression and recurrence.

The ability of **PdB1** to exert such inhibitory effects at lower concentrations may be due to its structural characteristics, including the presence of a terminal methyl group in the **R_2_** substituent, which has been associated with enhanced cellular uptake and interaction with biomolecular targets. **PdC1** also reduced colony formation, although to a lower extent, which is consistent with its lower activity in certain cell lines but still supports its cytotoxic potential.

These results reinforce the relevance of colony formation assays as a complement to *IC*_50_-based assessments and highlight the potential of **PdB1**, especially at lower, sublethal concentrations, as a candidate for further studies aimed at targeting tumor-cell persistence and resistance mechanisms.

#### 3.2.3. Cell Morphology Assay

Evaluation of cell morphology provides valuable insight into the effects of compounds on cell structure and viability. In this study, we used the A2780cis tumor cell line, which is characterized by an adherent growth pattern. Changes such as decreased cell density and the presence of cellular debris serve as morphological indicators of cytotoxicity and cell death.

**Figure 5:**
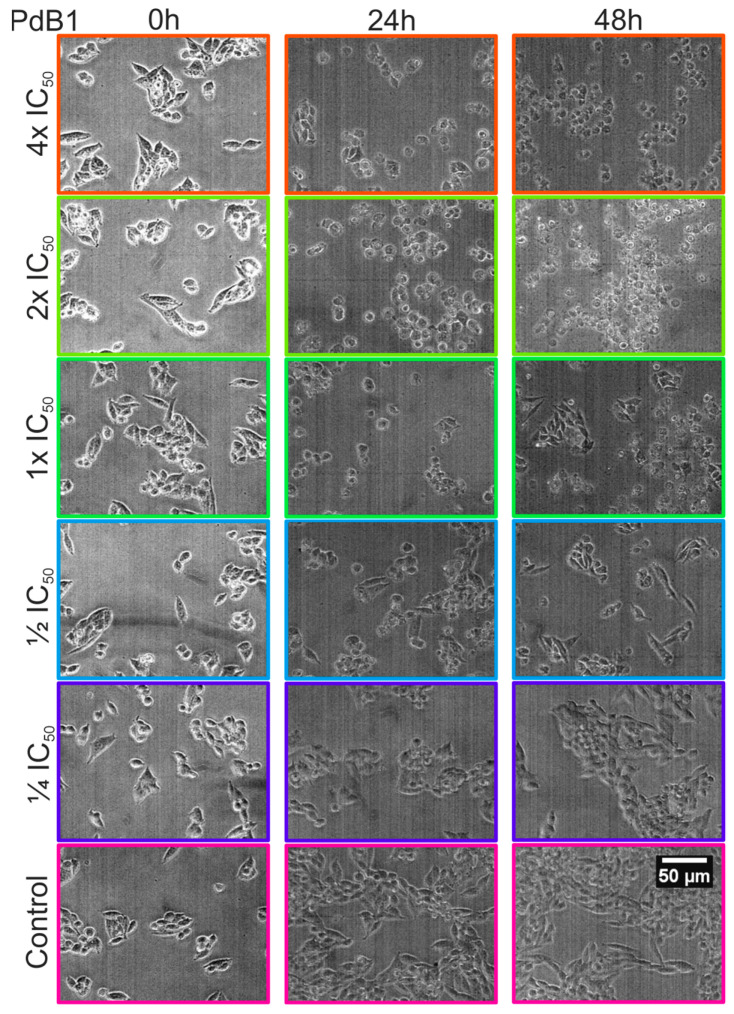
Microscopy images of the morphological evaluation assay for the **PdB1** complex at different concentrations in the A2780cis cell line and at different times.

Microscopic analysis of treated A2780cis cells revealed a marked cytotoxic response at concentrations above the *IC*_50_ of the compounds tested (Figure 5 and Figure S27). Specifically, we observed a significant reduction in the number of adherent cells, the appearance of rounded and detached cells often indicative of apoptosis or loss of membrane integrity, and an accumulation of cell fragments. These morphological changes strongly suggest that the compounds exert a potent cytotoxic effect at higher concentrations.

The correlation between increased compound concentration and the observed morphological changes reinforces the potential of these agents to disrupt cellular homeostasis and induce cell death.

In addition to the initial morphological evaluation, further analysis was performed using fluorescent staining to better characterize the cellular changes induced by the compound **PdB1**. Two markers were used: DAPI, which binds to DNA and highlights nuclear morphology, and Green Plasma, which stains the plasma membrane. This dual-staining approach allowed for more detailed observation of potential morphological changes associated with cell death mechanisms.

**Figure 6:**
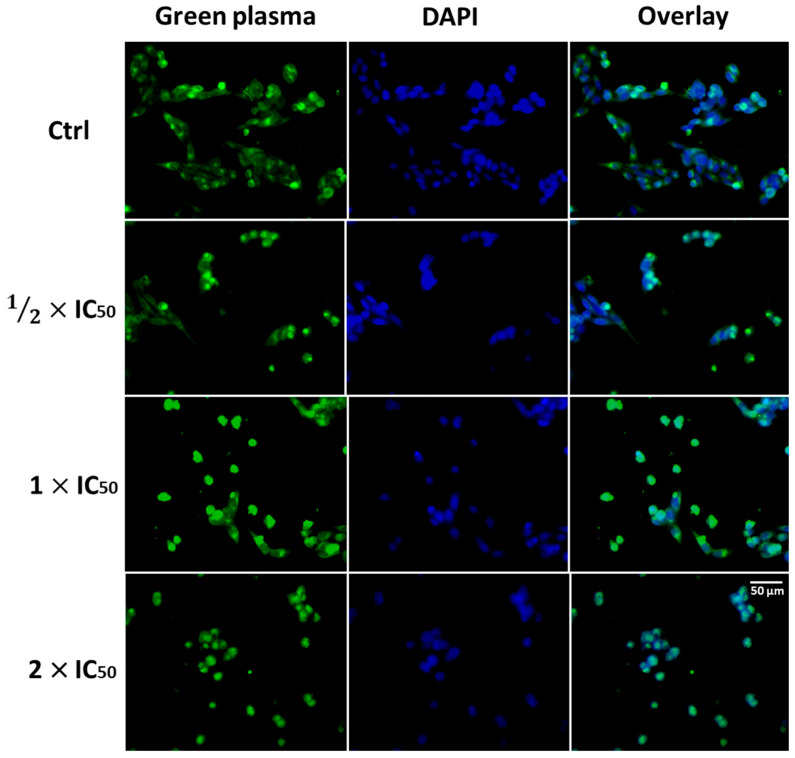
Fluorescence microscopy images using the fluorescent markers DAPI and Green Plasma to evaluate the morphology of the A2780cis cell line after 48 h of treatment with the complex **PdB1**, in different concentrations.

Following treatment of A2780cis cells with **PdB1**, starting at concentrations as low as ½ × *IC*_50_, a significant shift in cell morphology was observed (Figure 6). Green Plasma staining revealed a transition to a more spherical shape and a reduction in total cell area, suggesting membrane retraction and early apoptotic features. At the same time, DAPI staining showed pronounced nuclear changes, including chromatin condensation and nuclear fragmentation hallmarks of apoptosis. These morphological changes became increasingly evident with increasing concentrations of **PdB1**, supporting a dose-dependent induction of apoptosis.

#### 3.2.4. Cell Viability (Kit: LIVE/DEAD^®^)

To further investigate the cytotoxic effects of **PdB1** on A2780cis cells, viability was assessed using the LIVE/DEAD^®^ viability/cytotoxicity kit. This assay distinguishes between live and dead cells based on membrane integrity and esterase activity. Viable cells fluoresce green due to intracellular conversion of non-fluorescent calcein-AM to fluorescent calcein, while non-viable cells fluoresce red due to uptake of ethidium homodimer-1 (EthD-1), which binds to nucleic acids in cells with compromised membranes [40,41].

**Figure 7:**
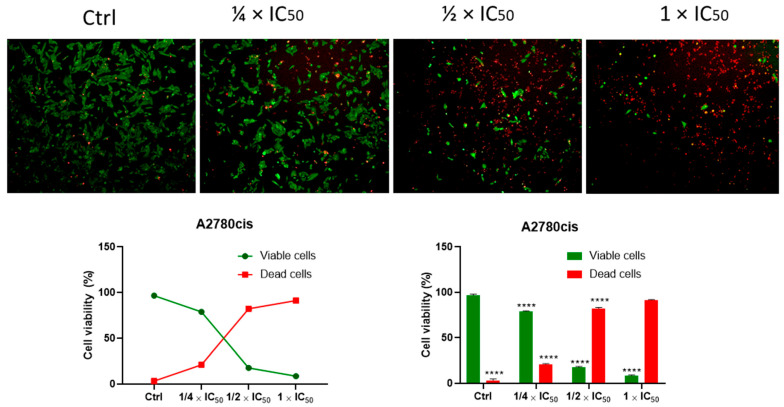
Fluorescence microscopy images of A2780cis cells treated with the compound **PdB1** and marked with the Kit: LIVE/DEAD after 48 h of treatment (green cells = viable cells and red cells = dead cells) and cell viability graphs (**** *p* < 0.0001). Images obtained using a CELENA^®^ S microscope with 4× zoom.

Fluorescence microscopy revealed a marked increase in red-fluorescing (non-viable) cells beginning at ½ × *IC*_50_, indicating early signs of cell death (Figure 7). This effect intensified with increasing concentrations of **PdB1**, suggesting a clear dose-dependent response.

Notably, the viability profile observed with the LIVE/DEAD^®^ assay closely matched the MTT assay results, reinforcing the reproducibility and reliability of the findings. These consistent results confirm the cytotoxic efficacy of **PdB1** against A2780cis cells.

Taken together with the morphological changes observed by both brightfield and fluorescence staining (DAPI and Green Plasma), the data support that **PdB1** induces apoptotic cell death, likely through mechanisms involving membrane disruption and nuclear condensation. In summary, these findings highlight the therapeutic potential of **PdB1** as a candidate for targeting cisplatin-resistant ovarian cancer cells.

#### 3.2.5. Cell Migration Assay (Wound Healing)

This experiment was designed to evaluate the ability of the **Pd(II)** complexes to inhibit cell migration using a wound healing assay. Cell migration was assessed by comparing the initial wound area with the area remaining after treatment. As expected, the control group treated with 0.5% (*v*/*v*) DMSO showed the highest degree of wound closure, indicating robust migratory activity in the absence of cytotoxic compounds.

**Figure 8:**
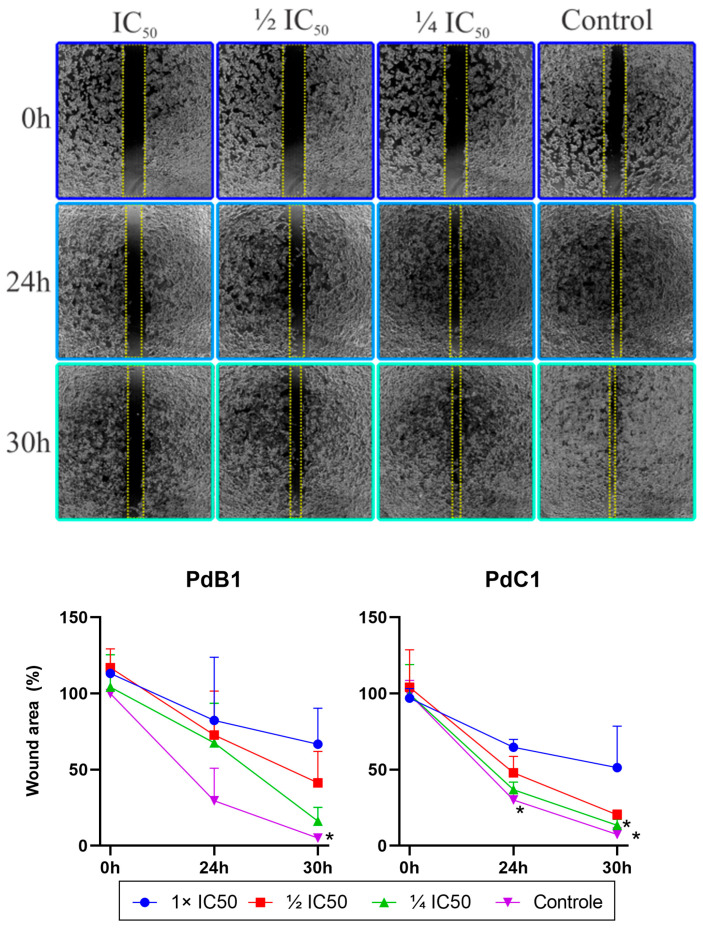
Microscopy images of the cell migration assay at times 0 h and 30 h after the addition of the compound **PdB1** at different concentrations in the A2780cis cell line, and graphs of wound area (%) versus time for each of the compounds tested in *IC*_50_ fractions (* *p* < 0.05). Images obtained using a Nikon Eclipse TS 100 microscope with 4× zoom.

In contrast, cells treated with **Pd(II)** complexes showed a marked reduction in wound healing, suggesting an impaired migratory capacity (Figure 8). The inhibitory effect on migration was most pronounced at concentrations near *IC*_50_, where approximately 50% of the wound area remained unhealed. This observation suggests that the compounds not only exert cytotoxic effects, but also interfere with cellular mechanisms involved in migration, a key factor in cancer invasion and metastasis.

#### 3.2.6. Cell Cycle Analysis

Cell cycle analysis was performed to determine whether treatment with the **Pd(II)** compounds leads to alterations in cell cycle distribution or induction of cell death in A2780cis cells. Experiments were performed at concentrations corresponding to *IC*_50_.

**Figure 9:**
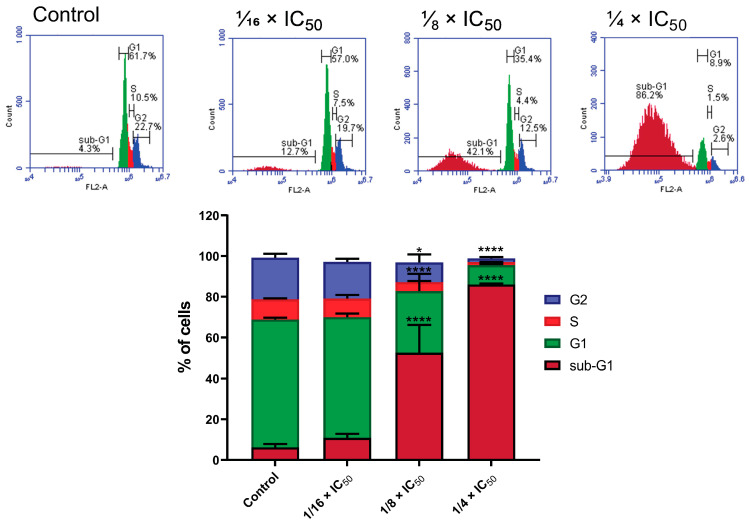
Graphs of the dispersion of cells in different phases of the cell cycle for different concentrations of the **PdB1** complex, and a bar graph of the percentages obtained from the dispersions at each concentration of the PdB1 complex. A2780cis cell line (* *p* < 0.05 and **** *p* < 0.0001).

The results (Figure 9) showed a clear accumulation of cells in the sub-G1 phase after treatment. This phase is not part of the normal cell cycle, but reflects a population of cells with fragmented DNA, which is often associated with apoptosis. Thus, the increase in sub-G1 does not indicate classical cell cycle arrest, but instead the presence of dead or dying cells [42].

These results suggest that the primary effect of the **Pd(II)** compounds at these concentrations is the induction of cell death via apoptosis rather than the interruption of specific cell cycle checkpoints. This is consistent with other assays performed in this study that indicate apoptotic features, further supporting the cytotoxic potential of the compounds.

#### 3.2.7. Apoptosis Assay by Anexin V

To further investigate the mechanism of **PdB1**-induced cell death, an apoptosis assay was performed using the Annexin V-FITC/PI kit followed by flow cytometry analysis. This approach allows differentiation between live, early apoptotic, late apoptotic, and necrotic cells based on phosphatidylserine exposure and membrane integrity.

**Figure 10:**
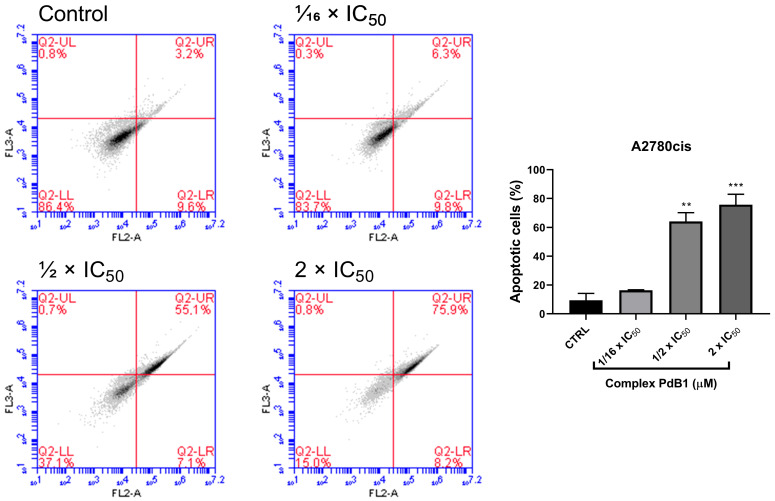
Histogram of the distribution of cell death of A2780cis cells after treatment with different concentrations of the compound **PdB1**, and a graph of the percentage of total cell death (** *p* < 0.01 and *** *p* < 0.001).

Analyzing Figure 10, it is observed that in the control group (treated with 0.5% DMSO), the majority of the cell population (86.4%) was located in Q3, indicating a predominantly viable population with intact membranes and no phosphatidylserine externalization. However, after treatment with PdB1 at ½ × *IC*_50_, a significant reduction in viable cells was observed, with only 37.1% remaining in Q3. At the same time, there was a significant increase in the Q2 quadrant, indicating cells undergoing late apoptosis.

This dose-dependent shift in the apoptotic profile suggests that **PdB1** promotes programmed cell death rather than necrosis. The relatively modest presence of cells in Q1 (necrosis) further supports this conclusion. The increase in the Q2 population at sub-lethal concentrations also suggests that **PdB1** initiates a time-dependent or progressive apoptotic process that culminates in late-stage apoptosis as exposure continues.

These results are consistent with previous findings from sub-G1 cell cycle analysis and DAPI staining, which demonstrated nuclear condensation and DNA fragmentation, both typical features of apoptosis. Taken together, these data confirm that apoptosis is the primary mode of cell death induced by **PdB1** and support its potential as a pro-apoptotic agent against cisplatin-resistant ovarian cancer cells.

#### 3.2.8. Three-Dimensional Assay

The morphological assay in 3D cell culture was performed using the A2780cis tumor cell line to evaluate structural changes induced by the **PdB1** complex in a more physiologically relevant and drug-resistant tumor model. Unlike traditional 2D monolayer cultures, 3D spheroid models better mimic the in vivo tumor microenvironment by promoting cell–cell and cell–matrix interactions, as well as gradients of oxygen, nutrients, and drug penetration. This model is particularly useful for evaluating compounds targeting chemoresistant cancers [43,44,45].

**Figure 11:**
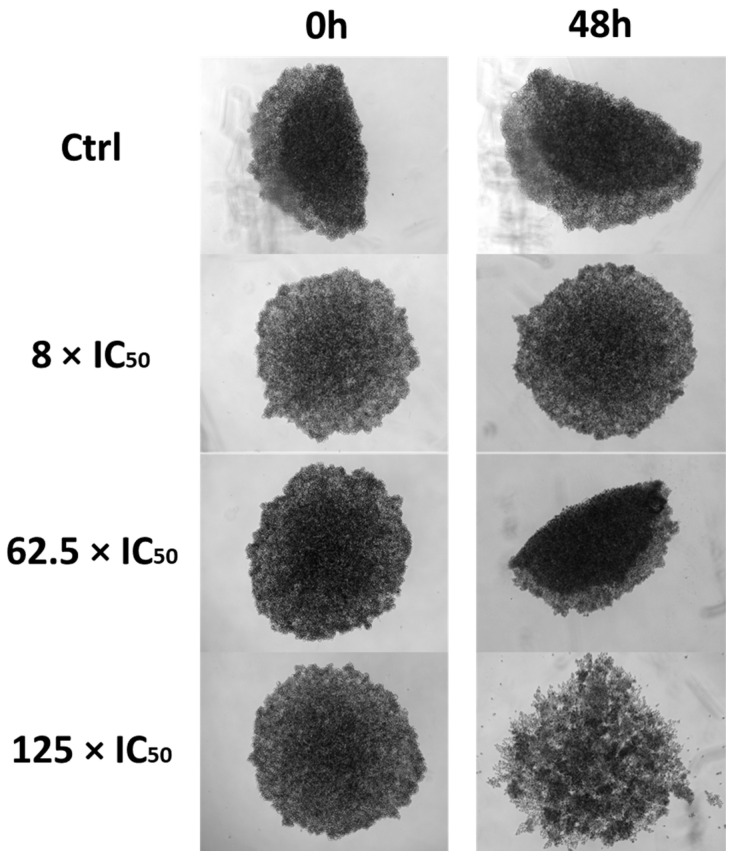
Microscopy images of the spheroid morphology of A2780cis cells treated with the **PdB1** complex after 48 h at concentrations of 1.6 µmol·L^−1^, 12.5 µmol·L^−1^, and 25 µmol·L^−1^, which correspond to approximately 8 × *IC*_50_, 62.5 × *IC*_50_, and 125 × *IC*_50_, respectively (*IC*_50_ = 0.20 µmol·L^−1^ for this cell line). Images were acquired using a CELENA^®^ S microscope with 4× magnification.

Brightfield microscopy images taken after 48 h of treatment showed continued spheroid growth at lower concentrations of **PdB1**, indicating limited cytotoxicity under these conditions (Figure 11). However, significant morphological changes were observed at a concentration of 25 µM. The spheroids showed a marked reduction in compaction and cohesion, with disrupted borders and visible disintegration of the spheroid structure. These features are indicative of loss of cell–cell adhesion and structural integrity, often associated with cell death within the spheroid core and periphery.

Such changes suggest that **PdB1** is able to penetrate the dense 3D architecture and exert cytotoxic effects even under conditions that typically confer resistance to conventional treatments such as cisplatin. The morphological disruption observed at 25 µM highlights the potential of **PdB1** to overcome microenvironment-mediated resistance mechanisms.

**Figure 12:**
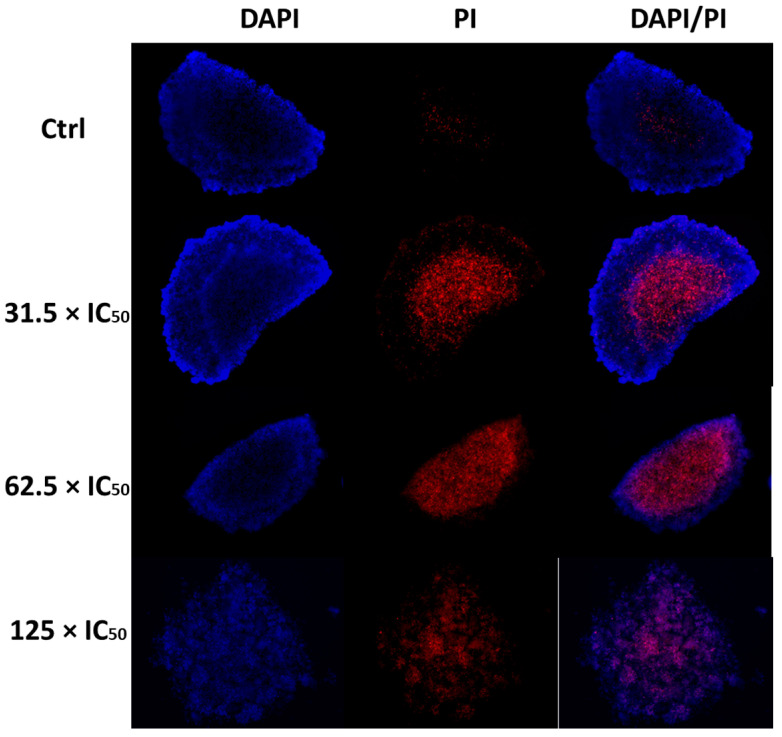
Fluorescence microscopy images of the spheroids of A2780cis cells treated with the **PdB1** complex after 48 h, using DAPI and PI staining. The tested concentrations were 6.3 µmol·L^−1^, 12.5 µmol·L^−1^, and 25 µmol·L^−1^, corresponding to approximately 31.5 × *IC*_50_, 62.5 × *IC*_50_, and 125 × *IC*_50_, respectively (*IC*_50_ = 0.12 µmol·L^−1^ for this cell line). Images were acquired using a CELENA^®^ S microscope with 4× magnification.

Fluorescence staining with DAPI and propidium iodide (PI) was used to further evaluate the extent and spatial distribution of cell death within A2780cis spheroids following treatment with the **PdB1** complex (Figure 12). In this assay, DAPI selectively binds to DNA and labels the nuclei of all cells with blue fluorescence, while PI is membrane-impermeable and selectively labels the nuclei of dead cells with compromised membrane integrity with red fluorescence. The overlap of the two signals results in a purple hue, indicating nuclei of cells that have undergone cell death.

Fluorescence microscopy images revealed a clear dose-dependent response to **PdB1** treatment. At lower concentrations, DAPI staining was predominant, indicating a higher proportion of intact, viable cells. In contrast, at 12.5 µM, the presence of strong PI fluorescence indicated a significant level of cell death within the spheroids, particularly around the outer layers where drug penetration is initially more effective. At the highest concentration tested (25 µM), the spheroids showed complete disintegration, accompanied by intense violet staining resulting from the co-localization of DAPI and PI signals. This strongly suggests that **PdB1** at this concentration induces widespread and possibly complete cell death within the 3D structure.

In particular, the diffuse PI staining and internal labeling of the spheroids demonstrate that **PdB1** has effective permeability, allowing it to reach deeper cell layers within the tumor-like structure. However, when compared to 2D monolayer assays such as MTT, it is evident that higher concentrations are required to induce similar cytotoxic effects in 3D culture, underscoring the increased resistance of spheroids due to factors such as limited drug diffusion, altered cell metabolism, and enhanced survival pathways.

## 4. Conclusions

In this study, new **Pd(II)** complexes were successfully proposed, synthesized, and characterized. The structures of most compounds were elucidated, and single crystals were obtained. Cellular assays revealed that the **B**-family complexes were more toxic than those of the **C** family, with the **R1** substituent having a greater influence on cell viability, while **R2** modulated cytotoxicity depending on the cell lineage. The **R1** group played a crucial role in determining the cytotoxic and cytostatic profiles, with aldehydes exhibiting a cytostatic effect near the *IC*_50_ and ketones displaying a cytotoxic profile. The carbonyl group modulated cytotoxic activity, with smaller aldehydes and ketones increasing cytotoxicity, while bulkier substituents reduced this effect. The complexes also demonstrated antimigratory potential, the ability to alter cell morphology, and apoptosis induction. The 3D assay indicated that the **PdB1** complex effectively induces cell death in physiologically relevant tumor environments, highlighting its potential as a promising candidate in the search for new metallodrugs.

## Figures and Tables

**Table 1:** *IC*_50_ values (µmol·L^−1^) of the complexes in 48 h of incubation for the human breast cell lines MDA-MB-231, MCF-7, SK-BR-3, and MFC-10A.

R_1_	R_2_	Complexes	MDA-MB-231	MCF-7	SK-BR-3	MFC-10A
	H	**PdB1**	1.45 ± 0.1	3.79 ± 0.4	1.42 ± 0.07	1.58 ± 0.07
Me	Me	**PdB2**	0.92 ± 0.08	1.47 ± 0.2	1.48 ± 0.06	―
	Et	**PdB3**	1.04 ± 0.06	0.91 ± 0.008	<2	―
	H	**PdC1**	5.97 ± 0.1	17.68 ± 1	2.25 ± 0.3	2.64 ± 0.06
Ph	Me	**PdC2**	9.66 ± 0.4	>50	>50	―
	Et	**PdC3**	>50	>50	>50	―
		Cisplatin	33.14 ± 0.3	8.64 ± 0.8	5.86 ± 0.2	23.90 ± 0.7

Abbreviations: Me (methyl), Et (ethyl), and Ph (phenyl).

**Table 2:** *IC*_50_ values (µmol·L^−1^) of the complexes in 48 h of incubation for the ovarian tumor cell lines A2780 and A2780cis, with the resistance factor (RF) value.

R_1_	R_2_	Complexes	A2780	A2780cis	RF
	H	**PdB1**	0.12 ± 0.01	0.20 ± 0.01	1.66
Me	Me	**PdB2**	0.13 ± 0.01	0.21 ± 0.01	1.62
	Et	**PdB3**	0.23 ± 0.01	0.26 ± 0.01	1.13
	H	**PdC1**	0.40 ± 0.01	0.62 ± 0.01	1.55
Ph	Me	**PdC2**	3.93 ± 0.2	1.66 ± 0.01	0.42
	Et	**PdC3**	22.78 ± 1	11.1 ± 0.6	0.48
		Cisplatin	14.24 ± 0.05	25.09 ± 0.9	1.76

**Table 3:** *IC*_50_ values (µmol·L^−1^) of the complexes in 48 h of incubation for A549, MRC5, and A375 cell lines.

R_1_	R_2_	Complexes	A549	MRC5	A375
	H	**PdB1**	44.8 ± 2	1.70 ± 0.2	1.34 ± 0.08
Me	Me	**PdB2**	30.4 ± 0.4	6.85 ± 0.3	―
	Et	**PdB3**	12.99 ± 1	9.28 ± 0.3	―
	H	**PdC1**	>50	46.77 ± 2	>50
Ph	Me	**PdC2**	>50	>50	―
	Et	**PdC3**	>50	>50	―
		Cisplatin	13.01 ± 0.4	21.61 ± 0.8	12.21 ± 0.62

**Table 4:** Selectivity index (SI *) in relation to the non-tumor cell line MRC5.

Complexes	MDA-MB-231	MCF-7	SK-BR-3	A2780	A2780cis	A549
**PdB1**	1.2	0.4	1.2	14.2	8.5	0.0
**PdB2**	7.4	4.7	4.6	52.7	32.6	0.2
**PdB3**	8.9	10.2	―	40.3	35.7	0.7
**PdC1**	7.8	2.6	20.8	116.9	75.4	―
Cisplatin	0.7	2.5	3.7	1.5	0.9	1.7

* SI = [IC_50_ of non-tumor cell line]/[IC50 of tumor cell lines].

## Data Availability

The original contributions presented in this study are included in the article/Supplementary Material. Further inquiries can be directed to the corresponding author.

## Supplementary Materials

The following supporting information can be downloaded at https://www.mdpi.com/article/10.3390/pharmaceutics17070829/s1, Figure S1: Structural proposal for Pd(II) complexes; Figures S2–S7: ^1^H NMR spectrum; Figure S8: ^1^H-^1^H COSY; Figure S9: ^13^C NMR spectrum; Figure S10: ^13^C DEPT-135 NMR spectrum; Figure S11: ^1^H-^13^C-HSQC spectrum; Figures S12–S13: ^31^P{ ^1^H} NMR spectra; Figure S14: FTIR vibrational spectrum; Figures S15–S19: Amplified mass spectrum; Figures S20–S25: Structural representation by X-ray diffraction; Figures S26–S64: Dose-response IC_50_ curve; Figures S65–S69: IC_50_ values (µmol·L^−1^); Figure S70: Microscopy images of the morphological evaluation assay; Figure S71: Microscopy images of the cell migration assay; Table S1: Values of the main distances and bond angles of the structures obtained; Tables S2–S3: X-Ray crystallographic data collection and refinement parameters.

## References

[1] Nenclares P., Harrington K.J. (2020). The biology of cancer. Medicine.

[2] Sarkar S., Horn G., Moulton K., Oza A., Byler S., Kokolus S., Longacre M. (2013). Cancer development, progression, and therapy: An epigenetic overview. Int. J. Mol. Sci..

[3] Kennedy S.R., Zhang Y., Risques R.A. (2019). Cancer-Associated Mutations but No Cancer: Insights into the Early Steps of Carcinogenesis and Implications for Early Cancer Detection. Trends Cancer.

[4] Bukowski K., Kciuk M., Kontek R. (2020). Mechanisms of Multidrug Resistance in Cancer Chemotherapy. Int. J. Mol. Sci..

[5] Debela D.T., Muzazu S.G.Y., Heraro K.D., Ndalama M.T., Mesele B.W., Haile D.C., Kitui S.K., Manyazewal T. (2021). New Approaches and Procedures for Cancer Treatment: Current Perspectives. SAGE Open Med..

[6] Chabner B.A., Roberts T.G. (2005). Chemotherapy and the War on Cancer. Nat. Rev. Cancer.

[7] Kelland L. (2007). The Resurgence of Platinum-Based Cancer Chemotherapy. Nat. Rev. Cancer.

[8] Ghosh S. (2019). Cisplatin: The First Metal Based Anticancer Drug. Bioorg. Chem..

[9] Barabas K., Milner R., Lurie D., Adin C. (2008). Cisplatin: A Review of Toxicities and Therapeutic Applications. Vet. Comp. Oncol..

[10] Rocha C.R.R., Silva M.M., Quinet A., Cabral-Neto J.B., Menck C.F.M. (2018). DNA Repair Pathways and Cisplatin Resistance: An Intimate Relationship. Clinics.

[11] Oun R., Moussa Y.E., Wheate N.J. (2018). The Side Effects of Platinum-Based Chemotherapy Drugs: A Review for Chemists. Dalton Trans..

[12] Lucaciu R.L., Hangan A.C., Sevastre B., Oprean L.S. (2022). Metallo-Drugs in Cancer Therapy: Past, Present and Future. Molecules.

[13] Casini A., Pöthig A. (2024). Metals in Cancer Research: Beyond Platinum Metallodrugs. ACS Cent. Sci..

[14] Zaki M., Arjmand F., Tabassum S. (2016). Current and Future Potential of Metallo Drugs: Revisiting DNA-Binding of Metal Containing Molecules and Their Diverse Mechanism of Action. Inorganica Chim. Acta.

[15] Fanelli M., Formica M., Fusi V., Giorgi L., Micheloni M., Paoli P. (2016). New Trends in Platinum and Palladium Complexes as Antineoplastic Agents. Coord. Chem. Rev..

[16] Czarnomysy R., Radomska D., Szewczyk O.K., Roszczenko P., Bielawski K. (2021). Platinum and Palladium Complexes as Promising Sources for Antitumor Treatments. Int. J. Mol. Sci..

[17] Azzouzi A.R., Vincendeau S., Barret E., Cicco A., Kleinclauss F., van Der Poel H.G., Stief C.G., Rassweiler J., Salomon G., Solsona E. (2017). Padeliporfin Vascular-Targeted Photodynamic Therapy versus Active Surveillance in Men with Low-Risk Prostate Cancer (CLIN1001 PCM301): An Open-Label, Phase 3, Randomised Controlled Trial. Lancet Oncol..

[18] Azzouzi A., Barret E., Moore C.M., Villers A., Allen C., Scherz A., Muir G., De Wildt M., Barber N.J., Lebdai S. (2013). TOOKAD^®^ S Oluble Vascular-targeted Photodynamic (VTP) Therapy: Determination of Optimal Treatment Conditions and Assessment of Effects in Patients with Localised Prostate Cancer. BJU Int..

[19] Gupta S., Singh N., Khan T., Joshi S. (2022). Thiosemicarbazone Derivatives of Transition Metals as Multi-Target Drugs: A Review. Results Chem..

[20] de Siqueira L.R.P., de Moraes Gomes P.A.T., de Lima Ferreira L.P., de Melo Rêgo M.J.B., Leite A.C.L. (2019). Multi-Target Compounds Acting in Cancer Progression: Focus on Thiosemicarbazone, Thiazole and Thiazolidinone Analogues. Eur. J. Med. Chem..

[21] Taylor S.E., Behr S., Cooper K.L., Mahdi H., Fabian D., Gallion H., Ueland F., Vargo J., Orr B., Girda E. (2025). Dose Finding, Bioavailability, and PK-PD of Oral Triapine with Concurrent Chemoradiation for Locally Advanced Cervical Cancer and Vaginal Cancer (ETCTN 9892). Cancer Chemother. Pharmacol..

[22] Lima M.A., Costa V.A., Franco M.A., de Oliveira G.P., Deflon V.M., Rocha F.V. (2020). Palladium (II) Complexes Bearing Thiosemicarbazone and Phosphines as Inhibitors of DNA-Topoisomerase II Enzyme: Synthesis, Characterizations and Biological Studies. Inorg. Chem. Commun..

[23] Lima M.A. (2023). Estudo da Atividade Anticâncer In Vitro para Complexos de Pd(II): Avaliação dos Substituintes dos Ligantes Tiossemicarbazonas. Ph.D. Thesis.

[24] Silva L.T.P., Pereira G.B.S., de Oliveira G.P., Lima M.A., de Araujo-Neto J.H., Akinyemi A.O., Vieira M.A., Nascimento-Júnior N.M., de Farias R.L., Ellena J.A. (2024). Synthesis, Characterization, Cytotoxicity Study, Interaction with DNA and Topoisomerase IIα of Square-Planar Complexes with Thiosemicarbazones. Polyhedron.

[25] Sheldrick G.M. (2015). Crystal structure refinement with *SHELXL*. Acta Crystallogr. Sect. C Struct. Chem..

[26] Sheldrick G.M. (2015). SHELXT–Integrated Space-Group and Crystal-Structure Determination. Acta Crystallogr. Sect. A Found. Adv..

[27] Quiroga A.G., Ranninger C.N. (2004). Contribution to the SAR field of metallated and coordination complexes: Studies of the palladium and platinum derivatives with selected thiosemicarbazones as antitumoral drugs. Coord. Chem. Rev..

[28] Farias R.L., Polez A.M.R., Silva D.E.S., Zanetti R.D., Moreira M.B., Batista V.S., Reis B.L., Nascimento-Júnior N.M., Rocha F.V., Lima M.A. (2021). In Vitro and in Silico Assessment of Antitumor Properties and Biomolecular Binding Studies for Two New Complexes Based on NiII Bearing K2N,S-Donor Ligands. Mater. Sci. Eng. C.

[29] Bakker J.M., Besson T., Lemaire J., Scuderi D., Maître P. (2007). Gas-Phase Structure of a π-Allyl-Palladium Complex: Efficient Infrared Spectroscopy in a 7 T Fourier Transform Mass Spectrometer. J. Phys. Chem. A.

[30] Kostyukovich A.Y., Burykina J.V., Eremin D.B., Ananikov V.P. (2021). Detection and Structural Investigation of Elusive Palladium Hydride Intermediates Formed from Simple Metal Salts. Inorg. Chem..

[31] Oliveira G.P., Lima M.A., Pereira G.B.S., Costa A.R., Batista A.A., Forim M.R., Cominetti M.R., Zanetti R.D., Farias R.L., Netto A.V.G. (2025). Thiophene-Based Thiosemicarbazones: Synthesis, Properties, and Anticancer Studies. J. Mol. Struct..

[32] Dutra J.L., Honorato J., Graminha A., Moraes C.A.F., de Oliveira K.T., Cominetti M.R., Castellano E.E., Batista A.A. (2024). Pd (II)/Diphosphine/Curcumin Complexes as Potential Anticancer Agents. Dalt. Trans..

[33] Tonon G., Mauceri M., Cavarzerani E., Piccolo R., Santo C., Demitri N., Orian L., Nogara P.A., Rocha J.B.T., Canzonieri V. (2024). Unveiling the Promising Anticancer Activity of Palladium(II)–Aryl Complexes Bearing Diphosphine Ligands: A Structure–Activity Relationship Analysis. Dalton Trans..

[34] Miyanaga S., Sakurai H., Saiki I., Onaka H., Igarashi Y. (2010). Anti-Invasive and Anti-Angiogenic Activities of Naturally Occurring Dibenzodiazepine BU-4664L and Its Derivatives. Bioorg. Med. Chem. Lett..

[35] Dao P., Jarray R., Le Coq J., Lietha D., Loukaci A., Lepelletier Y., Hadj-Slimane R., Garbay C., Raynaud F., Chen H. (2013). Synthesis of Novel Diarylamino-1, 3, 5-Triazine Derivatives as FAK Inhibitors with Anti-Angiogenic Activity. Bioorg. Med. Chem. Lett..

[36] Chang T.T., More S.V., Lu I.H., Hsu J.C., Chen T.J., Jen Y.C., Lu C.K., Li W.S. (2011). Isomalyngamide A, A-1 and Their Analogs Suppress Cancer Cell Migration In Vitro. Eur. J. Med. Chem..

[37] de Oliveira P.F., Alves J.M., Damasceno J.L., Oliveira R.A.M., Dias H.J., Crotti A.E.M., Tavares D.C. (2015). Cytotoxicity Screening of Essential Oils in Cancer Cell Lines. Rev. Bras. Farmacogn..

[38] Kim K., Yoo H.J., Jung J.H., Lee R., Hyun J.K., Park J.H., Na D., Yeon J.H. (2020). Cytotoxic Effects of Plant Sap-Derived Extracellular Vesicles on Various Tumor Cell Types. J. Funct. Biomater..

[39] Segun P.A., Ogbole O.O., Ismail F.M.D., Nahar L., Evans A.R., Ajaiyeoba E.O., Sarker S.D. (2019). Resveratrol derivatives from *Commiphora africana* (A. Rich.) Endl. display cytotoxicity and selectivity against several human cancer cell lines. Phyther Res..

[40] Sena-Lopes Â., Remião M.H., Alves M.S.D., da Rocha Fonseca B., Seixas F.K., Collares T., Borsuk S. (2020). Cell Viability Analysis of Toxocara Cati Larvae with LIVE/DEAD^®^ Viability/Cytotoxicity Kit. Exp. Parasitol..

[41] Demirbuga M., Wink D.J., Milligan R.D. (2024). Introduction to Fluorescence in General Chemistry Using the Intercalation of Propidium Iodide with DNA. J. Chem. Educ..

[42] Darzynkiewicz Z., Zhao H., Halicka H.D., Rybak P., Dobrucki J., Wlodkowic D. (2012). DNA Damage Signaling Assessed in Individual Cells in Relation to the Cell Cycle Phase and Induction of Apoptosis. Crit. Rev. Clin. Lab. Sci..

[43] Brajša K., Trzun M., Zlatar I., Jelić D. (2016). Three-Dimensional Cell Cultures as a New Tool in Drug Discovery. Period. Biol..

[44] Souza A.G., Silva I.B.B., Campos-Fernandez E., Barcelos L.S., Souza J.B., Marangoni K., Goulart L.R., Alonso-Goulart V. (2018). Comparative Assay of 2D and 3D Cell Culture Models: Proliferation, Gene Expression and Anticancer Drug Response. Curr. Pharm. Des..

[45] Foglietta F., Serpe L., Canaparo R. (2021). The Effective Combination between 3D Cancer Models and Stimuli-Responsive Nanoscale Drug Delivery Systems. Cells.

