# Supplementary material for: Enhanced Anticancer Potential of Pd(II)-Thiosemicarbazone Complexes: Selectivity, Mechanisms, and 3D Models
Source: Pharmaceutics. 2025 Jun 25;17(7):829. doi: 10.3390/pharmaceutics17070829 (PMC12297887; doi:10.3390/pharmaceutics17070829)
Supplement: Supplementary file 1 [file pharmaceutics-17-00829-s001.zip › pharmaceutics-3623117-supplementary.pdf]

Supporting Information

Enhanced Anticancer Potential of Pd(II)-Thiosemicarbazone Complexes: Selectivity, Mechanisms, and 3D Models

Mauro A. Lima [1,‡], Tamara Teixeira [1,‡], Dário B. Fortaleza [1], George B. S. Pereira [1], Amos O. Akinyemi [2], Carlos André Ferreira Moraes[1], Moacir R Forim [1], Alzir A. Batista [1], Jocely L. Dutra [1], Marcia R. Cominetti [3], João H. Araujo-Neto [4], Javier A. Ellena [5] and Fillipe V. Rocha [1,*]

[1] Departamento de Química, Universidade Federal de São Carlos, São Carlos, São Paulo, CEP 13565-905, São Carlos, São Paulo, Brazil

[2] Department of Toxicology and Cancer Biology, College of Medicine, University of Kentucky, Lexington, KY 40536, USA;

[3] Departamento de Gerontologia, Universidade Federal de São Carlos, São Carlos, São Paulo, CEP 13561-901 São Carlos, São Paulo, Brazil

[4] Departamento de Química Fundamental, Instituto de Química, Universidade de São Paulo, 05508-000, São Paulo, São Paulo, Brazil

[5] Instituto de Física de São Carlos, Universidade de São Paulo, São Carlos, São Paulo, CEP 13566-590, São Carlos, São Paulo, Brazil

[*] Correspondence: fillipe@ufscar.br

[‡] These authors contributed equally to this work.

List of Figures

Figure S1. Structural proposal for Pd(II) complexes. 11
Figure S2. ^{1}H NMR spectrum (400 MHz) in *$CDCl_3$* for the PdB1 complex........ 12
Figure S3. ^{1}H NMR spectrum (400 MHz) in *$CDCl_3$* for the PdB3 complex........ 12
Figure S4. ^{1}H NMR spectrum (400 MHz) in *$CDCl_3$* for the PdC1 complex. 13
Figure S5. ^{1}H NMR spectrum (400 MHz) in *$CDCl_3$* for the PdC2 complex. 13
Figure S6. ^{1}H NMR spectrum (400 MHz) in *$CDCl_3$* for the PdC3 complex. 14
Figure S7. ^{1}H NMR spectrum (400 MHz) in *$CDCl_3$* for the **PdC3** complex. with the assignments for the signals. 15
Figure S8. ^{1}H–^{1}H COSY for the PdC3 complex in *$CDCl_3$* with the main signals assigned and highlighted on the spectrum. 15
Figure S9. ^{13}C NMR spectrum in *$CDCl_3$* for the PdC3 complex with assigned signals and amplification of the region from 127 to 135 ppm........ 15
Figure S10. ^{13}C DEPT-135 NMR spectrum in *$CDCl_3$* for the **PdC3** complex. 16
Figure S11. ^{1}H-^{13}C-HSQC spectrum for the **PdC3** complex in *$CDCl_3$*. with the main signals assigned and emphasized on the spectrum........ 16
Figure S12. ^{31}P NMR spectra in *d_6*-DMSO for the Pd(II) complexes: line 1 (PdB1). line 2 (PdB3). line 3 (PdC1). line 4 (PdC2) and line 5 (PdC3)........ 17
Figure S13. ^{31}P{^{1}H} NMR spectra in *d_6*-DMSO for the **PdB3** complex at 0 (1). 24 (2) and 48 h (3)........ 17
Figure S14. FTIR vibrational spectrum in CsI for the PdB1. PdB3. PdC1. PdC2 and PdC3 complexes between 4400 - 250 cm^{-1}........ 18
Figure S15. Amplified mass spectrum for the PdB1 complex........ 19
Figure S16. Amplified mass spectrum for the PdB3 complex........ 19
Figure S17. Amplified mass spectrum for the PdC1 complex. 19
Figure S18. Amplified mass spectrum for the PdC2 complex. 20
Figure S19. Amplified mass spectrum for the PdC3 complex. 20
Figure S20. Structural representation of the **PdB1** complex obtained by X-ray diffraction. ellipsoids of 50% thermal probability........ 21
Figure S21. Structural representation of the PdB2 complex obtained by X-ray diffraction. ellipsoids of 50% thermal probability........ 21
Figure S22. Structural representation of the **PdB3** complex obtained by X-ray diffraction. ellipsoids of 50% thermal probability........ 22
Figure S23. Structural representation of the **PdC1** complex obtained by X-ray diffraction. ellipsoids of 50% thermal probability........ 22
Figure S24. Structural representation of the **PdC3** complex obtained by X-ray diffraction. ellipsoids of 50% thermal probability........ 23
Figure S25. Structural representation of the PdC3-DMSO complex obtained by X-ray diffraction. ellipsoids of 50% thermal probability. 23
Figure S26. Dose-response IC_{50} curve of compound PdB1 against the MDA-MB-231 cell line. The x-axis represents the logarithm of the concentration (μmol·L^{-1}). Curves A (blue circles), B (red squares), and C (green triangles) represent three independent

experimental replicates performed in triplicate. Data are expressed as mean ± standard deviation. 27
Figure S27. Dose-response IC_{50} curve of compound PdB2 against the MDA-MB-231 cell line. The x-axis represents the logarithm of the concentration (μmol·L^{-1}). Curves A (blue circles), B (red squares), and C (green triangles) represent three independent experimental replicates performed in triplicate. Data are expressed as mean ± standard deviation. 27
Figure S28. Dose-response IC_{50} curve of compound PdB3 against the MDA-MB-231 cell line. The x-axis represents the logarithm of the concentration (μmol·L^{-1}). Curves A (blue circles), B (red squares), and C (green triangles) represent three independent experimental replicates performed in triplicate. Data are expressed as mean ± standard deviation. 28
Figure S29. Dose-response IC_{50} curve of compound PdC1 against the MDA-MB-231 cell line. The x-axis represents the logarithm of the concentration (μmol·L^{-1}). Curves A (blue circles), B (red squares), and C (green triangles) represent three independent experimental replicates performed in triplicate. Data are expressed as mean ± standard deviation. 28
Figure S30. Dose-response IC_{50} curve of compound PdC2 against the MDA-MB-231 cell line. The x-axis represents the logarithm of the concentration (μmol·L^{-1}). Curves A (blue circles), B (red squares), and C (green triangles) represent three independent experimental replicates performed in triplicate. Data are expressed as mean ± standard deviation. 29
Figure S31. Dose-response IC_{50} curve of compound CDDP (Cisplatin) against the MDA-MB-231 cell line. The x-axis represents the logarithm of the concentration (μmol·L^{-1}). Curves A (blue circles), B (red squares), and C (green triangles) represent three independent experimental replicates performed in triplicate. Data are expressed as mean ± standard deviation. 29
Figure S32. Dose-response IC_{50} curve of compound PdB1 against the MCF-7 cell line. The x-axis represents the logarithm of the concentration (μmol·L^{-1}). Curves A (blue circles), B (red squares), and C (green triangles) represent three independent experimental replicates performed in triplicate. Data are expressed as mean ± standard deviation... 30
Figure S33. Dose-response IC_{50} curve of compound PdB2 against the MCF-7 cell line. The x-axis represents the logarithm of the concentration (μmol·L^{-1}). Curves A (blue circles), B (red squares), and C (green triangles) represent three independent experimental replicates performed in triplicate. Data are expressed as mean ± standard deviation... 30
Figure S34. Dose-response IC_{50} curve of compound PdB3 against the MCF-7 cell line. The x-axis represents the logarithm of the concentration (μmol·L^{-1}). Curves A (blue circles), B (red squares), and C (green triangles) represent three independent experimental replicates performed in triplicate. Data are expressed as mean ± standard deviation... 31
Figure S35. Dose-response IC_{50} curve of compound PdC1 against the MCF-7 cell line. The x-axis represents the logarithm of the concentration (μmol·L^{-1}). Curves A (blue circles), B (red squares), and C (green triangles) represent three independent experimental replicates performed in triplicate. Data are expressed as mean ± standard deviation... 31

Figure S36. Dose-response IC_{50} curve of compound CDDP (Cisplatin) against the MCF-7 cell line. The x-axis represents the logarithm of the concentration (μmol·L^{-1}). Curves A (blue circles), B (red squares), and C (green triangles) represent three independent experimental replicates performed in triplicate. Data are expressed as mean ± standard deviation. 32
Figure S37. Dose-response IC_{50} curve of compound PdB1 against the SK-BR-3 cell line. The x-axis represents the logarithm of the concentration (μmol·L^{-1}). Curves A (blue circles), B (red squares), and C (green triangles) represent three independent experimental replicates performed in triplicate. Data are expressed as mean ± standard deviation. 32
Figure S38. Dose-response IC_{50} curve of compound PdB2 against the SK-BR-3 cell line. The x-axis represents the logarithm of the concentration (μmol·L^{-1}). Curves A (blue circles), B (red squares), and C (green triangles) represent three independent experimental replicates performed in triplicate. Data are expressed as mean ± standard deviation. 33
Figure S39. Dose-response IC_{50} curve of compound PdC1 against the SK-BR-3 cell line. The x-axis represents the logarithm of the concentration (μmol·L^{-1}). Curves A (blue circles), B (red squares), and C (green triangles) represent three independent experimental replicates performed in triplicate. Data are expressed as mean ± standard deviation. 33
Figure S40. Dose-response IC_{50} curve of compound PdB1 against the MCF10A cell line. The x-axis represents the logarithm of the concentration (μmol·L^{-1}). Curves A (blue circles), B (red squares), and C (green triangles) represent three independent experimental replicates performed in triplicate. Data are expressed as mean ± standard deviation. 34
Figure S41. Dose-response $IC5_0$ curve of compound PdC1 against the MCF10A cell line. The x-axis represents the logarithm of the concentration (μmol·L^{-1}). Curves A (blue circles), B (red squares), and C (green triangles) represent three independent experimental replicates performed in triplicate. Data are expressed as mean ± standard deviation. 34
Figure S42. Dose-response IC_{50} curve of compound PdB1 against the A2780 cell line. The x-axis represents the logarithm of the concentration (μmol·L^{-1}). Curves A (blue circles), B (red squares), and C (green triangles) represent three independent experimental replicates performed in triplicate. Data are expressed as mean ± standard deviation... 35
Figure S43. Dose-response IC_{50} curve of compound PdB2 against the A2780 cell line. The x-axis represents the logarithm of the concentration (μmol·L^{-1}). Curves A (blue circles), B (red squares), and C (green triangles) represent three independent experimental replicates performed in triplicate. Data are expressed as mean ± standard deviation... 35
Figure S44. Dose-response IC_{50} curve of compound PdB3 against the A2780 cell line. The x-axis represents the logarithm of the concentration (μmol·L^{-1}). Curves A (blue circles), B (red squares), and C (green triangles) represent three independent experimental replicates performed in triplicate. Data are expressed as mean ± standard deviation... 36

Figure S45. Dose-response IC_{50} curve of compound PdC1 against the A2780 cell line. The x-axis represents the logarithm of the concentration ($\mu mol \cdot L^{-1}$). Curves A (blue circles), B (red squares), and C (green triangles) represent three independent experimental replicates performed in triplicate. Data are expressed as mean ± standard deviation... 36
Figure S46. Dose-response IC_{50} curve of compound PdC2 against the A2780 cell line. The x-axis represents the logarithm of the concentration ($\mu mol \cdot L^{-1}$). Curves A (blue circles), B (red squares), and C (green triangles) represent three independent experimental replicates performed in triplicate. Data are expressed as mean ± standard deviation... 37
Figure S47. Dose-response IC_{50} curve of compound PdC3 against the A2780 cell line. The x-axis represents the logarithm of the concentration ($\mu mol \cdot L^{-1}$). Curves A (blue circles), B (red squares), and C (green triangles) represent three independent experimental replicates performed in triplicate. Data are expressed as mean ± standard deviation... 37
Figure S48. Dose-response IC_{50} curve of compound CDDP (Cisplatin) against the A2780 cell line. The x-axis represents the logarithm of the concentration ($\mu mol \cdot L^{-1}$). Curves A (blue circles), B (red squares), and C (green triangles) represent three independent experimental replicates performed in triplicate. Data are expressed as mean ± standard deviation. 38
Figure S49. Dose-response IC_{50} curve of compound PdB1 against the A2780cis cell line. The x-axis represents the logarithm of the concentration ($\mu mol \cdot L^{-1}$). Curves A (blue circles), B (red squares), and C (green triangles) represent three independent experimental replicates performed in triplicate. Data are expressed as mean ± standard deviation. 38
Figure S50. Dose-response IC_{50} curve of compound PdB2 against the A2780cis cell line. The x-axis represents the logarithm of the concentration ($\mu mol \cdot L^{-1}$). Curves A (blue circles), B (red squares), and C (green triangles) represent three independent experimental replicates performed in triplicate. Data are expressed as mean ± standard deviation. 39
Figure S51. Dose-response IC_{50} curve of compound PdB3 against the A2780cis cell line. The x-axis represents the logarithm of the concentration ($\mu mol \cdot L^{-1}$). Curves A (blue circles), B (red squares), and C (green triangles) represent three independent experimental replicates performed in triplicate. Data are expressed as mean ± standard deviation. 39
Figure S52. Dose-response IC_{50} curve of compound PdC1 against the A2780cis cell line. The x-axis represents the logarithm of the concentration ($\mu mol \cdot L^{-1}$). Curves A (blue circles), B (red squares), and C (green triangles) represent three independent experimental replicates performed in triplicate. Data are expressed as mean ± standard deviation. 40
Figure S53. Dose-response IC_{50} curve of compound PdC2 against the A2780cis cell line. The x-axis represents the logarithm of the concentration ($\mu mol \cdot L^{-1}$). Curves A (blue circles), B (red squares), and C (green triangles) represent three independent experimental replicates performed in triplicate. Data are expressed as mean ± standard deviation. 40

Figure S54. Dose-response IC_{50} curve of compound PdC3 against the A2780cis cell line. The x-axis represents the logarithm of the concentration (μmol·L^{-1}). Curves A (blue circles), B (red squares), and C (green triangles) represent three independent experimental replicates performed in triplicate. Data are expressed as mean ± standard deviation. 41
Figure S55. Dose-response IC_{50} curve of compound CDDP (Cisplatin) against the A2780cis cell line. The x-axis represents the logarithm of the concentration (μmol·L^{-1}). Curves A (blue circles), B (red squares), and C (green triangles) represent three independent experimental replicates performed in triplicate. Data are expressed as mean ± standard deviation. 41
Figure S56. Dose-response IC_{50} curve of compound PdB1 against the A549 cell line. The x-axis represents the logarithm of the concentration (μmol·L^{-1}). Curves A (blue circles), B (red squares), and C (green triangles) represent three independent experimental replicates performed in triplicate. Data are expressed as mean ± standard deviation... 42
Figure S57. Dose-response IC_{50} curve of compound PdB2 against the A549 cell line. The x-axis represents the logarithm of the concentration (μmol·L^{-1}). Curves A (blue circles), B (red squares), and C (green triangles) represent three independent experimental replicates performed in triplicate. Data are expressed as mean ± standard deviation... 42
Figure S58. Dose-response IC_{50} curve of compound PdB3 against the A549 cell line. The x-axis represents the logarithm of the concentration (μmol·L^{-1}). Curves A (blue circles), B (red squares), and C (green triangles) represent three independent experimental replicates performed in triplicate. Data are expressed as mean ± standard deviation... 43
Figure S59. Dose-response IC_{50} curve of compound CDDP (Cisplatin) against the A549 cell line. The x-axis represents the logarithm of the concentration (μmol·L^{-1}). Curves A (blue circles), B (red squares), and C (green triangles) represent three independent experimental replicates performed in triplicate. Data are expressed as mean ± standard deviation. 43
Figure S60. Dose-response IC_{50} curve of compound PdB1 against the MRC5 cell line. The x-axis represents the logarithm of the concentration (μmol·L^{-1}). Curves A (blue circles), B (red squares), and C (green triangles) represent three independent experimental replicates performed in triplicate. Data are expressed as mean ± standard deviation... 44
Figure S61. Dose-response IC_{50} curve of compound PdB2 against the MRC5 cell line. The x-axis represents the logarithm of the concentration (μmol·L^{-1}). Curves A (blue circles), B (red squares), and C (green triangles) represent three independent experimental replicates performed in triplicate. Data are expressed as mean ± standard deviation... 44
Figure S62. Dose-response IC_{50} curve of compound PdB3 against the MRC5 cell line. The x-axis represents the logarithm of the concentration (μmol·L^{-1}). Curves A (blue circles), B (red squares), and C (green triangles) represent three independent experimental replicates performed in triplicate. Data are expressed as mean ± standard deviation... 45
Figure S63. Dose-response IC_{50} curve of compound PdC1 against the MRC5 cell line. The x-axis represents the logarithm of the concentration (μmol·L^{-1}). Curves A (blue circles), B (red squares), and C (green triangles) represent three independent experimental replicates performed in triplicate. Data are expressed as mean ± standard deviation... 45

Figure S64. Dose-response IC_{50} curve of compound PdB1 against the A375 cell line. The x-axis represents the logarithm of the concentration (μmol·L^{-1}). Curves A (blue circles), B (red squares), and C (green triangles) represent three independent experimental replicates performed in triplicate. Data are expressed as mean ± standard deviation... 46
Figure S65. IC_{50} values (μmol·L^{-1}) of Pd(II) complexes (PdB1, PdB2, PdB3, PdC1, PdC2, PdC3) and cisplatin (CDDP) against breast cancer cell lines MDA-MB-231, MCF-7, SK-BR-3, and the non-tumorigenic epithelial cell line MCF10A. Data are presented as mean ± standard deviation. Statistical analysis was performed using one-way ANOVA, comparing the IC_{50} values of each Pd(II) complex to the respective IC_{50} value of cisplatin (CDDP) within each cell line. Significance levels are indicated as ****$p < 0.0001$. Arrows indicate: ↑ $IC_{50} > 50$ μmol·L^{-1}; ↓ $IC_{50} < 2$ μmol·L^{-1}. 47
Figure S66. IC_{50} values (μmol·L^{-1}) of Pd(II) complexes PdC1, PdC2, and PdC3 compared within each cell line. CDDP was included as a reference compound. Data are presented as mean ± standard deviation. Statistical analysis was performed using one-way ANOVA, comparing IC_{50} values among PdC1, PdC2, and PdC3 within each cell line. Significance levels are indicated as ****$p < 0.0001$ and ns (not significant). The upward arrow (↑) indicates $IC_{50} > 50$ μmol·L^{-1}........ 47
Figure S67. (A) IC_{50} values (μmol·L^{-1}) of Pd(II) complexes PdB1, PdB2, PdB3, PdC1, PdC2, and PdC3 against A2780 (cisplatin-sensitive) and A2780cis (cisplatin-resistant) ovarian cancer cell lines. CDDP was included as a reference compound. The dashed line at 4.5 μ........ 48
Figure S68. (A) IC_{50} values (μmol·L^{-1}) of Pd(II) complexes PdB1, PdB2, PdB3, PdC1, PdC2, and PdC3 in ovarian cancer cell lines sensitive (A2780, cyan bars) and resistant (A2780cis, orange bars) to cisplatin. CDDP was included as a reference compound. The dashed line at 1 μmol·L^{-1} indicates the threshold adopted for better visualization in panel B. (B) Magnification of panel A highlighting compounds with IC_{50} values below 1 μmol·L^{-1}. Data are presented as mean ± standard deviation. Statistical analysis was performed using two-way ANOVA followed by Sidak's multiple comparisons test. Significance levels are indicated as ****$p < 0.0001$ and ns (not significant)........ 49
Figure S69. IC_{50} values (μmol·L^{-1}) of Pd(II) complexes PdB1, PdB2, PdB3, PdC1, PdC2, PdC3, and cisplatin (CDDP) against A549 (lung cancer), MRC-5 (normal lung fibroblast), and A375 (melanoma) cell lines. CDDP was included as a reference compound. The comparisons were performed between each Pd(II) complex and cisplatin within the same cell line. Data are presented as mean ± standard deviation. Statistical analysis was performed using two-way ANOVA followed by Tukey's multiple comparisons test. Significance levels are indicated as ****$p < 0.0001$ and ns (not significant)........ 50
Figure S70. Microscopy images of the morphological evaluation assay for the **PdC1** complex at different concentrations, in the A2780cis cell line and different times........ 51
Figure S71. Microscopy images of the cell migration assay at times 0 h and 30 h after the addition of the compound PdC1 at different concentrations, in the A2780cis cell line. 52

List of Table

Table S1. Values of the main distances and bond angles of the structures obtained. 24
Table S2. X-Ray crystallographic data collection and refinement parameters for complexes PdB1 and PdB3. 25
Table S3. X-Ray crystallographic data collection and refinement parameters for complexes PdC1, PdC3 and PdC3-DMSO. 26

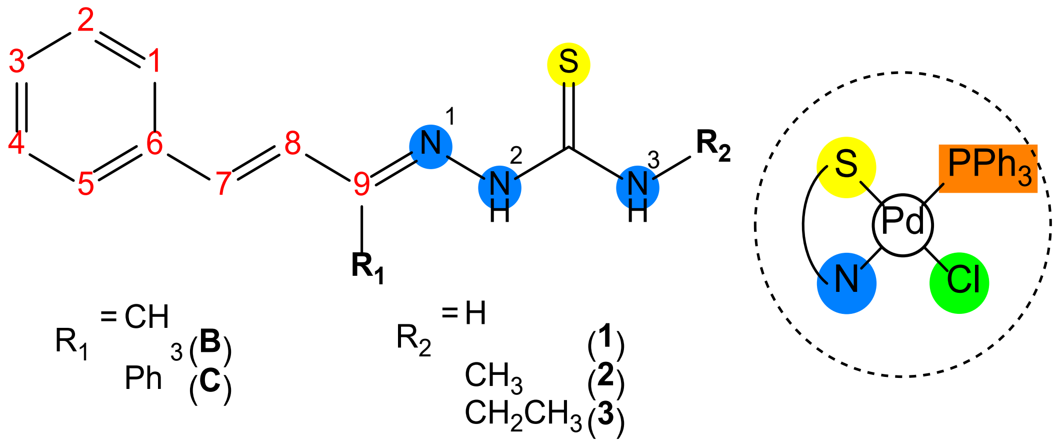

Figure S1. Structural proposal for Pd(II) complexes.

NMR spectra

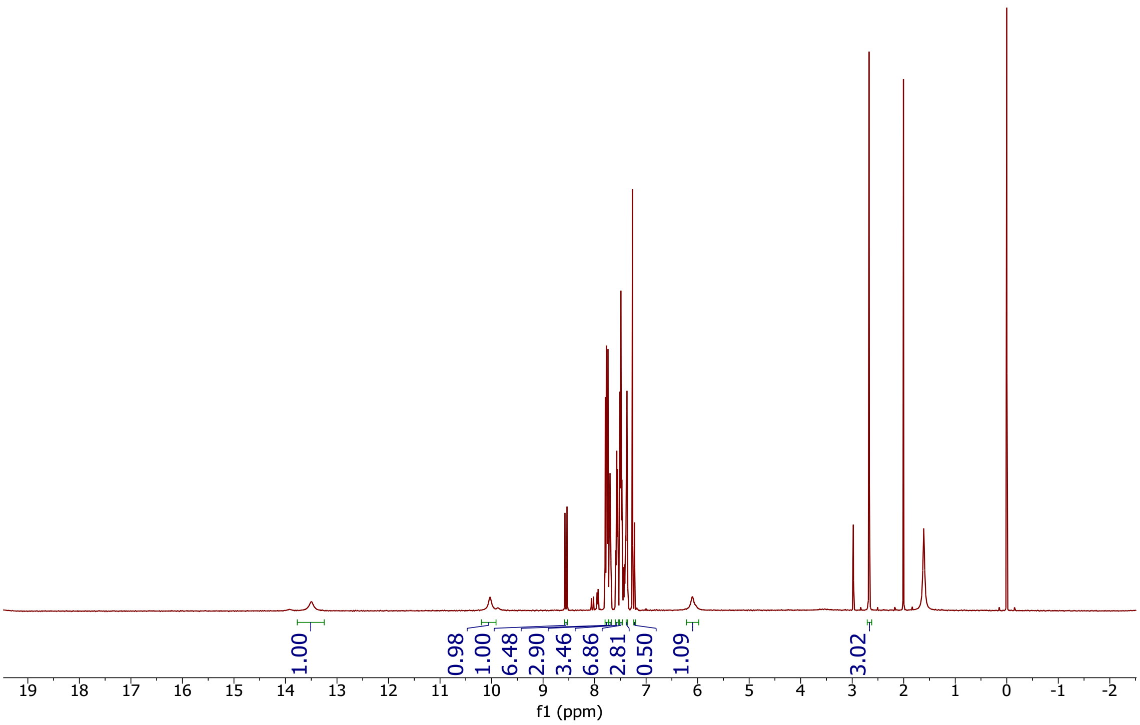

Figure S2. ^{1}H NMR spectrum (400 MHz) in *$CDCl_3$* for the **PdB1** complex.

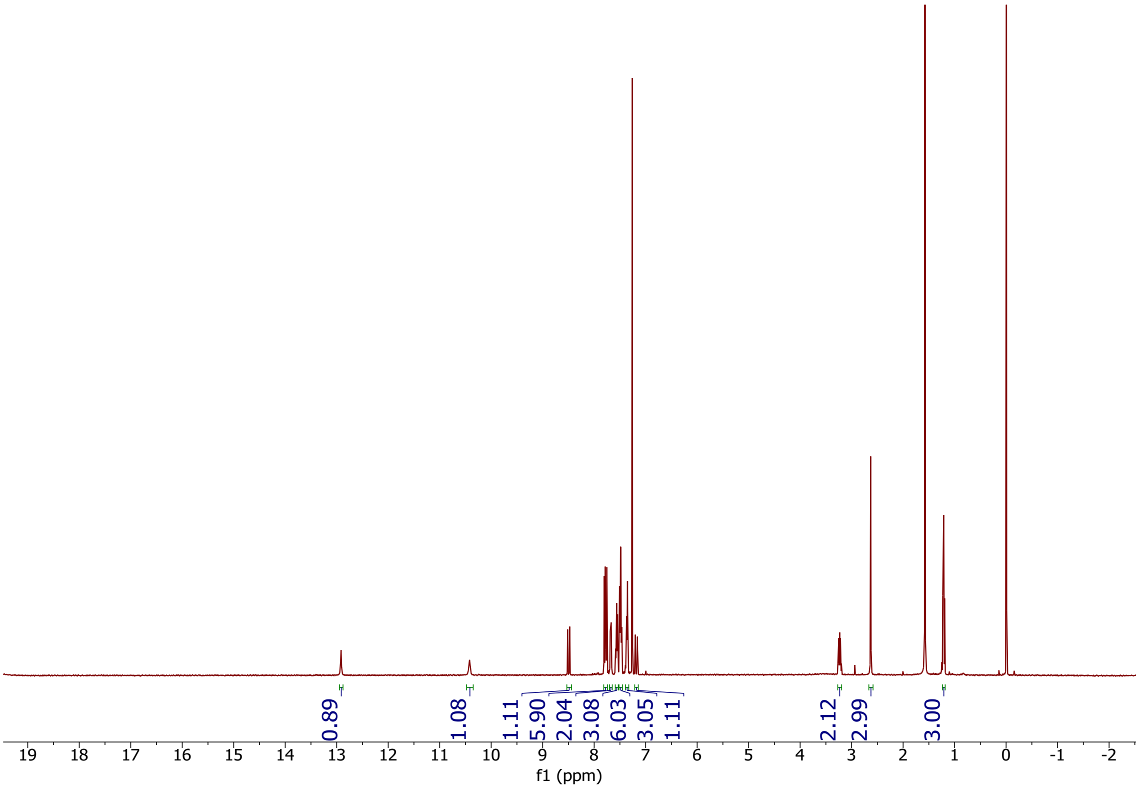

Figure S3. ^{1}H NMR spectrum (400 MHz) in *$CDCl_3$* for the **PdB3** complex.

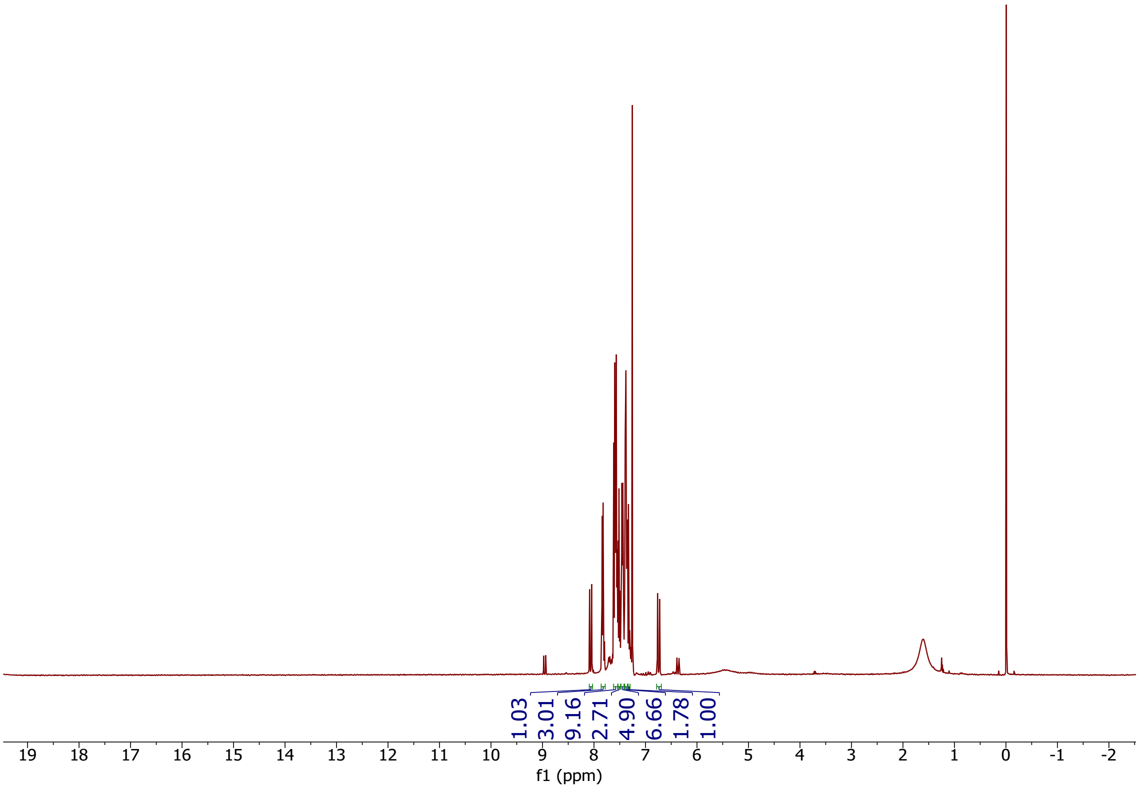

Figure S4. ^{1}H NMR spectrum (400 MHz) in *$CDCl_3$* for the **PdC1** complex.

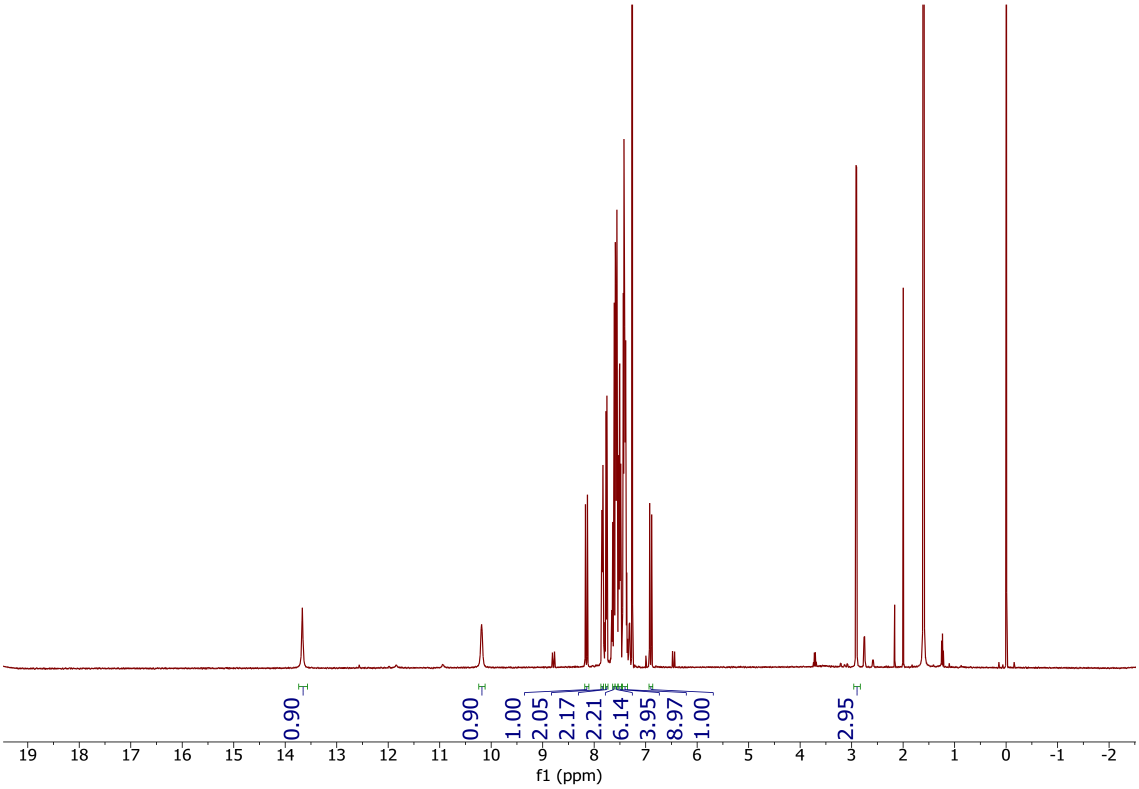

Figure S5. ^{1}H NMR spectrum (400 MHz) in *$CDCl_3$* for the **PdC2** complex.

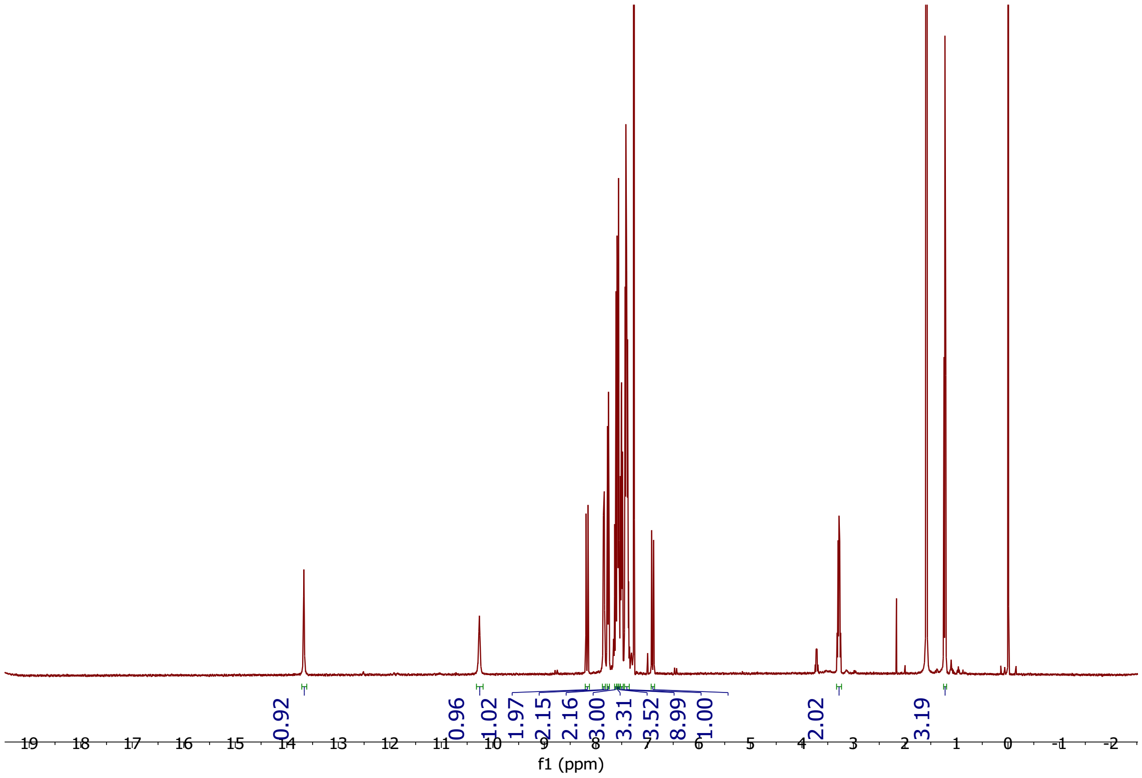

Figure S6. ^{1}H NMR spectrum (400 MHz) in *$CDCl_3$* for the **PdC3** complex.

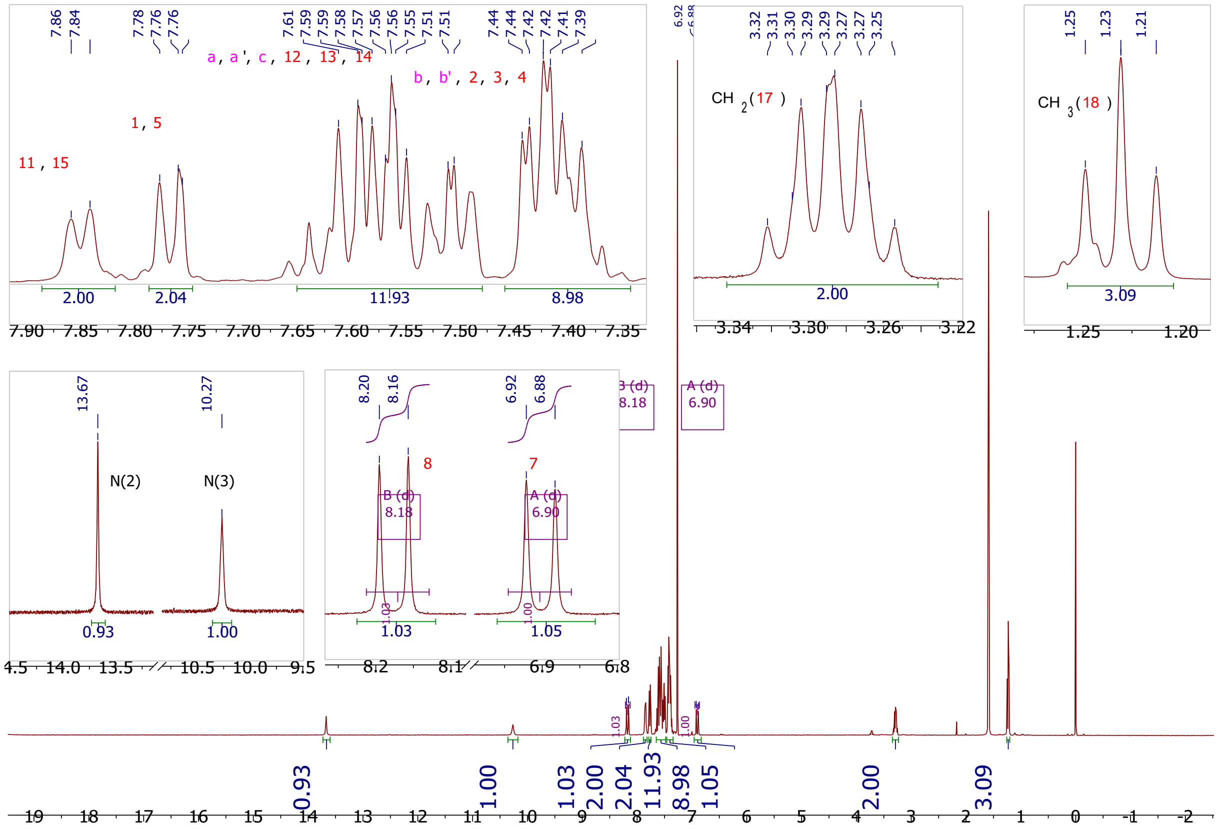

Figure S7. ^{1}H NMR spectrum (400 MHz) in *$CDCl_3$* for the **PdC3** complex. with the assignments for the signals.

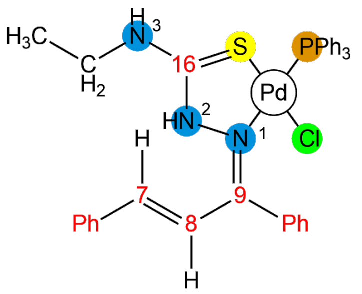

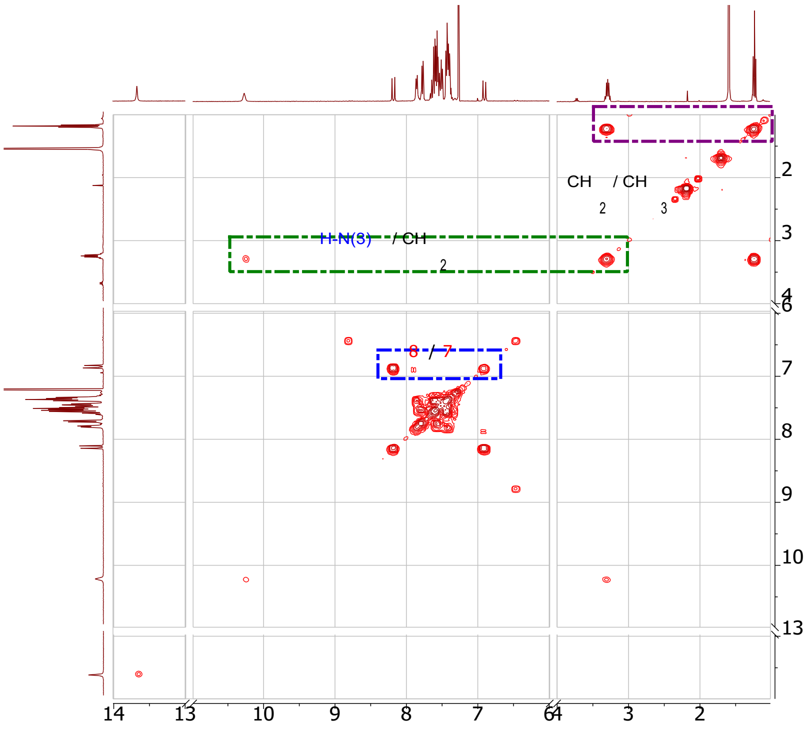

Figure S8. ^{1}H–^{1}H COSY for the **PdC3** complex in *$CDCl_3$* with the main signals assigned and highlighted on the spectrum.

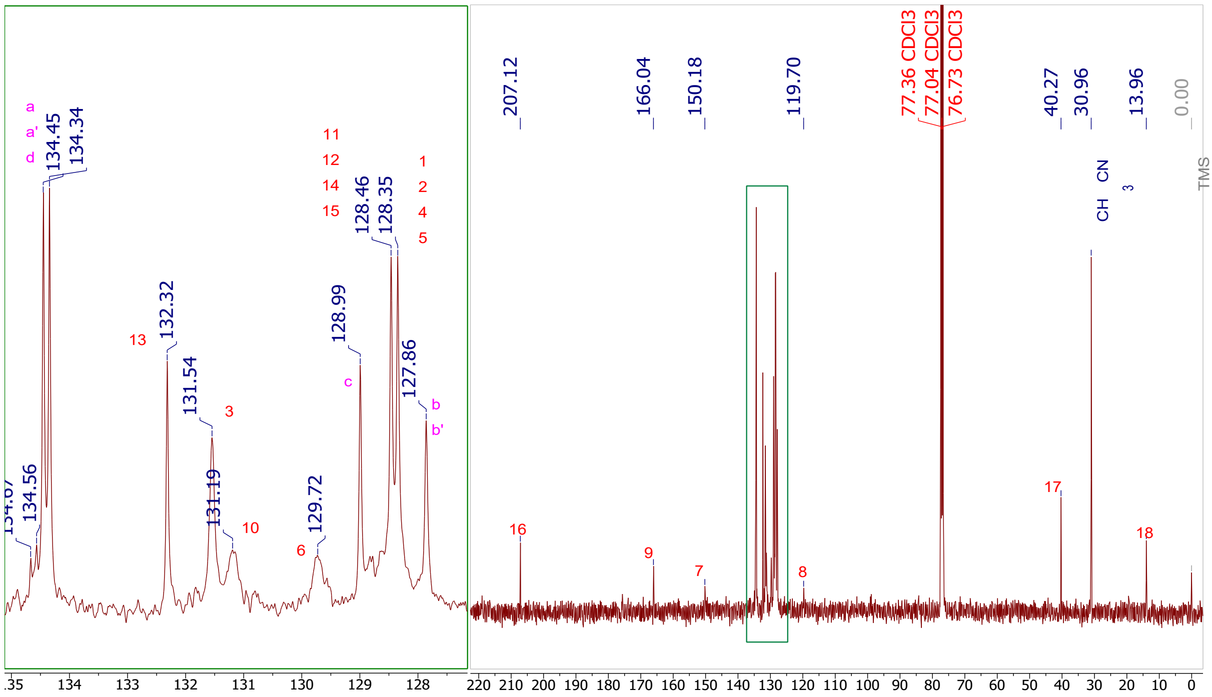

Figure S9. ^{13}C NMR spectrum in *$CDCl_3$* for the **PdC3** complex with assigned signals and amplification of the region from 127 to 135 ppm.

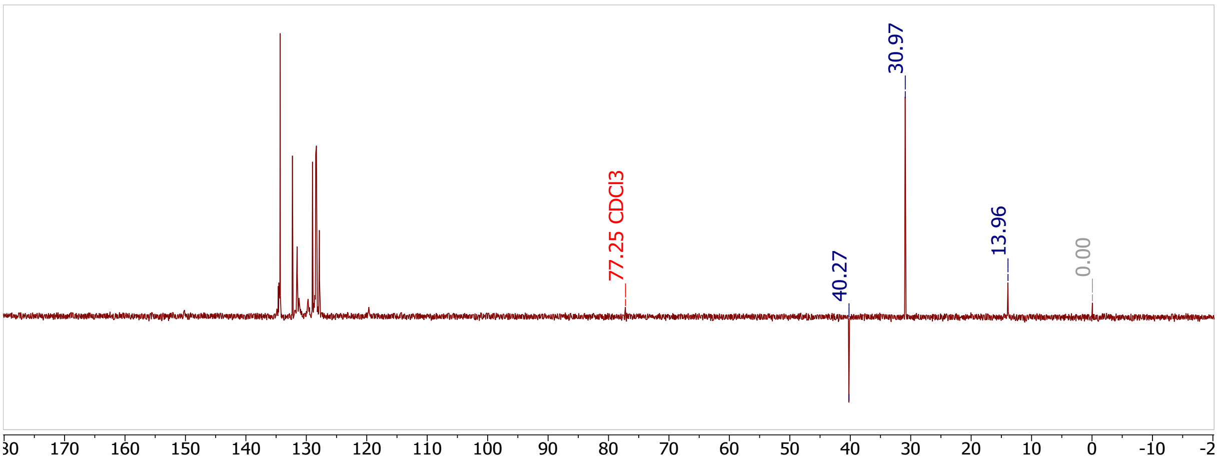

Figure S10. ^{13}C DEPT-135 NMR spectrum in *$CDCl_3$* for the **PdC3** complex.

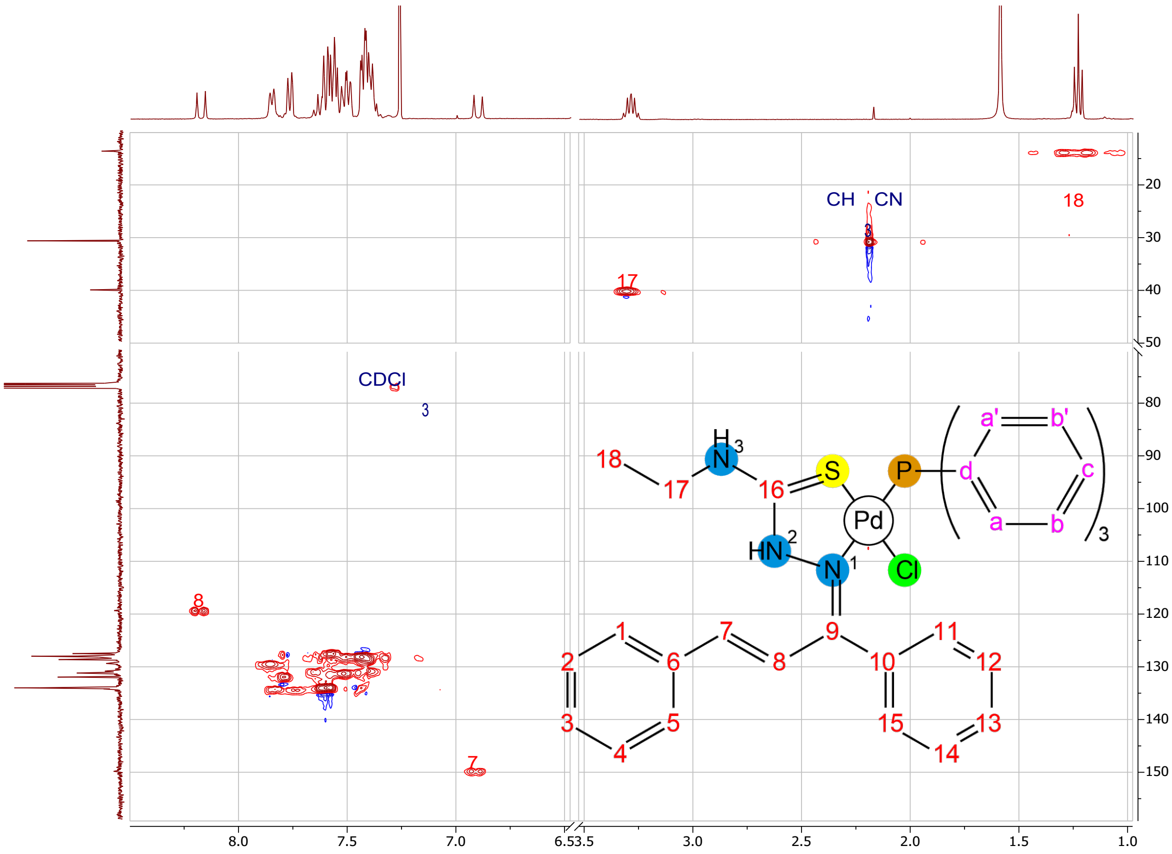

Figure S11. ^{1}H-^{13}C-HSQC spectrum for the **PdC3** complex in *$CDCl_3$*. with the main signals assigned and emphasized on the spectrum.

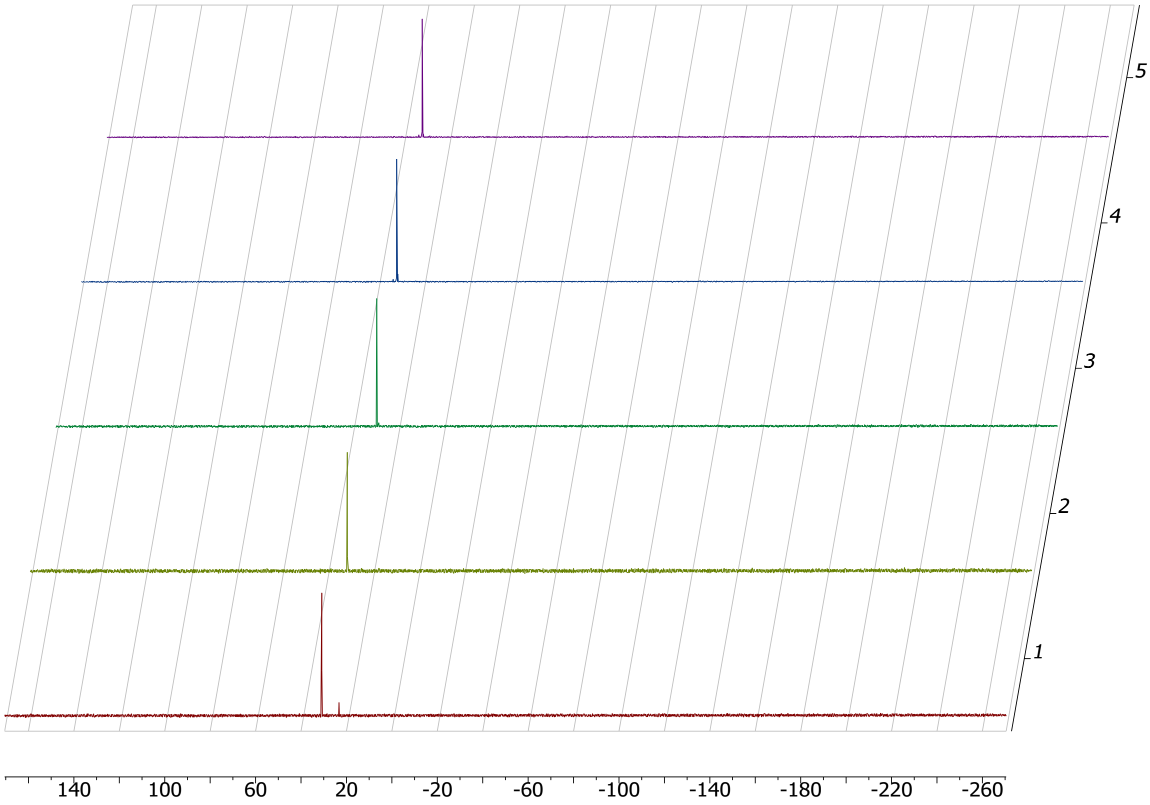

Figure S12. ^{31}P NMR spectra in d_6-DMSO for the Pd(II) complexes: line 1 (**PdB1**). line 2 (**PdB3**). line 3 (**PdC1**). line 4 (**PdC2**) and line 5 (**PdC3**).

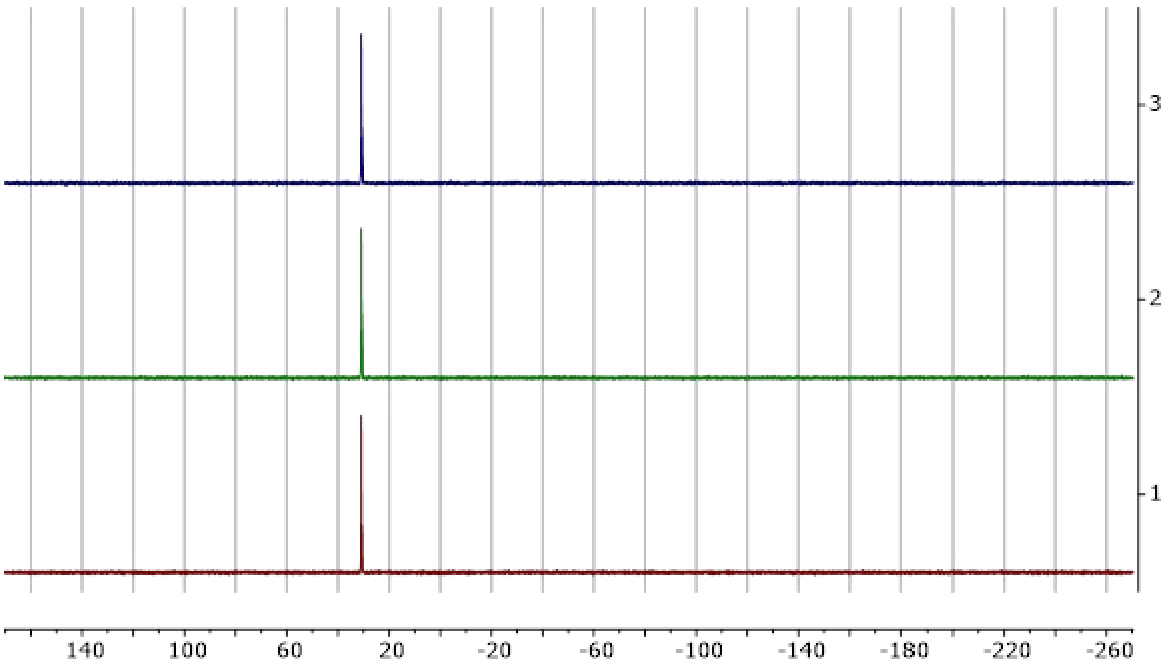

Figure S13. $^{31}P\{^1H\}$ NMR spectra in d_6-DMSO for the **PdB3** complex at 0 (1). 24 (2) and 48 h (3).

IR data

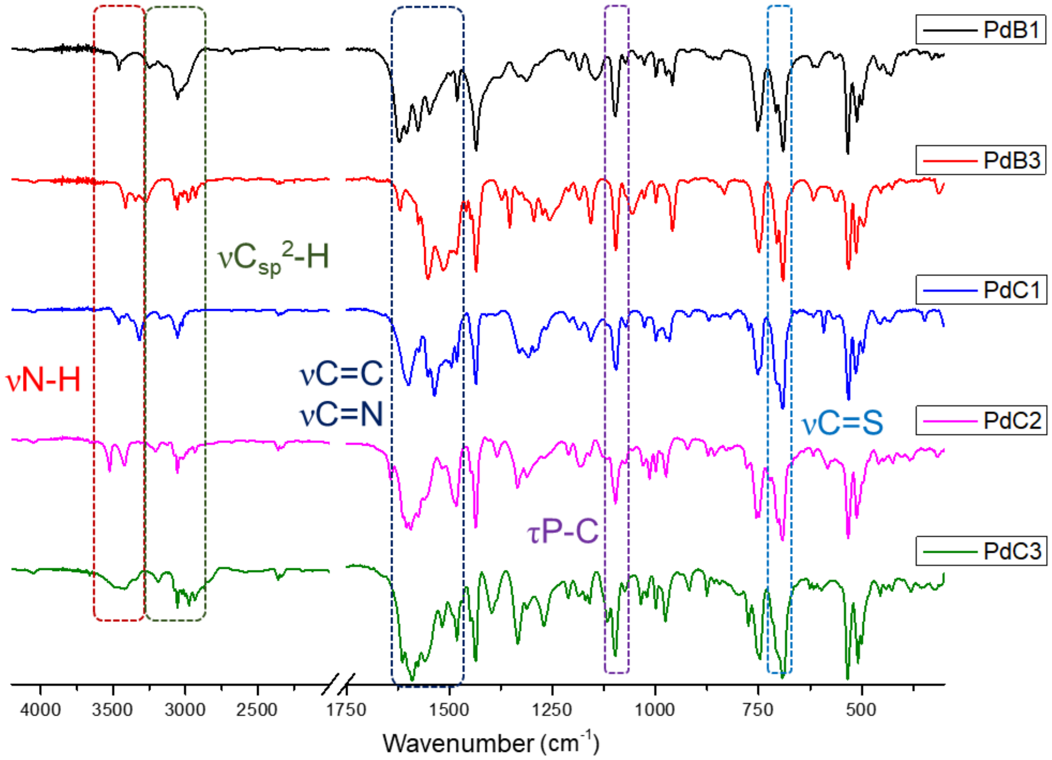

Figure S14. FTIR vibrational spectrum in CsI for the **PdB1**. **PdB3**. **PdC1**. **PdC2** and **PdC3** complexes between 4400 - 250 cm^{-1}.

Mass spectrometry

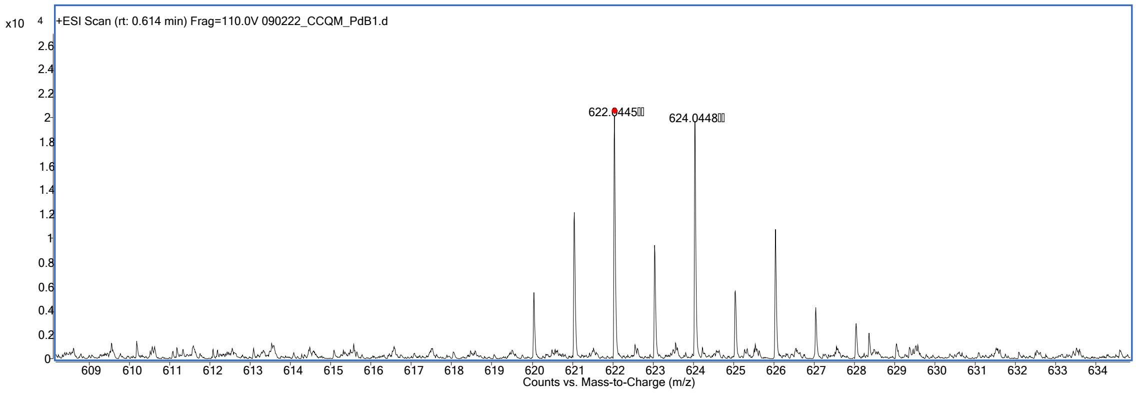

Figure S15. Amplified mass spectrum for the **PdB1** complex.

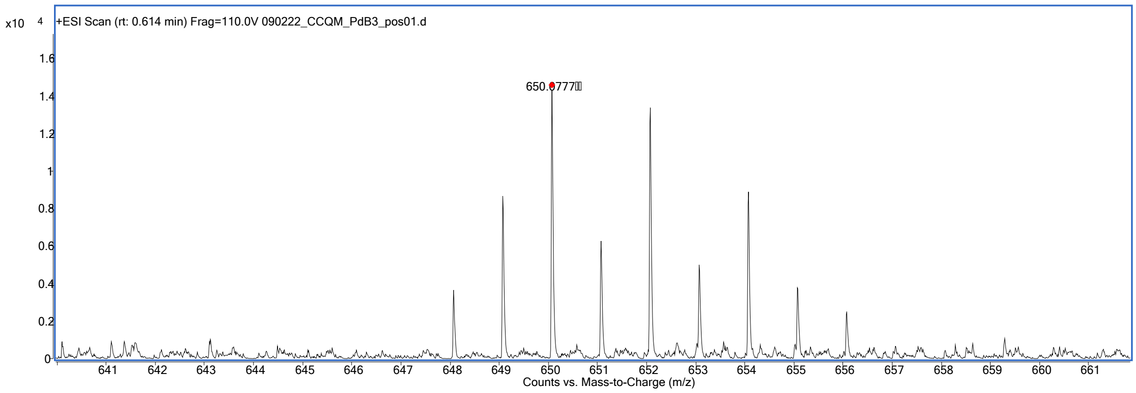

Figure S16. Amplified mass spectrum for the **PdB3** complex.

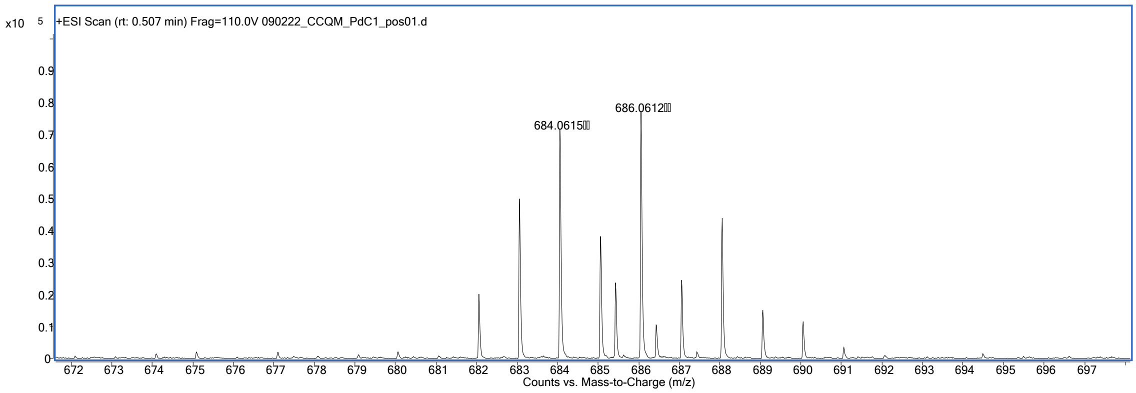

Figure S17. Amplified mass spectrum for the **PdC1** complex.

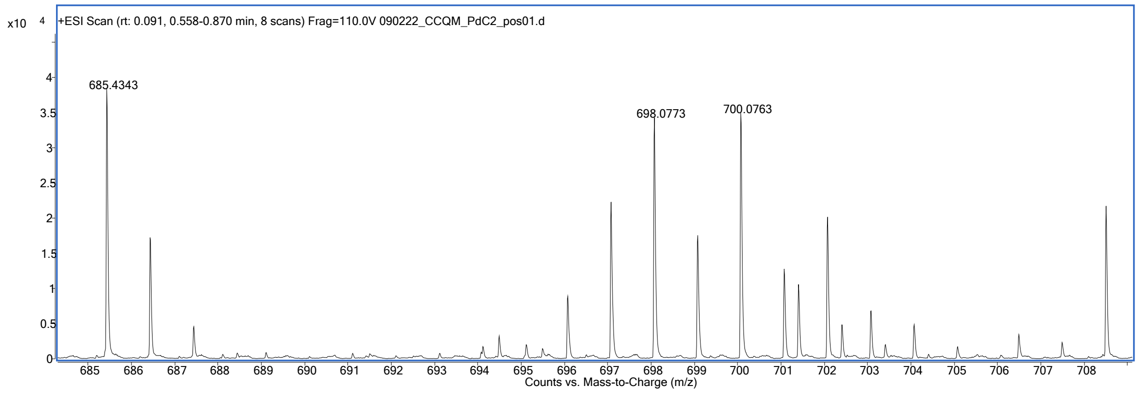

Figure S18. Amplified mass spectrum for the **PdC2** complex.

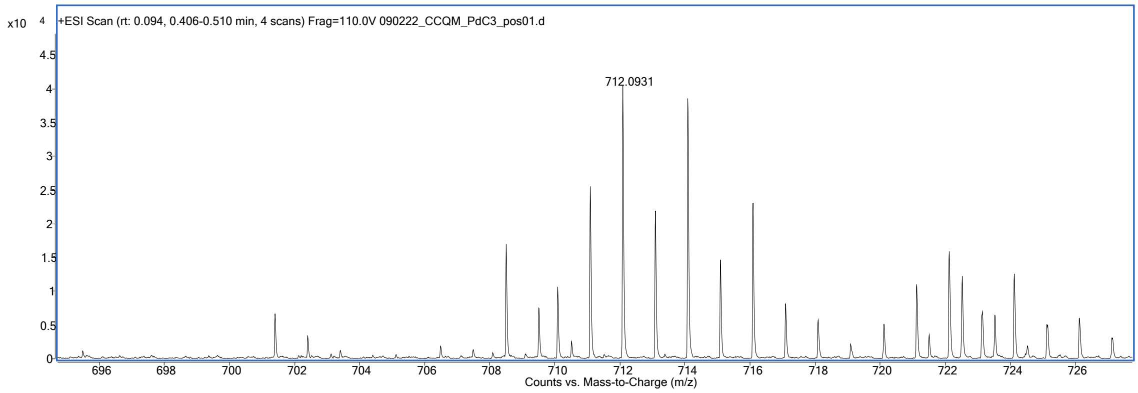

Figure S19. Amplified mass spectrum for the **PdC3** complex.

Crystallographic data and details of the refinement of the compounds

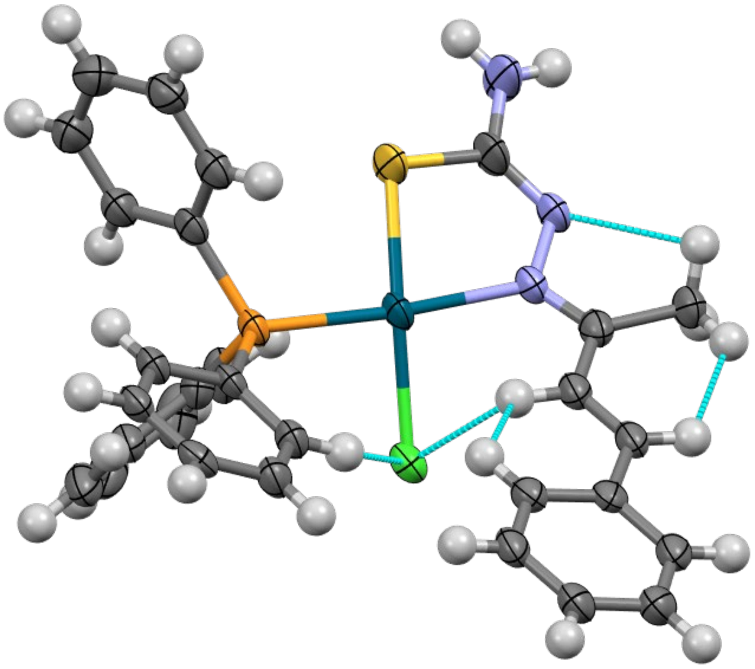

Figure S20. Structural representation of the **PdB1** complex obtained by X-ray diffraction. ellipsoids of 50% thermal probability.

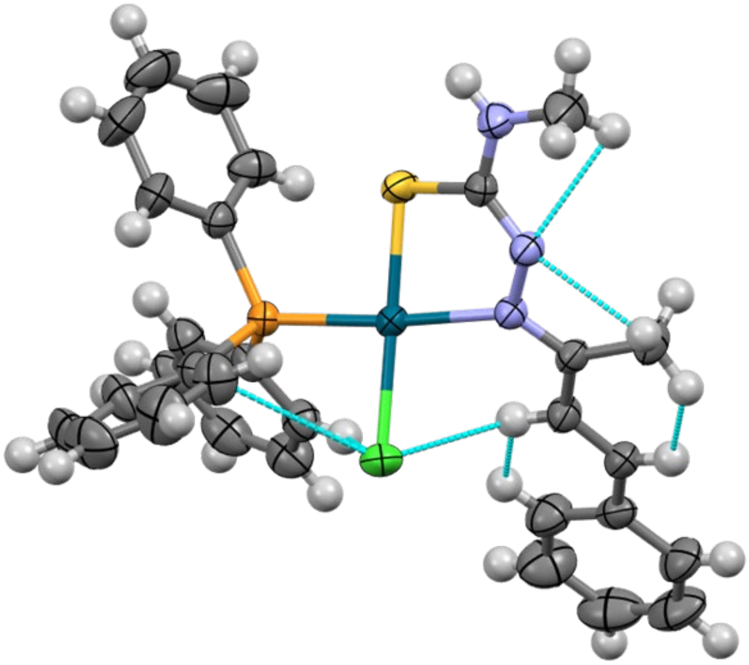

Figure S21. Structural representation of the **PdB2** complex obtained by X-ray diffraction. ellipsoids of 50% thermal probability.

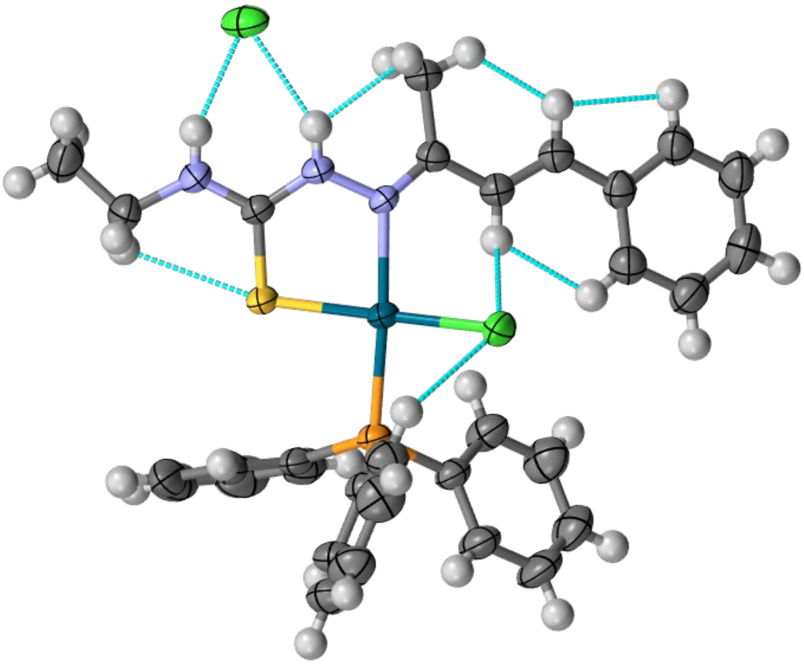

Figure S22. Structural representation of the **PdB3** complex obtained by X-ray diffraction. ellipsoids of 50% thermal probability.

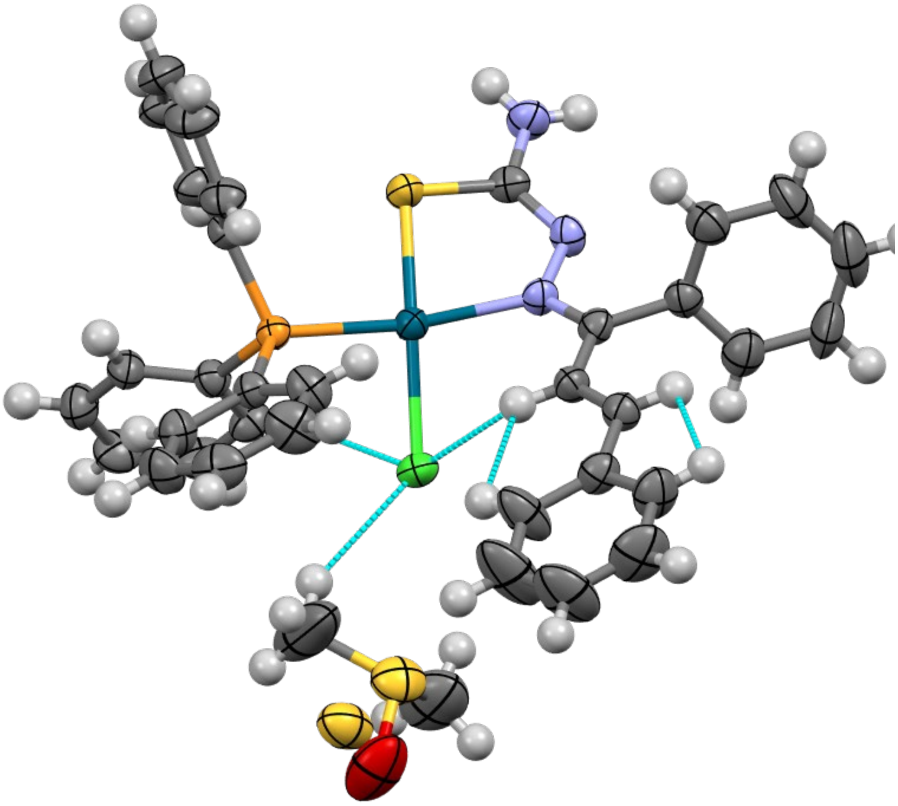

Figure S23. Structural representation of the **PdC1** complex obtained by X-ray diffraction. ellipsoids of 50% thermal probability.

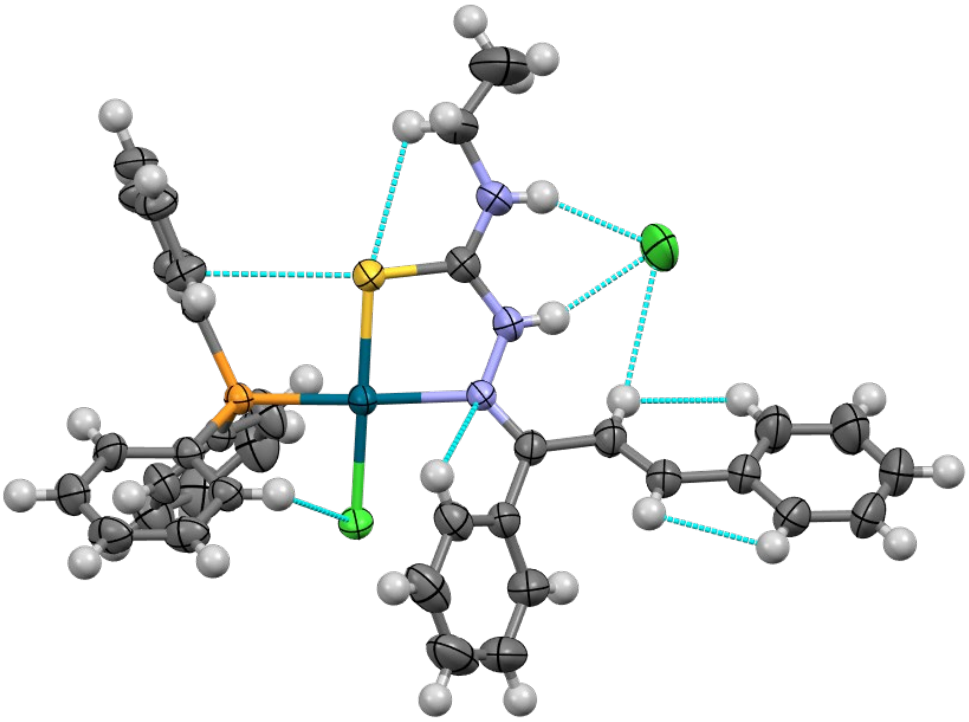

Figure S24. Structural representation of the **PdC3** complex obtained by X-ray diffraction. ellipsoids of 50% thermal probability.

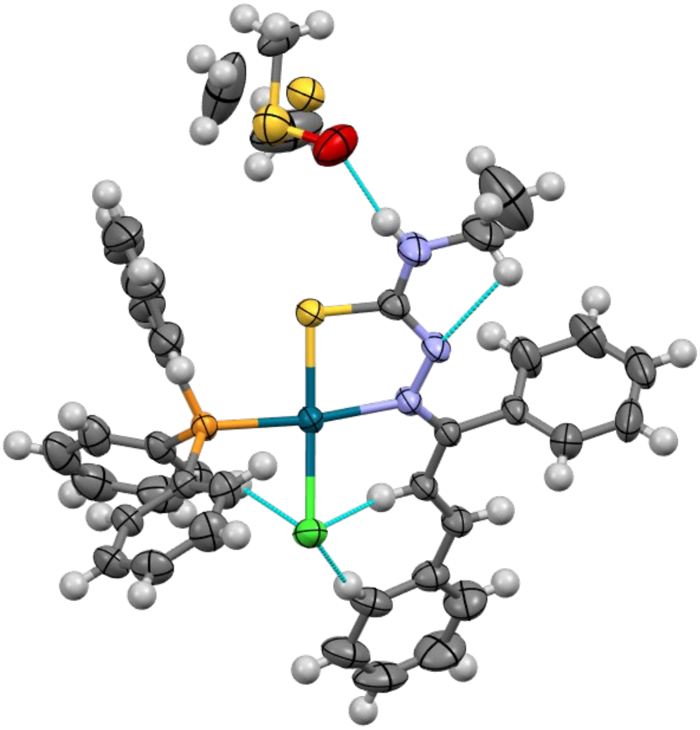

Figure S25. Structural representation of the **PdC3-DMSO** complex obtained by X-ray diffraction. ellipsoids of 50% thermal probability.

Table S1. Values of the main distances and bond angles of the structures obtained.

Complexes	PdB1*	PdB2	PdB3	PdC1*	PdC3-Cl	PdC3-DMSO
Pd–S	2.261	2.251	2.267	2.252	2.279	2.243
Pd–N(1)	2.095	2.097	2.131	2.118	2.146	2.122
Pd–P	2.252	2.255	2.254	2.250	2.247	2.245
Pd–Cl	2.359	2.338	2.327	2.355	2.317	2.353
S–C(10)	1.772	1.755	1.724	1.759	1.714	1.745
N(1)-C(9)	1.302	1.296	1.293	1.292	1.299	1.308
N(2)–C(10)	1.304	1.286	1.336	1.312	1.349	1.303
S-Pd-Cl	165.78	169.47	166.43	164.94	174.66	170.92
N-Pd-P	173.71	175.03	170.47	167.81	176.68	172.92
S-Pd-N	81.44	81.14	82.74	81.53	83.32	81.77
P-Pd-Cl	89.13	88.69	89.61	88.58	84.24	86.33
S-Pd-P	94.10	93.93	92.24	92.93	94.69	91.68
N-Pd-Cl	96.26	96.28	97.17	99.61	97.49	100.66

* Crystallized in DMSO.

Table S2. X-Ray crystallographic data collection and refinement parameters for complexes **PdB1** and **PdB3.**

**Compound**	**PdB1**	**PdB2**	**PdB3**
Empirical formula	$C_{29}H_{27}ClN_3PPdS$	2($C_{30}H_{29}ClN_3PPdS$)	$C_{31}H_{32}Cl_2N_3PPdS$
Formula weight	622.41	1272.88	686.92
Temperature/K	100.0	293	293(2)
Crystal system	monoclinic	triclinic	triclinic
Space group	$P2_1/c$	P-1	P-1
a/Å	17.2041(5)	11.5049(3)	8.0497(3)
b/Å	12.5527(3)	16.2048(4)	12.0856(4)
c/Å	15.4719(4)	17.4711(4)	16.5953(5)
α/°	90	112.378(2)	93.273(3)
β/°	93.327(2)	92.328(2)	98.749(3)
γ/°	90	92.937(2)	101.680(3)
Volume/Å^3	3335.65(15)	3001.58(13)	1556.20(9)
Z	4	2	2
ρ_{calc}g/cm^3	1.239	1.408	1.466
μ/mm^{-1}	0.766	0.853	0.912
F(000)	1264.0	1296.0	700.0
Crystal size/mm^3	0.197 × 0.123 × 0.066		0.409 × 0.364 × 0.28
Radiation	Mo Kα (λ = 0.71073)		Mo Kα (λ = 0.71073)
2Θ range for data collection/°	4.182 to 51.356		5.242 to 51.5
Index ranges	$-20 \leq h \leq 20$. $-15 \leq k \leq 13$. $-18 \leq l \leq 18$		$-9 \leq h \leq 9$. $-14 \leq k \leq 14$. $-20 \leq l \leq 20$
Reflections collected	34441	11774	30876
Independent reflections	6330 [R_{int} = 0.0342. R_{sigma} = 0.0239]		5947 [R_{int} = 0.0239. R_{sigma} = 0.0158]
Data/restraints/parameters	6330/0/326		5947/0/354
Goodness-of-fit on F^2	1.045		1.167
Final R indexes [I>=2σ (I)]	R_1 = 0.0347, wR_2 = 0.0790	R_1 = 0.0313, wR_2 = 0.0800	R_1 = 0.0264, wR_2 = 0.0562
Final R indexes [all data]	R_1 = 0.0406, wR_2 = 0.0813		R_1 = 0.0323, wR_2 = 0.0616
Largest diff. peak/hole / e Å^{-3}	0.82/-0.81		0.52/-0.31

Table S3. X-Ray crystallographic data collection and refinement parameters for complexes **PdC1**, **PdC3** and **PdC3-DMSO**.

**Compound**	**PdC1**	**PdC3**	**PdC3-DMSO**
Empirical formula	$C_{36}H_{35}ClN_3OPPdS_2$	$C_{36}H_{34}Cl_2N_3PPdS$	$C_{38}H_{39}ClN_3OPPdS_2$
Formula weight	762.61	748.99	790.66
Temperature/K	298(2)	293(2)	298(2)
Crystal system	monoclinic	triclinic	monoclinic
Space group	$P2_1$	P-1	$P2_1/c$
a/Å	8.9723(6)	10.2043(4)	17.7028(8)
b/Å	20.6518(13)	13.9510(6)	12.0178(6)
c/Å	10.5638(7)	14.1613(6)	17.8206(8)
α/°	90	117.596(4)	90
β/°	94.246(6)	93.622(3)	94.927(4)
γ/°	90	99.133(4)	90
Volume/Å^3	1952.0(2)	1742.34(14)	3777.3(3)
Z	2	2	4
ρ_{calc}g/cm^3	1.297	1.428	1.390
μ/mm^{-1}	0.721	0.821	0.748
F(000)	780.0	764.0	1624.0
Crystal size/mm^3	0.225 × 0.2 × 0.118	0.31 × 0.146 × 0.13	0.351 × 0.26 × 0.178
Radiation	Mo Kα (λ = 0.71073)	Mo Kα (λ = 0.71073)	Mo Kα (λ = 0.71073)
2Θ range for data collection/°	5.524 to 51.35	5.16 to 51.498	4.956 to 51.362
Index ranges	-10 ≤ h ≤ 7. -19 ≤ k ≤ 25. -12 ≤ l ≤ 11	-12 ≤ h ≤ 12. -17 ≤ k ≤ 17. -17 ≤ l ≤ 17	-21 ≤ h ≤ 21. -14 ≤ k ≤ 13. -21 ≤ l ≤ 21
Reflections collected	7258	27232	25390
Independent reflections	5839 [R_{int} = 0.0227. R_{sigma} = 0.0430]	6674 [R_{int} = 0.0345. R_{sigma} = 0.0293]	7161 [R_{int} = 0.0299. R_{sigma} = 0.0375]
Data/restraints/parameters	5839/41/417	6674/0/398	7161/61/386
Goodness-of-fit on F^2	1.057	1.085	1.103
Final R indexes [I>=2σ (I)]	R_1 = 0.0312. wR_2 = 0.0721	R_1 = 0.0299. wR_2 = 0.0715	R_1 = 0.0348. wR_2 = 0.0851
Final R indexes [all data]	R_1 = 0.0374. wR_2 = 0.0756	R_1 = 0.0408. wR_2 = 0.0807	R_1 = 0.0458. wR_2 = 0.0943
Largest diff. peak/hole / e Å^{-3}	0.43/-0.38	0.42/-0.36	0.55/-0.34

Dose-Response Curves for IC_{50} Determination

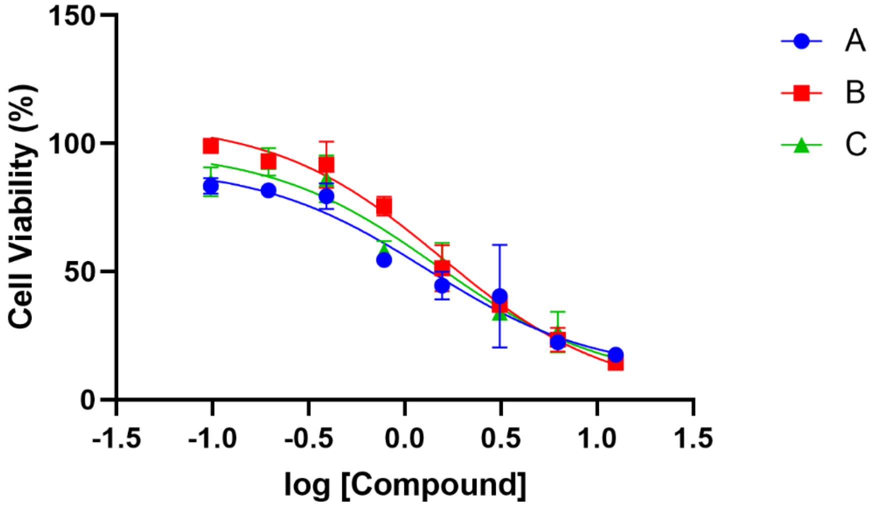

Figure S26. Dose-response IC_{50} curve of compound **PdB1** against the MDA-MB-231 cell line. The x-axis represents the logarithm of the concentration (μmol·L^{-1}). Curves A (blue circles), B (red squares), and C (green triangles) represent three independent experimental replicates performed in triplicate. Data are expressed as mean ± standard deviation.

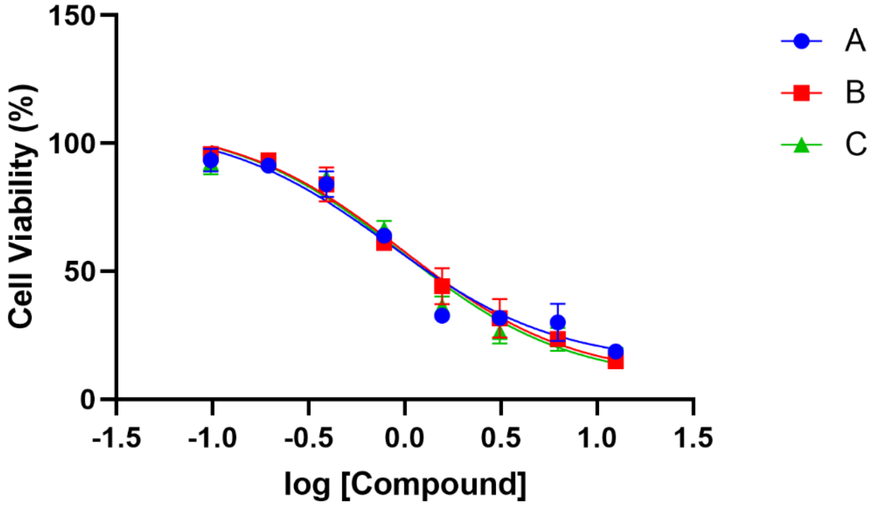

Figure S27. Dose-response IC_{50} curve of compound **PdB2** against the MDA-MB-231 cell line. The x-axis represents the logarithm of the concentration (μmol·L^{-1}). Curves A (blue circles), B (red squares), and C (green triangles) represent three independent experimental replicates performed in triplicate. Data are expressed as mean ± standard deviation.

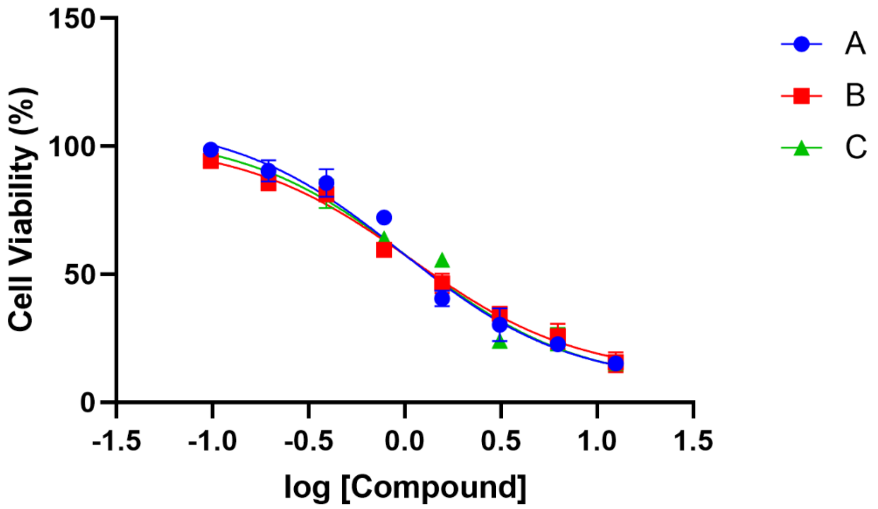

Figure S28. Dose-response IC_{50} curve of compound **PdB3** against the MDA-MB-231 cell line. The x-axis represents the logarithm of the concentration ($\mu mol \cdot L^{-1}$). Curves A (blue circles), B (red squares), and C (green triangles) represent three independent experimental replicates performed in triplicate. Data are expressed as mean ± standard deviation.

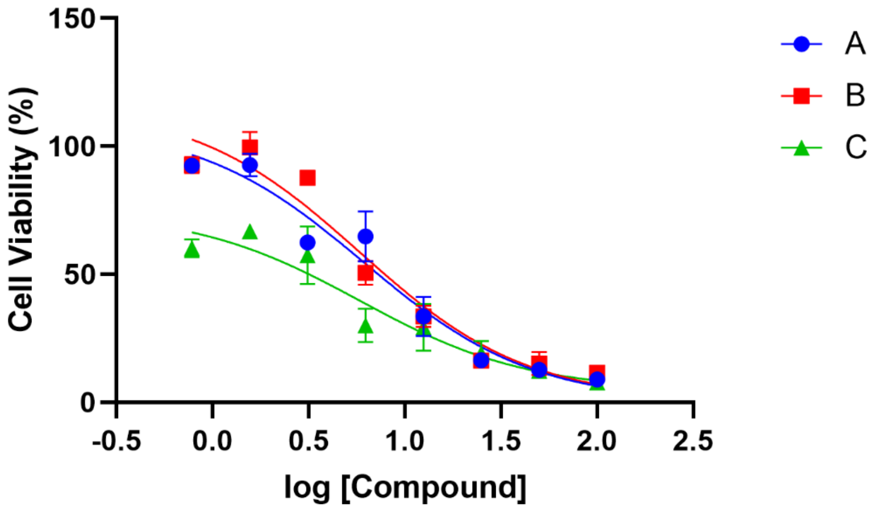

Figure S29. Dose-response IC_{50} curve of compound **PdC1** against the MDA-MB-231 cell line. The x-axis represents the logarithm of the concentration ($\mu mol \cdot L^{-1}$). Curves A (blue circles), B (red squares), and C (green triangles) represent three independent experimental replicates performed in triplicate. Data are expressed as mean ± standard deviation.

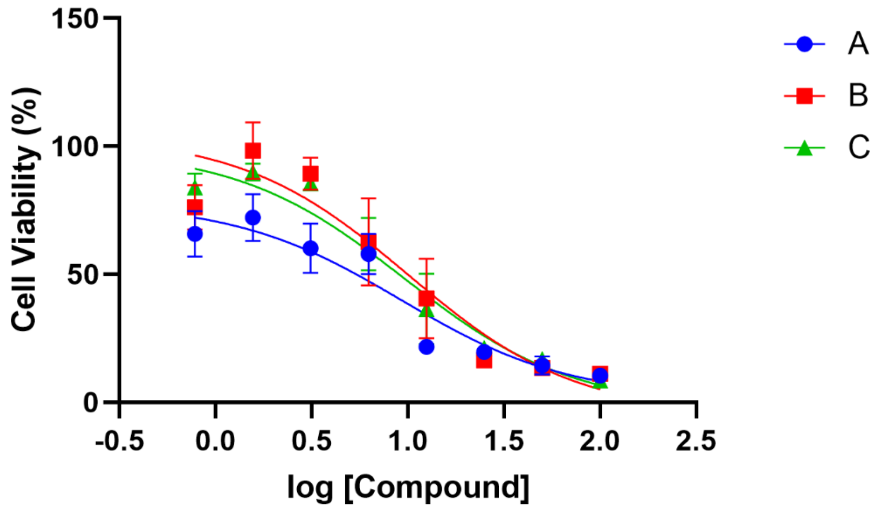

Figure S30. Dose-response IC_{50} curve of compound **PdC2** against the MDA-MB-231 cell line. The x-axis represents the logarithm of the concentration (μmol·L^{-1}). Curves A (blue circles), B (red squares), and C (green triangles) represent three independent experimental replicates performed in triplicate. Data are expressed as mean ± standard deviation.

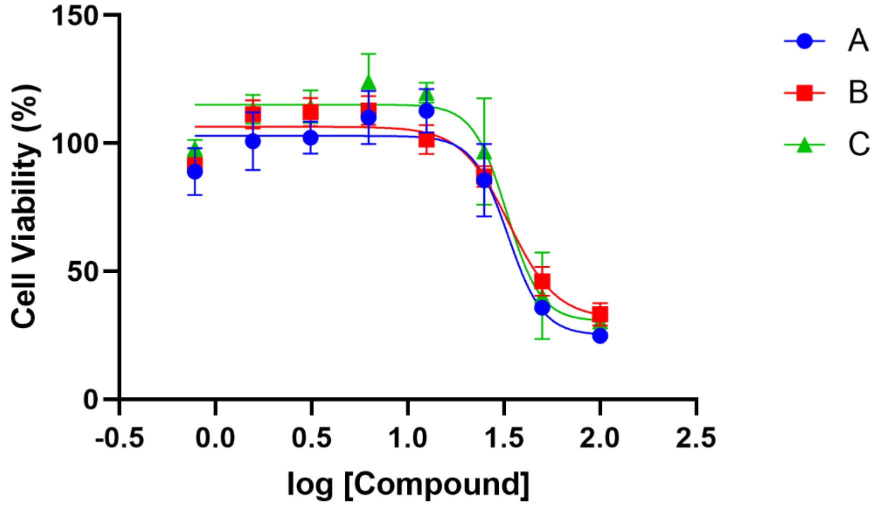

Figure S31. Dose-response IC_{50} curve of compound **CDDP** (Cisplatin) against the MDA-MB-231 cell line. The x-axis represents the logarithm of the concentration (μmol·L^{-1}). Curves A (blue circles), B (red squares), and C (green triangles) represent three independent experimental replicates performed in triplicate. Data are expressed as mean ± standard deviation.

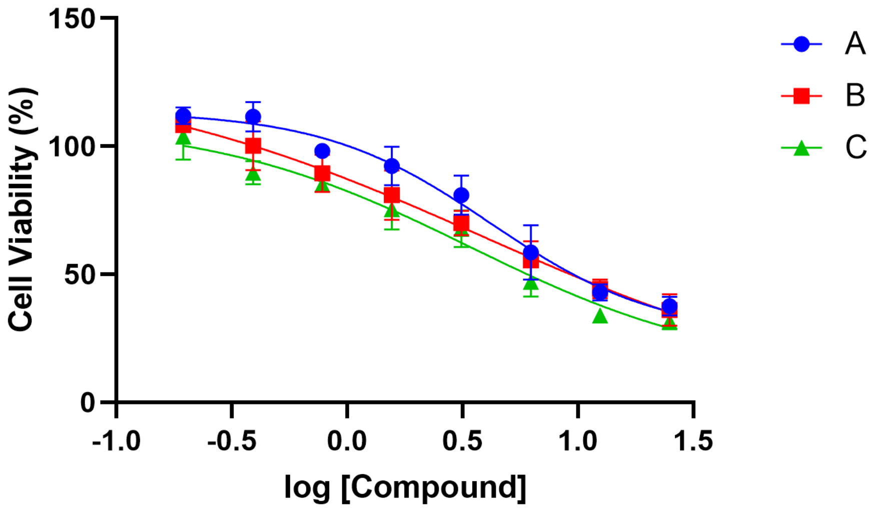

Figure S32. Dose-response IC_{50} curve of compound **PdB1** against the MCF-7 cell line. The x-axis represents the logarithm of the concentration (μmol·L^{-1}). Curves A (blue circles), B (red squares), and C (green triangles) represent three independent experimental replicates performed in triplicate. Data are expressed as mean ± standard deviation.

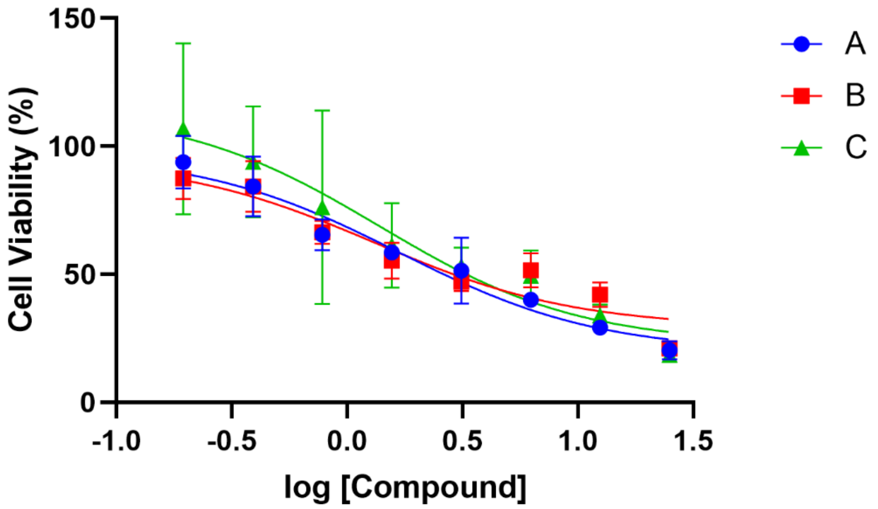

Figure S33. Dose-response IC_{50} curve of compound **PdB2** against the MCF-7 cell line. The x-axis represents the logarithm of the concentration (μmol·L^{-1}). Curves A (blue circles), B (red squares), and C (green triangles) represent three independent experimental replicates performed in triplicate. Data are expressed as mean ± standard deviation.

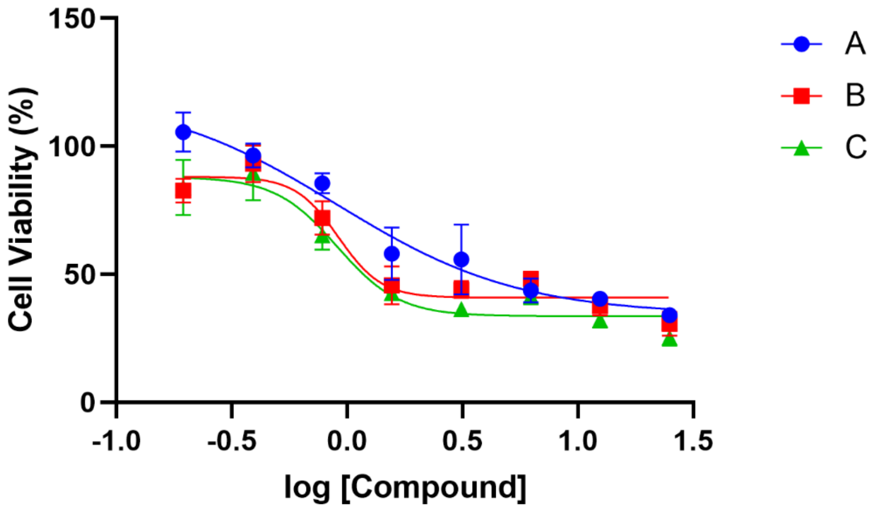

Figure S34. Dose-response IC_{50} curve of compound **PdB3** against the MCF-7 cell line. The x-axis represents the logarithm of the concentration ($\mu mol \cdot L^{-1}$). Curves A (blue circles), B (red squares), and C (green triangles) represent three independent experimental replicates performed in triplicate. Data are expressed as mean ± standard deviation.

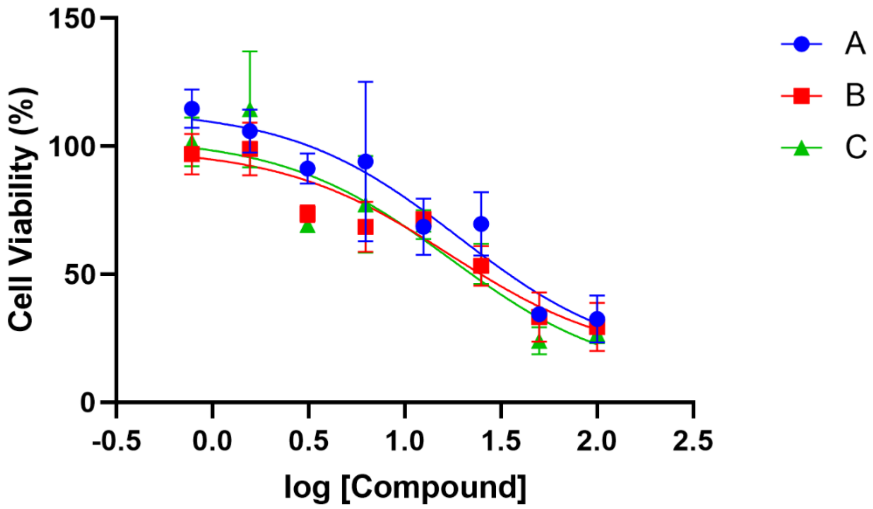

Figure S35. Dose-response IC_{50} curve of compound **PdC1** against the MCF-7 cell line. The x-axis represents the logarithm of the concentration ($\mu mol \cdot L^{-1}$). Curves A (blue circles), B (red squares), and C (green triangles) represent three independent experimental replicates performed in triplicate. Data are expressed as mean ± standard deviation.

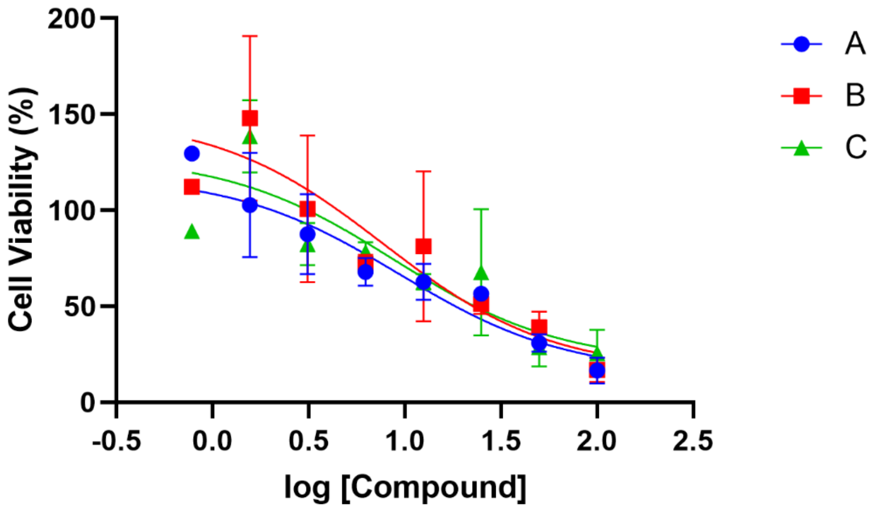

Figure S36. Dose-response IC_{50} curve of compound **CDDP** (Cisplatin) against the MCF-7 cell line. The x-axis represents the logarithm of the concentration ($\mu mol \cdot L^{-1}$). Curves A (blue circles), B (red squares), and C (green triangles) represent three independent experimental replicates performed in triplicate. Data are expressed as mean ± standard deviation.

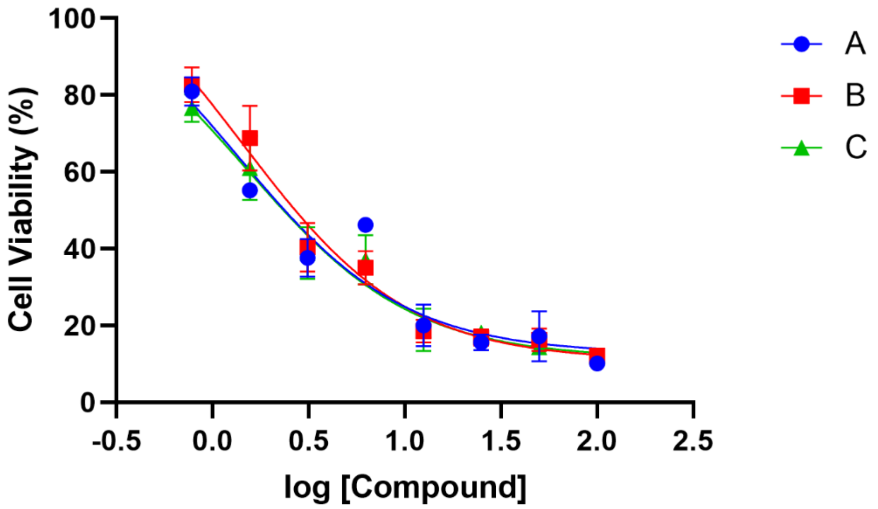

Figure S37. Dose-response IC_{50} curve of compound **PdB1** against the SK-BR-3 cell line. The x-axis represents the logarithm of the concentration ($\mu mol \cdot L^{-1}$). Curves A (blue circles), B (red squares), and C (green triangles) represent three independent experimental replicates performed in triplicate. Data are expressed as mean ± standard deviation.

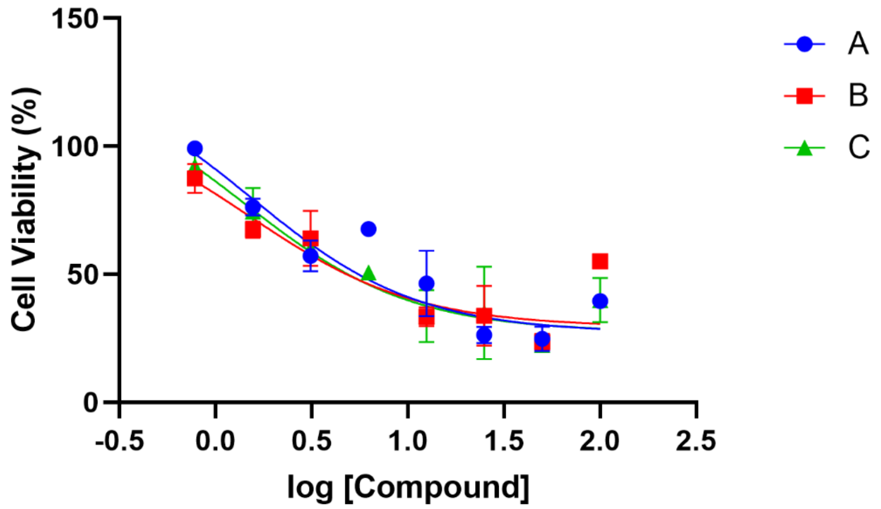

Figure S38. Dose-response IC_{50} curve of compound **PdB2** against the SK-BR-3 cell line. The x-axis represents the logarithm of the concentration ($\mu mol \cdot L^{-1}$). Curves A (blue circles), B (red squares), and C (green triangles) represent three independent experimental replicates performed in triplicate. Data are expressed as mean ± standard deviation.

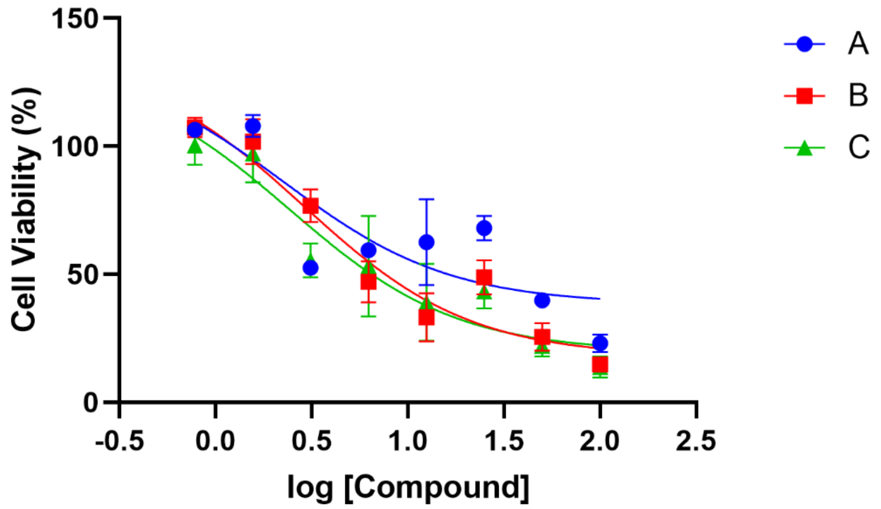

Figure S39. Dose-response IC_{50} curve of compound **PdC1** against the SK-BR-3 cell line. The x-axis represents the logarithm of the concentration ($\mu mol \cdot L^{-1}$). Curves A (blue circles), B (red squares), and C (green triangles) represent three independent experimental replicates performed in triplicate. Data are expressed as mean ± standard deviation.

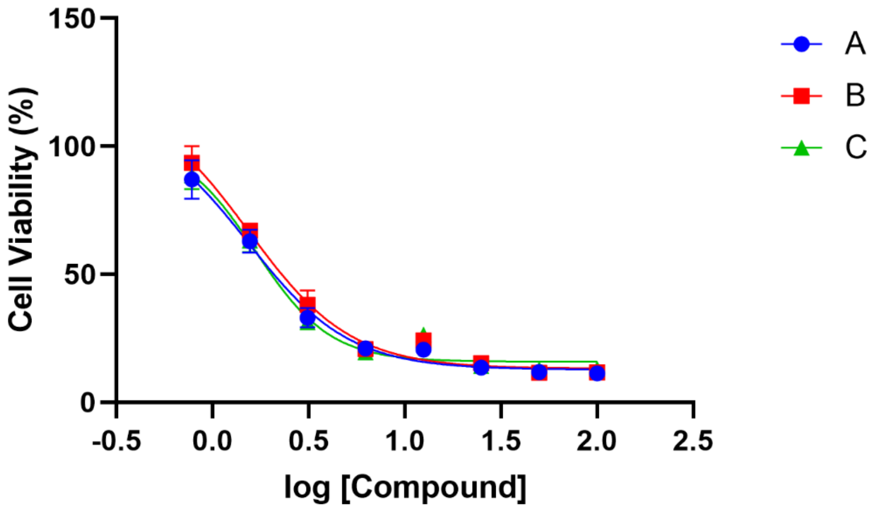

Figure S40. Dose-response IC_{50} curve of compound **PdB1** against the MCF10A cell line. The x-axis represents the logarithm of the concentration (µmol·L^{-1}). Curves A (blue circles), B (red squares), and C (green triangles) represent three independent experimental replicates performed in triplicate. Data are expressed as mean ± standard deviation.

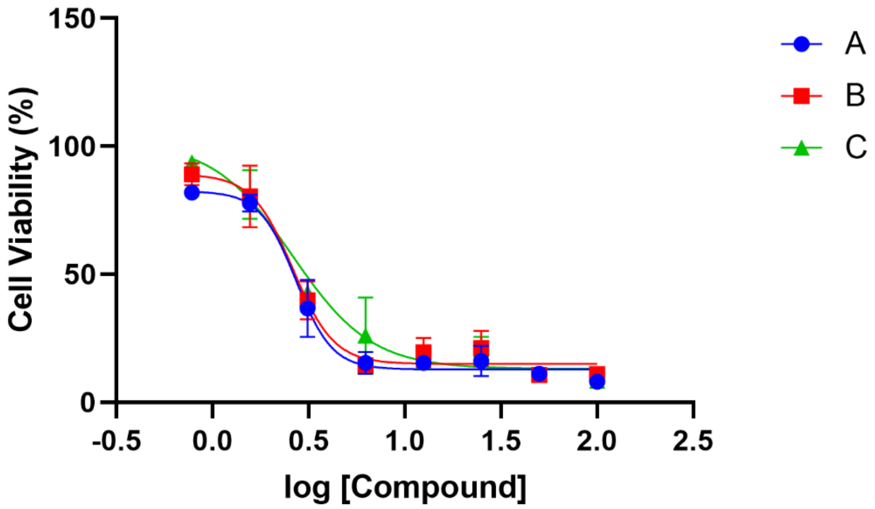

Figure S41. Dose-response $IC5_0$ curve of compound **PdC1** against the MCF10A cell line. The x-axis represents the logarithm of the concentration (µmol·L^{-1}). Curves A (blue circles), B (red squares), and C (green triangles) represent three independent experimental replicates performed in triplicate. Data are expressed as mean ± standard deviation.

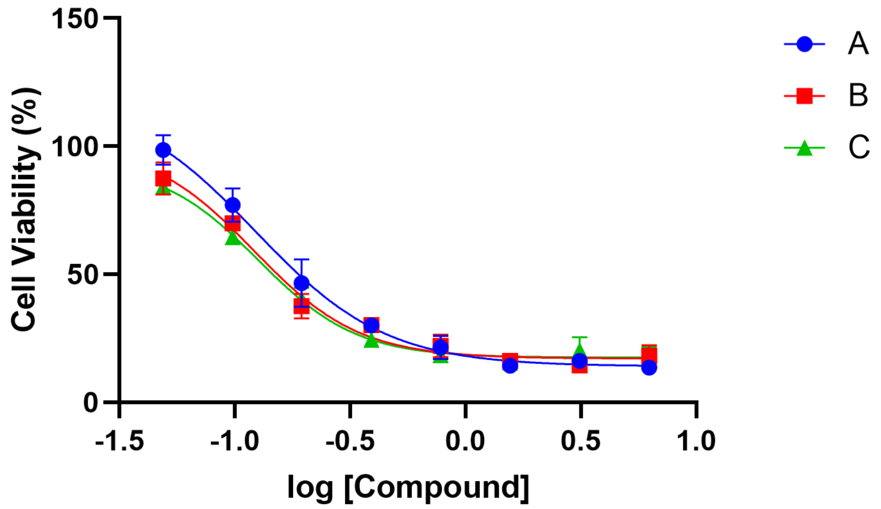

Figure S42. Dose-response IC_{50} curve of compound **PdB1** against the A2780 cell line. The x-axis represents the logarithm of the concentration (μmol·L^{-1}). Curves A (blue circles), B (red squares), and C (green triangles) represent three independent experimental replicates performed in triplicate. Data are expressed as mean ± standard deviation.

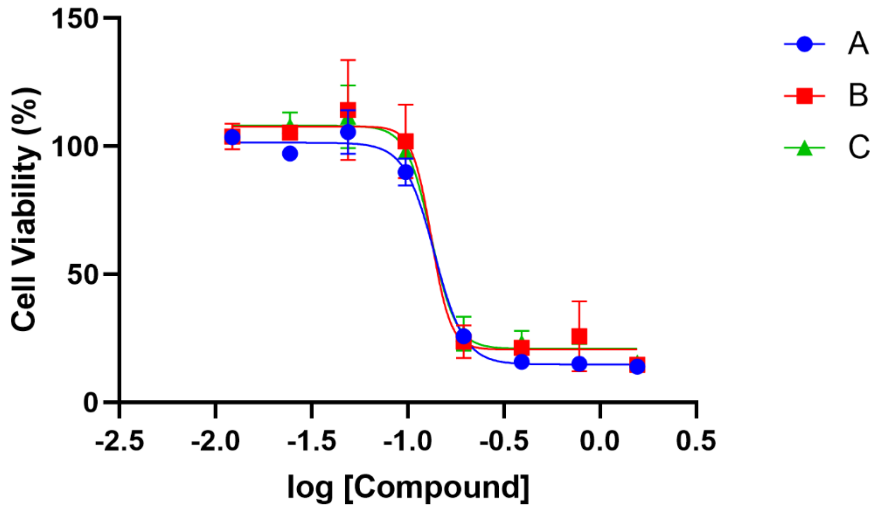

Figure S43. Dose-response IC_{50} curve of compound **PdB2** against the A2780 cell line. The x-axis represents the logarithm of the concentration (μmol·L^{-1}). Curves A (blue circles), B (red squares), and C (green triangles) represent three independent experimental replicates performed in triplicate. Data are expressed as mean ± standard deviation.

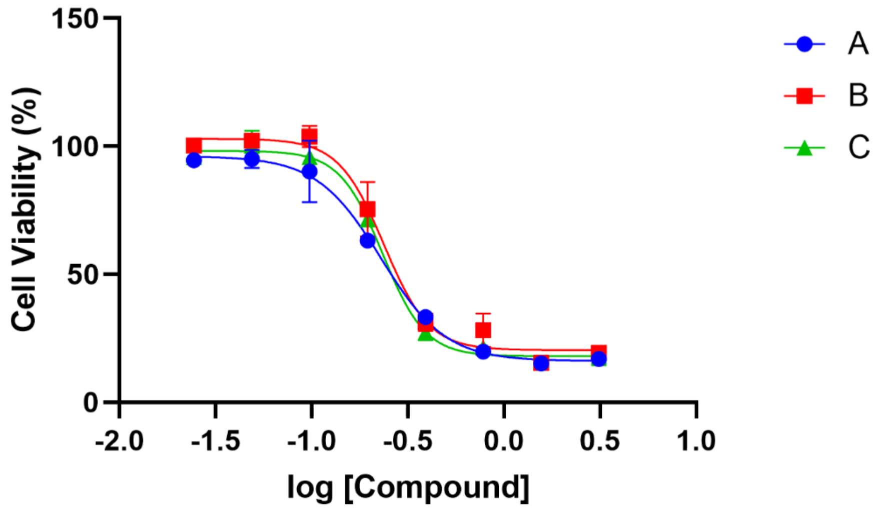

Figure S44. Dose-response IC_{50} curve of compound **PdB3** against the A2780 cell line. The x-axis represents the logarithm of the concentration (μmol·L^{-1}). Curves A (blue circles), B (red squares), and C (green triangles) represent three independent experimental replicates performed in triplicate. Data are expressed as mean ± standard deviation.

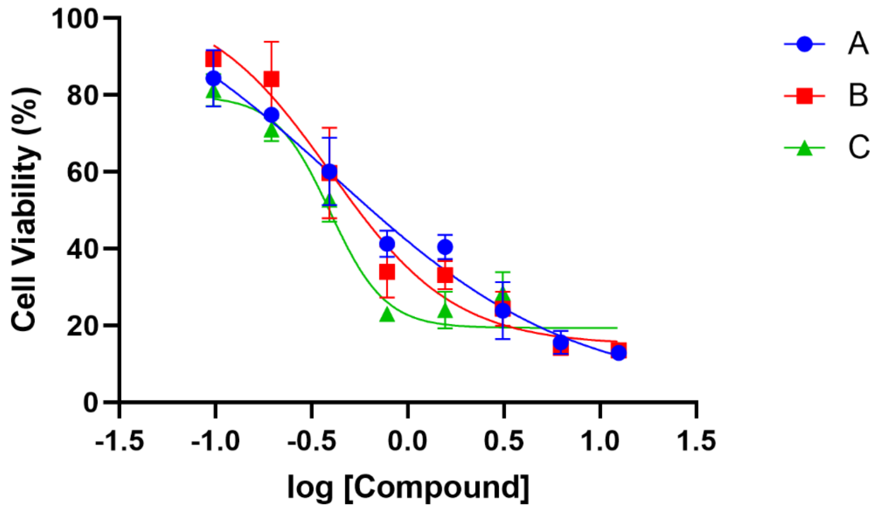

Figure S45. Dose-response IC_{50} curve of compound **PdC1** against the A2780 cell line. The x-axis represents the logarithm of the concentration (μmol·L^{-1}). Curves A (blue circles), B (red squares), and C (green triangles) represent three independent experimental replicates performed in triplicate. Data are expressed as mean ± standard deviation.

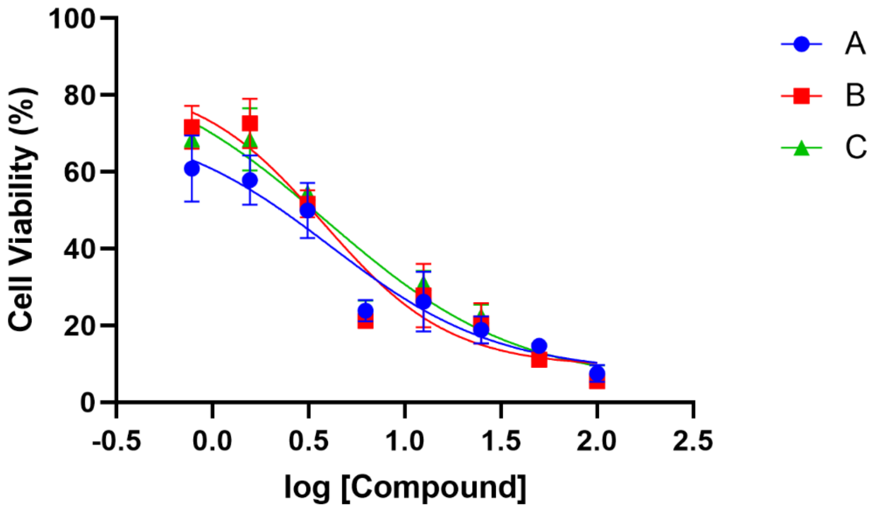

Figure S46. Dose-response IC_{50} curve of compound **PdC2** against the A2780 cell line. The x-axis represents the logarithm of the concentration (μmol·L^{-1}). Curves A (blue circles), B (red squares), and C (green triangles) represent three independent experimental replicates performed in triplicate. Data are expressed as mean ± standard deviation.

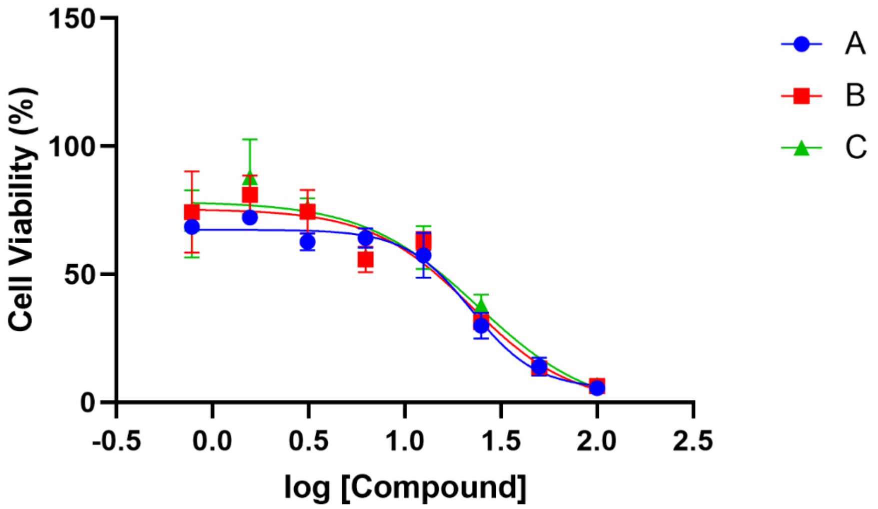

Figure S47. Dose-response IC_{50} curve of compound **PdC3** against the A2780 cell line. The x-axis represents the logarithm of the concentration (μmol·L^{-1}). Curves A (blue circles), B (red squares), and C (green triangles) represent three independent experimental replicates performed in triplicate. Data are expressed as mean ± standard deviation.

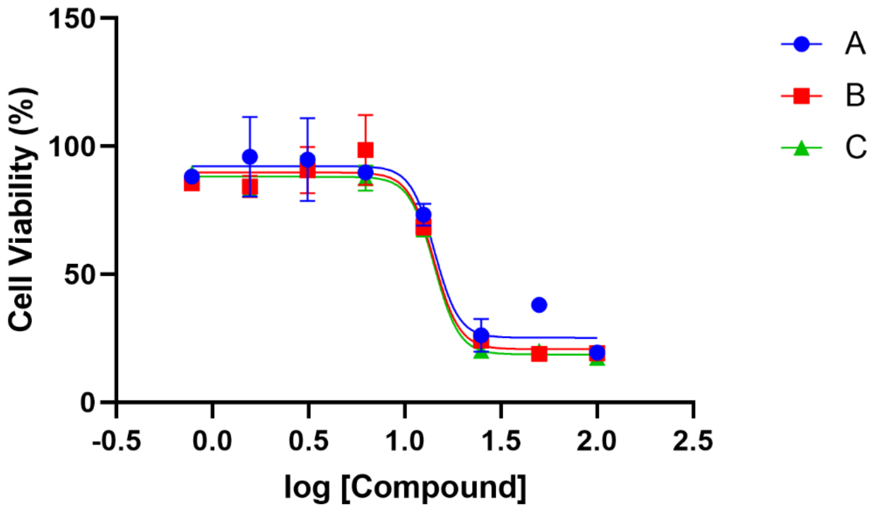

Figure S48. Dose-response IC_{50} curve of compound **CDDP** (Cisplatin) against the A2780 cell line. The x-axis represents the logarithm of the concentration (μmol·L^{-1}). Curves A (blue circles), B (red squares), and C (green triangles) represent three independent experimental replicates performed in triplicate. Data are expressed as mean ± standard deviation.

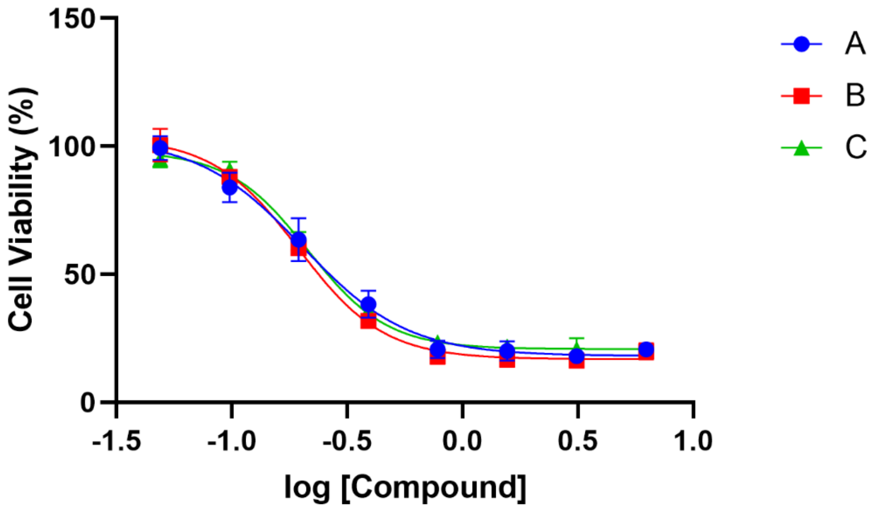

Figure S49. Dose-response IC_{50} curve of compound **PdB1** against the A2780cis cell line. The x-axis represents the logarithm of the concentration (μmol·L^{-1}). Curves A (blue circles), B (red squares), and C (green triangles) represent three independent experimental replicates performed in triplicate. Data are expressed as mean ± standard deviation.

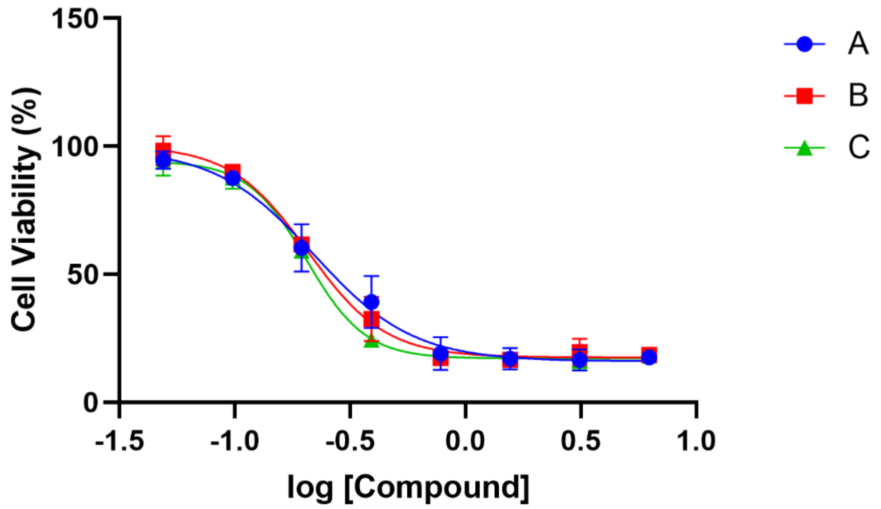

Figure S50. Dose-response IC_{50} curve of compound **PdB2** against the A2780cis cell line. The x-axis represents the logarithm of the concentration (μmol·L^{-1}). Curves A (blue circles), B (red squares), and C (green triangles) represent three independent experimental replicates performed in triplicate. Data are expressed as mean ± standard deviation.

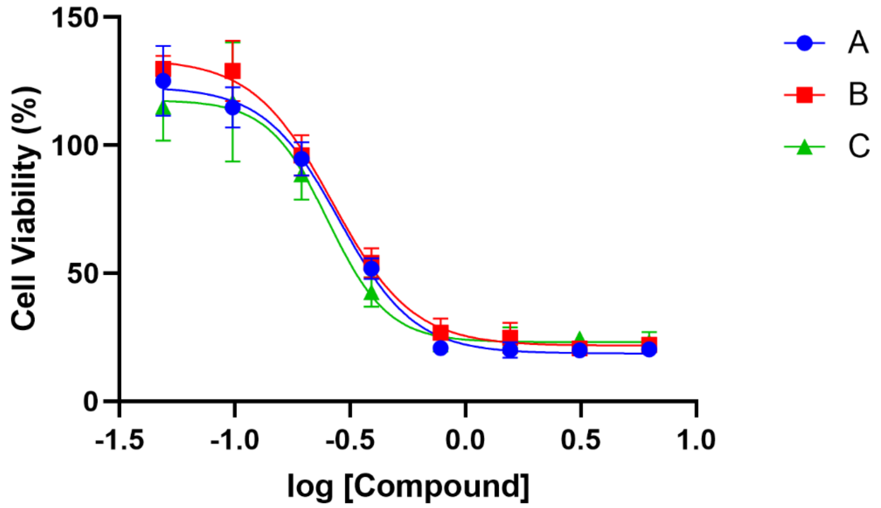

Figure S51. Dose-response IC_{50} curve of compound **PdB3** against the A2780cis cell line. The x-axis represents the logarithm of the concentration (μmol·L^{-1}). Curves A (blue circles), B (red squares), and C (green triangles) represent three independent experimental replicates performed in triplicate. Data are expressed as mean ± standard deviation.

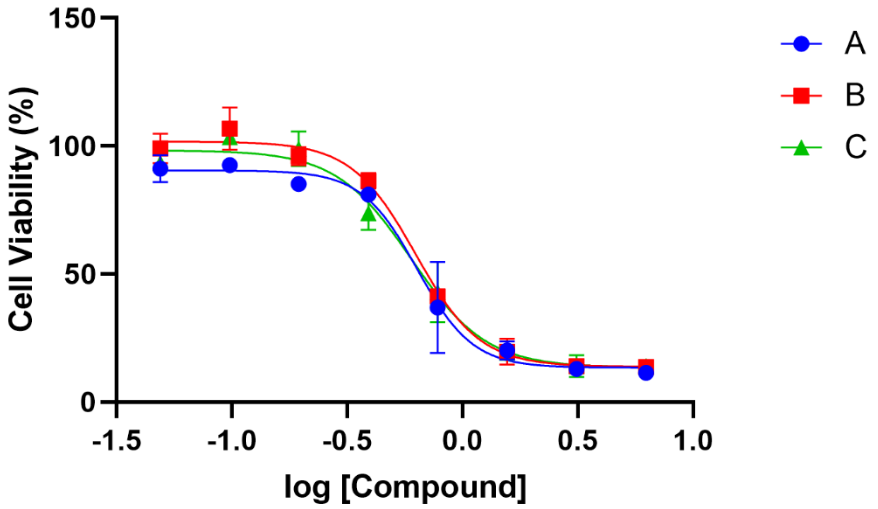

Figure S52. Dose-response IC_{50} curve of compound **PdC1** against the A2780cis cell line. The x-axis represents the logarithm of the concentration (μmol·L^{-1}). Curves A (blue circles), B (red squares), and C (green triangles) represent three independent experimental replicates performed in triplicate. Data are expressed as mean ± standard deviation.

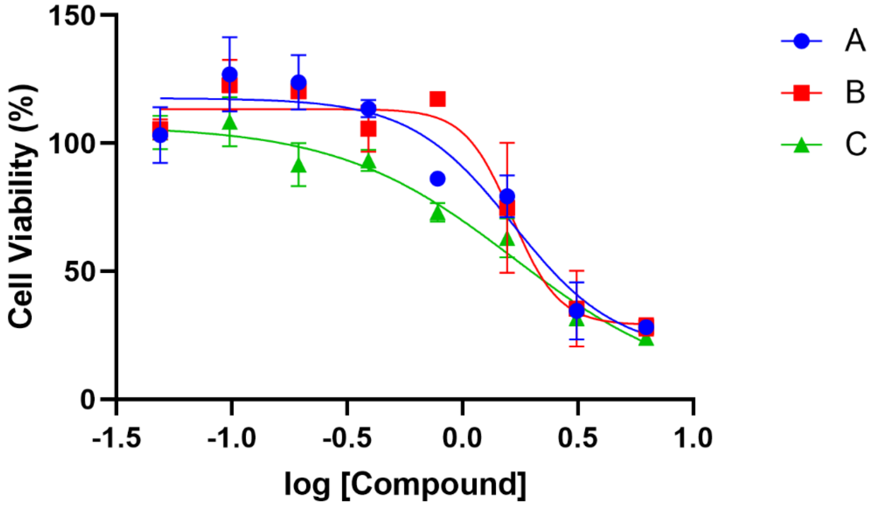

Figure S53. Dose-response IC_{50} curve of compound **PdC2** against the A2780cis cell line. The x-axis represents the logarithm of the concentration (μmol·L^{-1}). Curves A (blue circles), B (red squares), and C (green triangles) represent three independent experimental replicates performed in triplicate. Data are expressed as mean ± standard deviation.

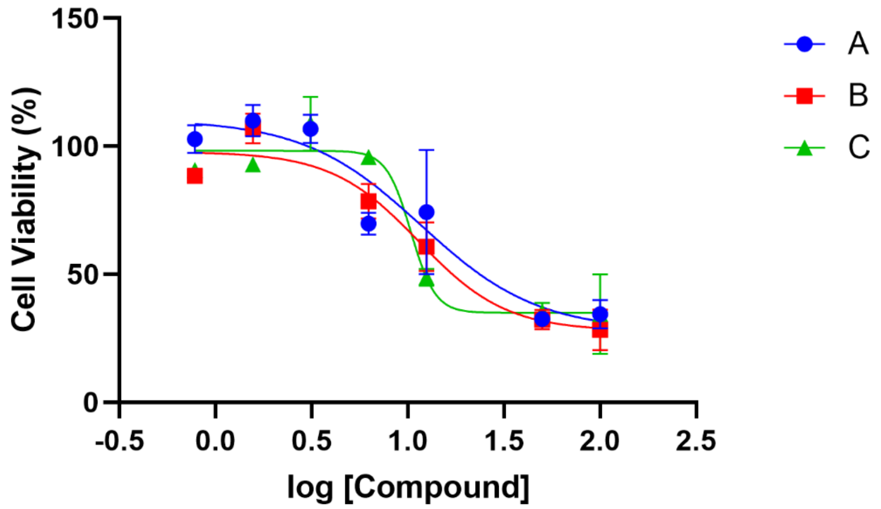

Figure S54. Dose-response IC_{50} curve of compound **PdC3** against the A2780cis cell line. The x-axis represents the logarithm of the concentration ($\mu mol \cdot L^{-1}$). Curves A (blue circles), B (red squares), and C (green triangles) represent three independent experimental replicates performed in triplicate. Data are expressed as mean ± standard deviation.

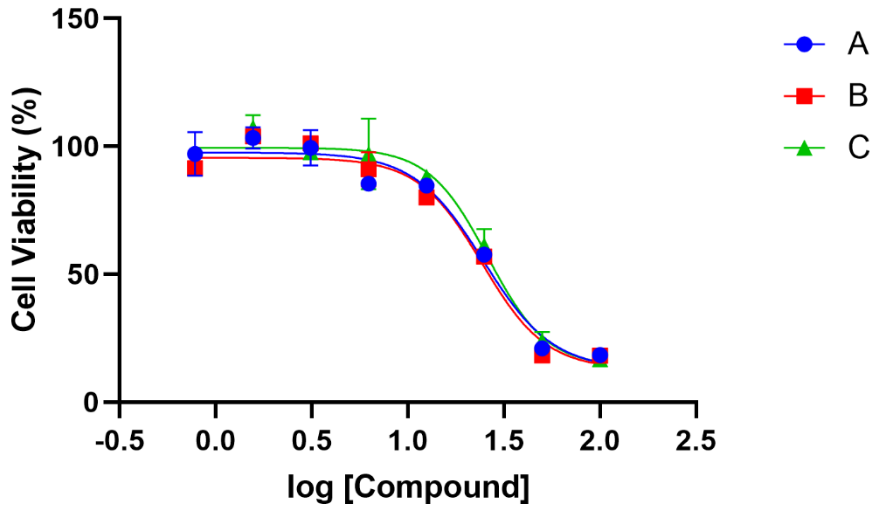

Figure S55. Dose-response IC_{50} curve of compound **CDDP** (Cisplatin) against the A2780cis cell line. The x-axis represents the logarithm of the concentration ($\mu mol \cdot L^{-1}$). Curves A (blue circles), B (red squares), and C (green triangles) represent three independent experimental replicates performed in triplicate. Data are expressed as mean ± standard deviation.

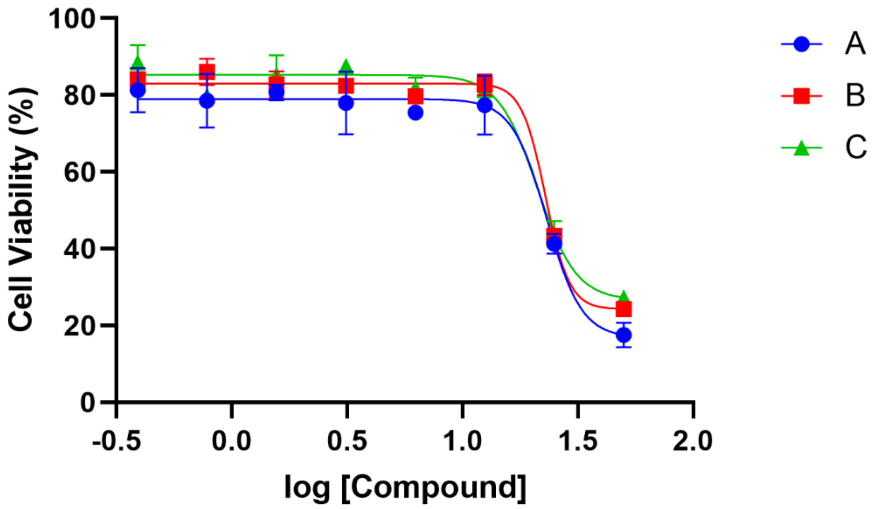

Figure S56. Dose-response IC_{50} curve of compound **PdB1** against the A549 cell line. The x-axis represents the logarithm of the concentration (μmol·L^{-1}). Curves A (blue circles), B (red squares), and C (green triangles) represent three independent experimental replicates performed in triplicate. Data are expressed as mean ± standard deviation.

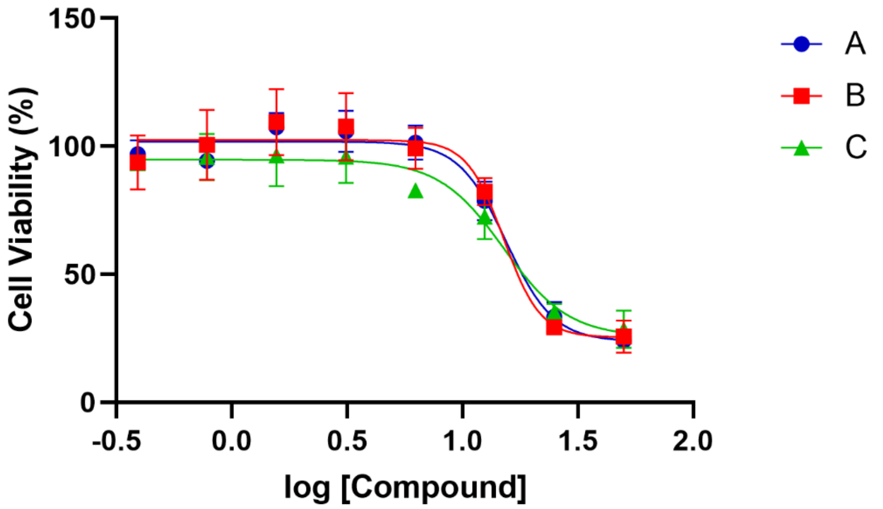

Figure S57. Dose-response IC_{50} curve of compound **PdB2** against the A549 cell line. The x-axis represents the logarithm of the concentration (μmol·L^{-1}). Curves A (blue circles), B (red squares), and C (green triangles) represent three independent experimental replicates performed in triplicate. Data are expressed as mean ± standard deviation.

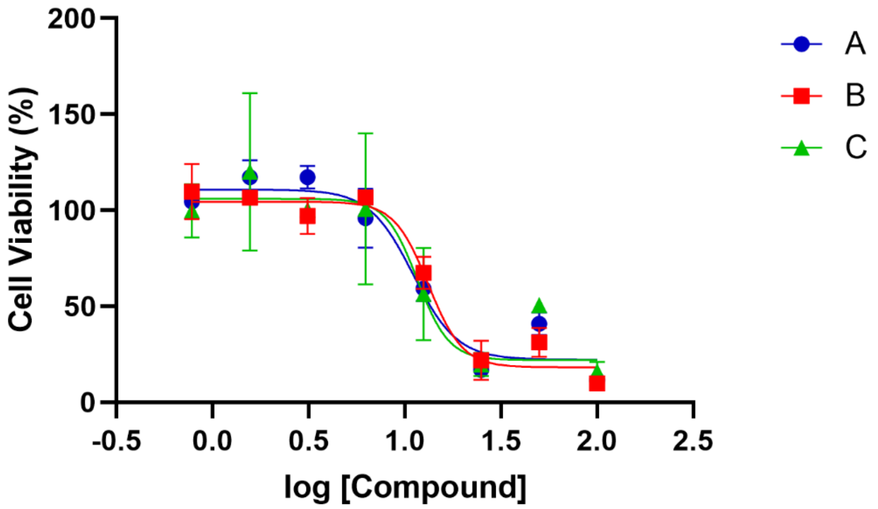

Figure S58. Dose-response IC_{50} curve of compound **PdB3** against the A549 cell line. The x-axis represents the logarithm of the concentration (μmol·L^{-1}). Curves A (blue circles), B (red squares), and C (green triangles) represent three independent experimental replicates performed in triplicate. Data are expressed as mean ± standard deviation.

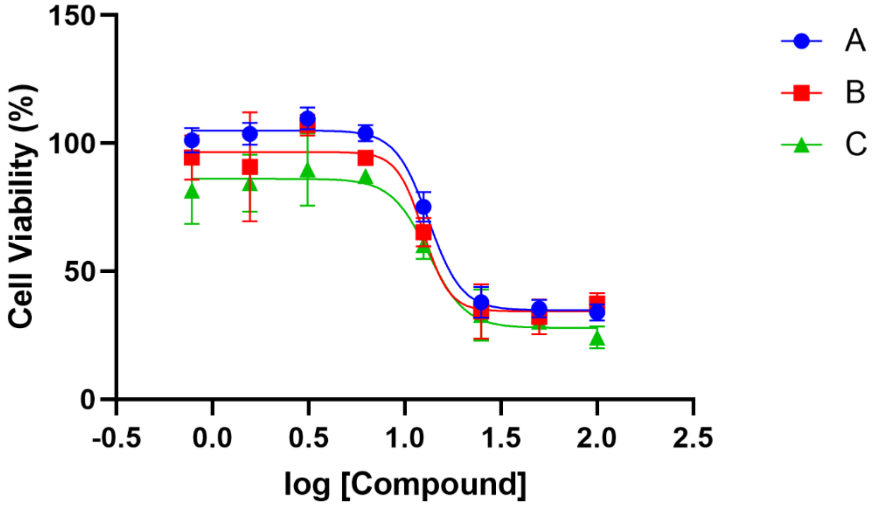

Figure S59. Dose-response IC_{50} curve of compound **CDDP** (Cisplatin) against the A549 cell line. The x-axis represents the logarithm of the concentration (μmol·L^{-1}). Curves A (blue circles), B (red squares), and C (green triangles) represent three independent experimental replicates performed in triplicate. Data are expressed as mean ± standard deviation.

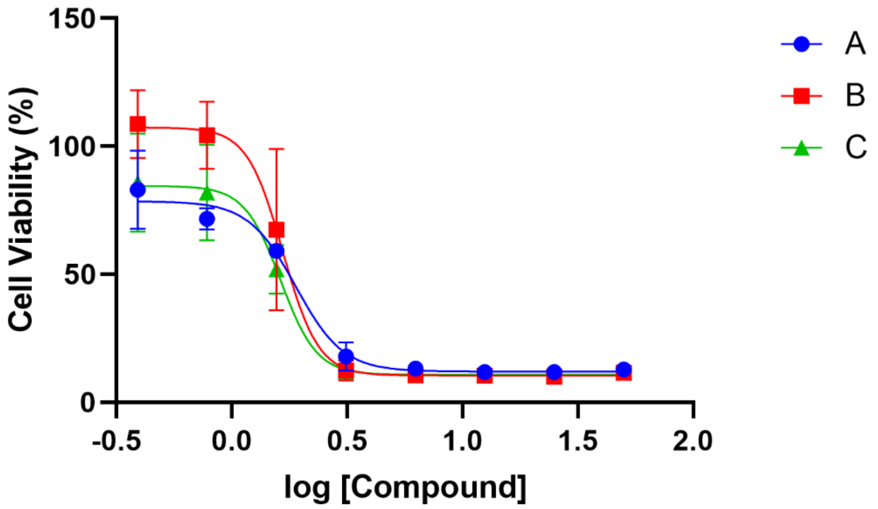

Figure S60. Dose-response IC_{50} curve of compound **PdB1** against the MRC5 cell line. The x-axis represents the logarithm of the concentration ($\mu mol \cdot L^{-1}$). Curves A (blue circles), B (red squares), and C (green triangles) represent three independent experimental replicates performed in triplicate. Data are expressed as mean ± standard deviation.

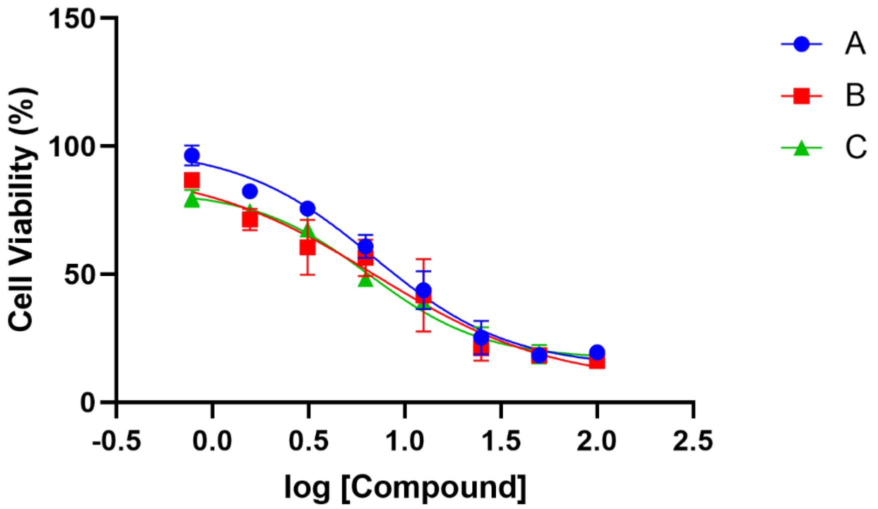

Figure S61. Dose-response IC_{50} curve of compound **PdB2** against the MRC5 cell line. The x-axis represents the logarithm of the concentration ($\mu mol \cdot L^{-1}$). Curves A (blue circles), B (red squares), and C (green triangles) represent three independent experimental replicates performed in triplicate. Data are expressed as mean ± standard deviation.

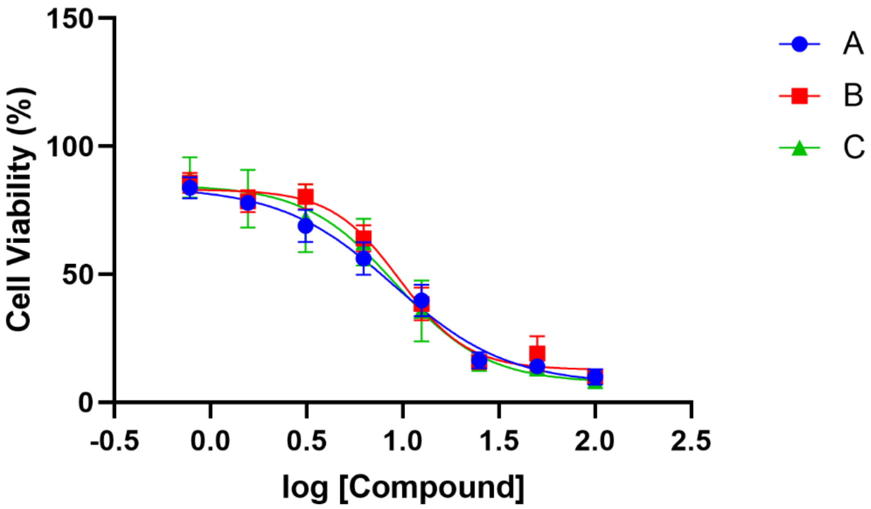

Figure S62. Dose-response IC_{50} curve of compound **PdB3** against the MRC5 cell line. The x-axis represents the logarithm of the concentration (μmol·L^{-1}). Curves A (blue circles), B (red squares), and C (green triangles) represent three independent experimental replicates performed in triplicate. Data are expressed as mean ± standard deviation.

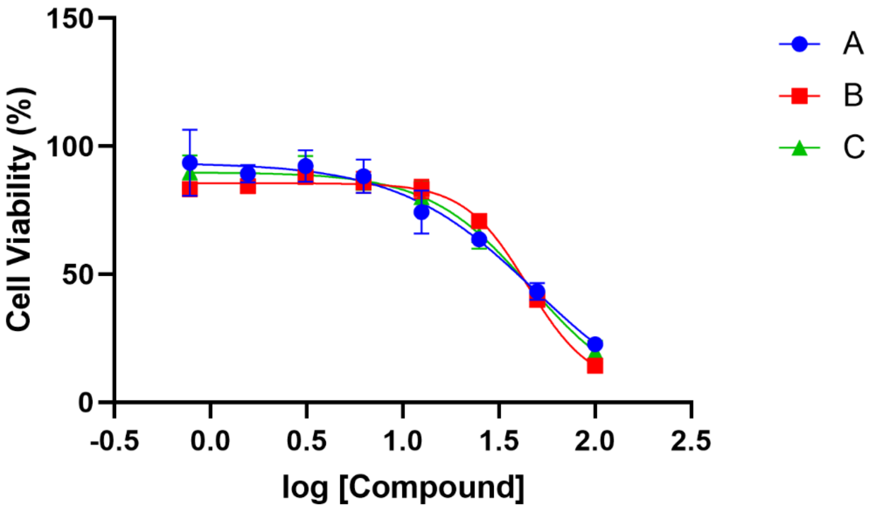

Figure S63. Dose-response IC_{50} curve of compound **PdC1** against the MRC5 cell line. The x-axis represents the logarithm of the concentration (μmol·L^{-1}). Curves A (blue circles), B (red squares), and C (green triangles) represent three independent experimental replicates performed in triplicate. Data are expressed as mean ± standard deviation.

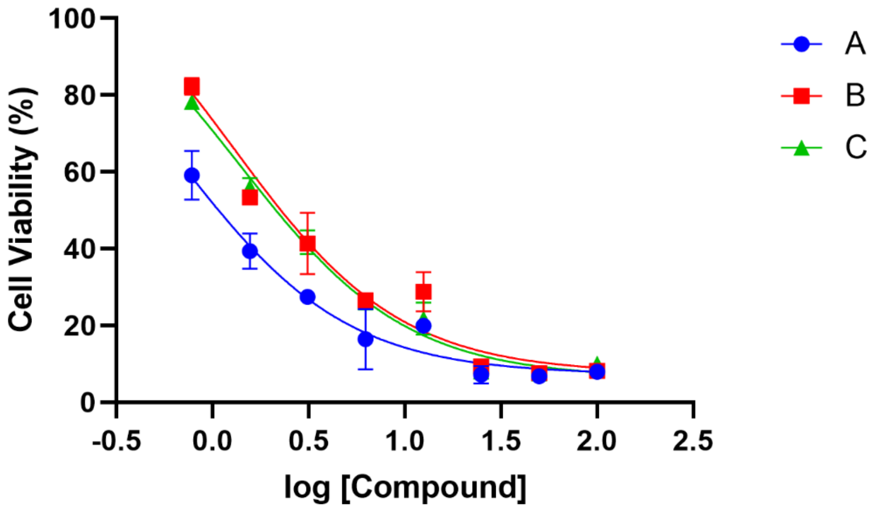

Figure S64. Dose-response IC_{50} curve of compound **PdB1** against the A375 cell line. The x-axis represents the logarithm of the concentration (μmol·L^{-1}). Curves A (blue circles), B (red squares), and C (green triangles) represent three independent experimental replicates performed in triplicate. Data are expressed as mean ± standard deviation.

IC_{50} Bar Charts and Statistical Analysis

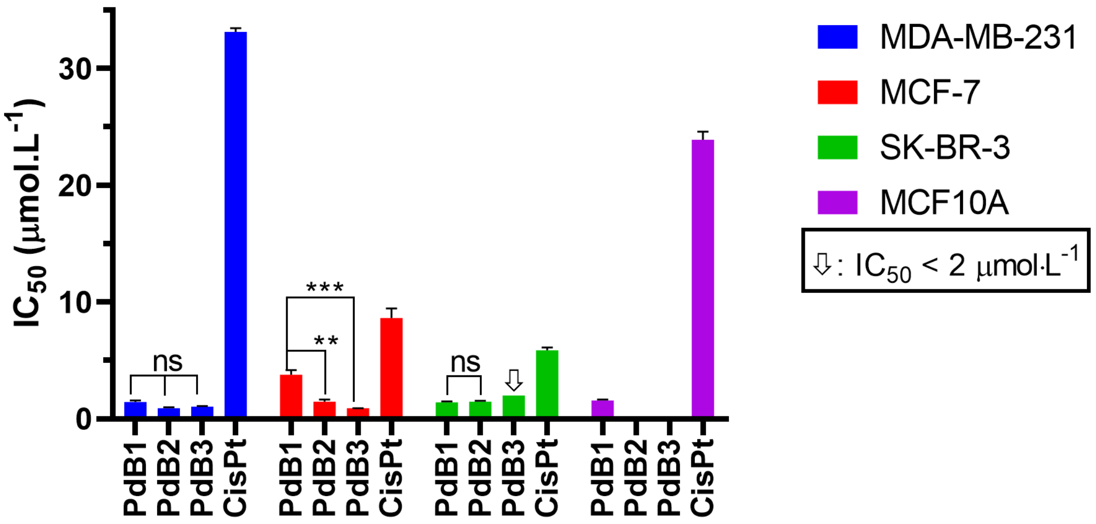

Figure S65. IC_{50} values (µmol·L^{-1}) of Pd(II) complexes (**PdB1**, **PdB2**, **PdB3**, **PdC1**, **PdC2**, **PdC3**) and cisplatin (CDDP) against breast cancer cell lines MDA-MB-231, MCF-7, SK-BR-3, and the non-tumorigenic epithelial cell line MCF10A. Data are presented as mean ± standard deviation. Statistical analysis was performed using one-way ANOVA, comparing the IC_{50} values of each Pd(II) complex to the respective IC_{50} value of cisplatin (CDDP) within each cell line. Significance levels are indicated as ***p < 0.001, **p < 0.01 and ns (not significant). Arrows indicate: ↑ IC_{50} > 50 µmol·L^{-1}; ↓ IC_{50} < 2 µmol·L^{-1}.

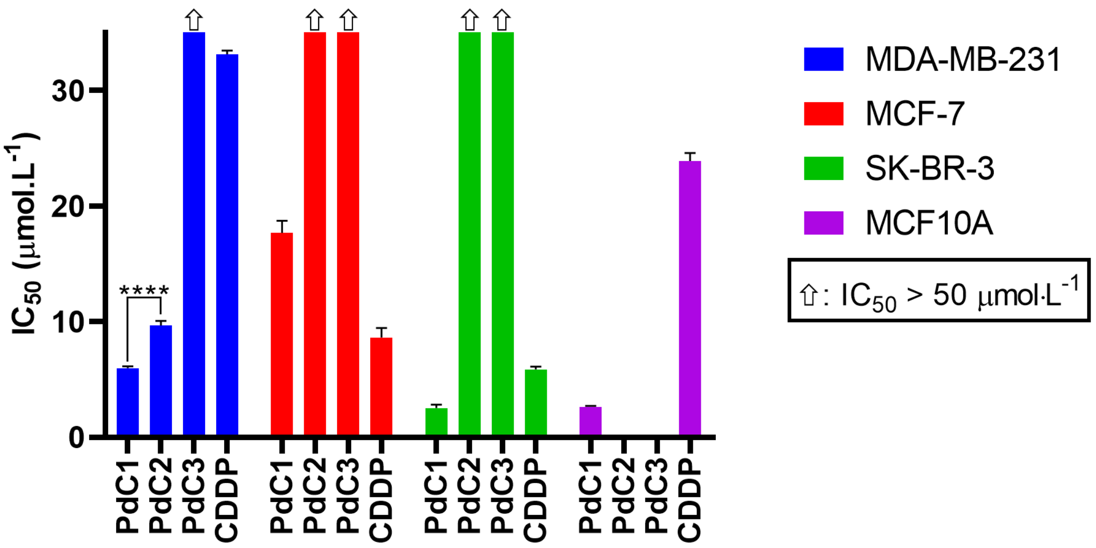

Figure S66. IC_{50} values (µmol·L^{-1}) of Pd(II) complexes **PdC1**, **PdC2**, and **PdC3** compared within each cell line. CDDP was included as a reference compound. Data are presented as mean ± standard deviation. Statistical analysis was performed using one-way ANOVA, comparing IC_{50} values among PdC1, PdC2, and PdC3 within each cell line. Significance levels are indicated as ****p < 0.0001 and ns (not significant). The upward arrow (↑) indicates IC_{50} > 50 µmol·L^{-1}.

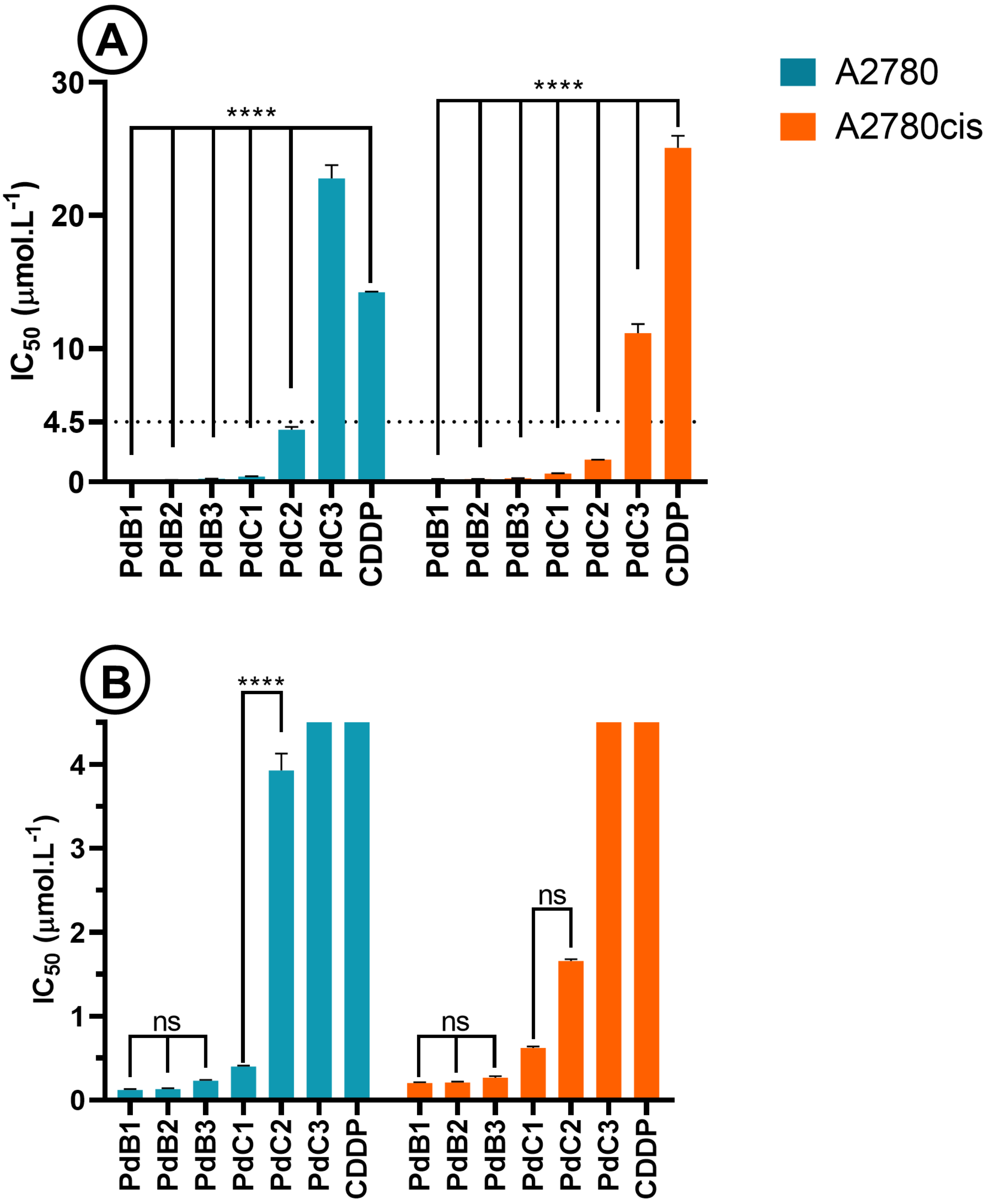

Figure S67. (**A**) IC_{50} values (µmol·L^{-1}) of Pd(II) complexes **PdB1**, **PdB2**, **PdB3**, **PdC1**, **PdC2**, and **PdC3** against A2780 (cisplatin-sensitive) and A2780cis (cisplatin-resistant) ovarian cancer cell lines. CDDP was included as a reference compound. The dashed line at 4.5 µmol·L^{-1} indicates the threshold adopted for better visualization in panel B. (**B**) Magnification of panel A highlighting compounds with IC_{50} values below 4.5 µmol·L^{-1}. Data are presented as mean ± standard deviation. Statistical analysis was performed using two-way ANOVA followed by Sidak's multiple comparisons test. Significance levels are indicated as ****$p < 0.0001$ and ns (not significant).

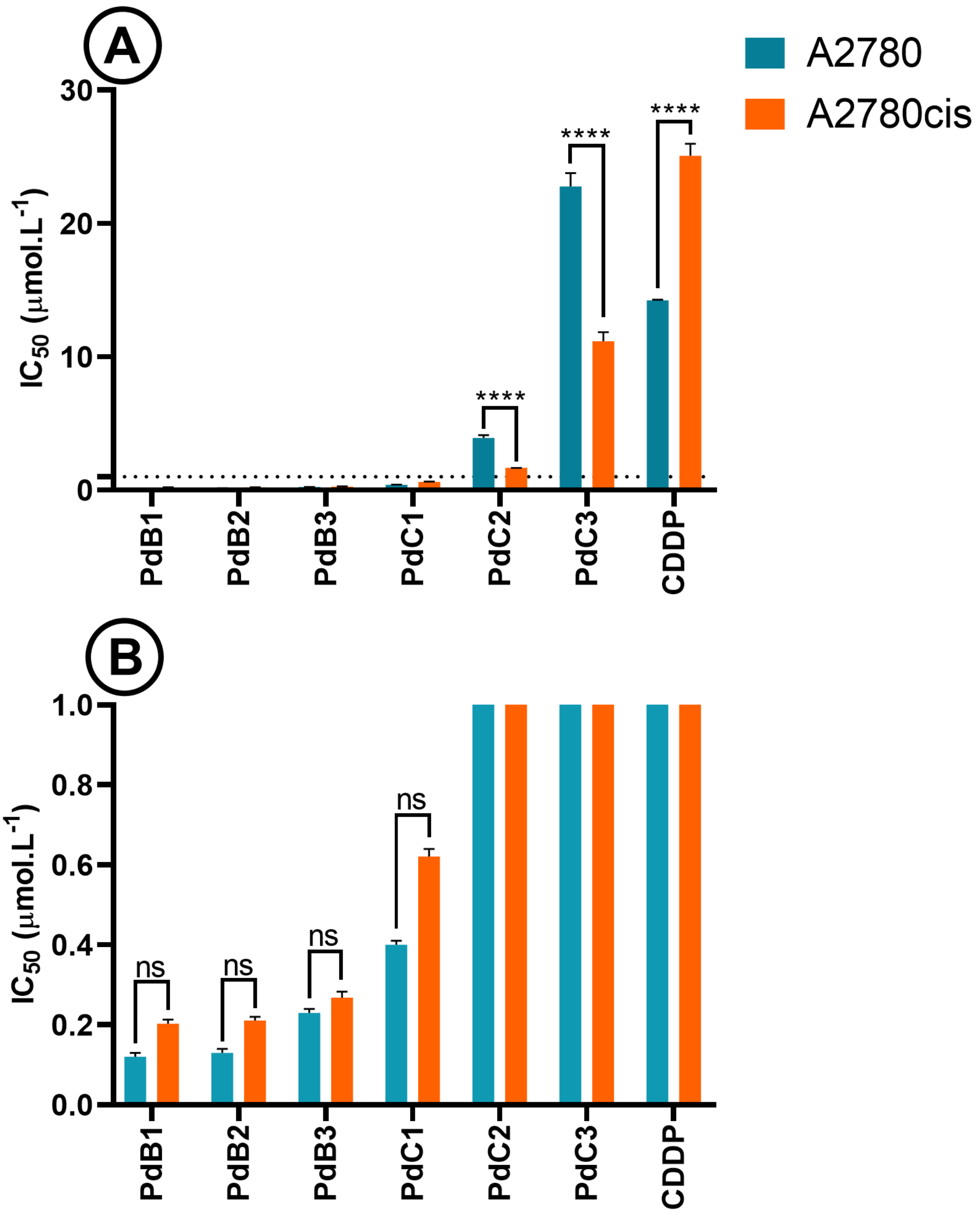

Figure S68. (**A**) IC_{50} values (μmol·L^{-1}) of Pd(II) complexes **PdB1**, **PdB2**, **PdB3**, **PdC1**, **PdC2**, and **PdC3** in ovarian cancer cell lines sensitive (A2780, cyan bars) and resistant (A2780cis, orange bars) to cisplatin. CDDP was included as a reference compound. The dashed line at 1 μmol·L^{-1} indicates the threshold adopted for better visualization in panel B. (**B**) Magnification of panel A highlighting compounds with IC_{50} values below 1 μmol·L^{-1}. Data are presented as mean ± standard deviation. Statistical analysis was performed using two-way ANOVA followed by Sidak's multiple comparisons test. Significance levels are indicated as ****$p < 0.0001$ and ns (not significant).

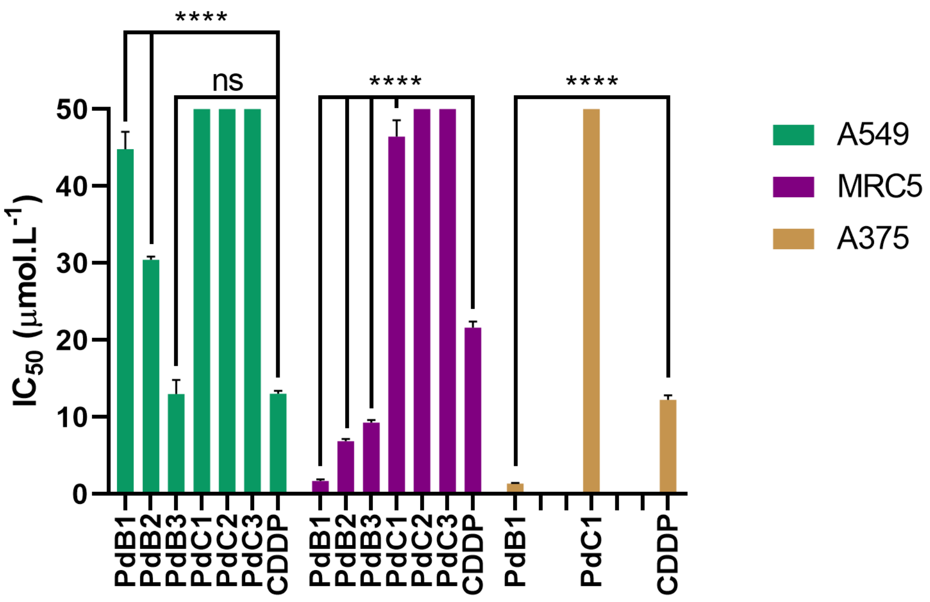

Figure S69. IC_{50} values (μmol·L^{-1}) of Pd(II) complexes **PdB1**, **PdB2**, **PdB3**, **PdC1**, **PdC2**, **PdC3**, and cisplatin (CDDP) against A549 (lung cancer), MRC-5 (normal lung fibroblast), and A375 (melanoma) cell lines. CDDP was included as a reference compound. The comparisons were performed between each Pd(II) complex and cisplatin within the same cell line. Data are presented as mean ± standard deviation. Statistical analysis was performed using two-way ANOVA followed by Tukey's multiple comparisons test. Significance levels are indicated as ****$p < 0.0001$ and ns (not significant).

Morphologic assay

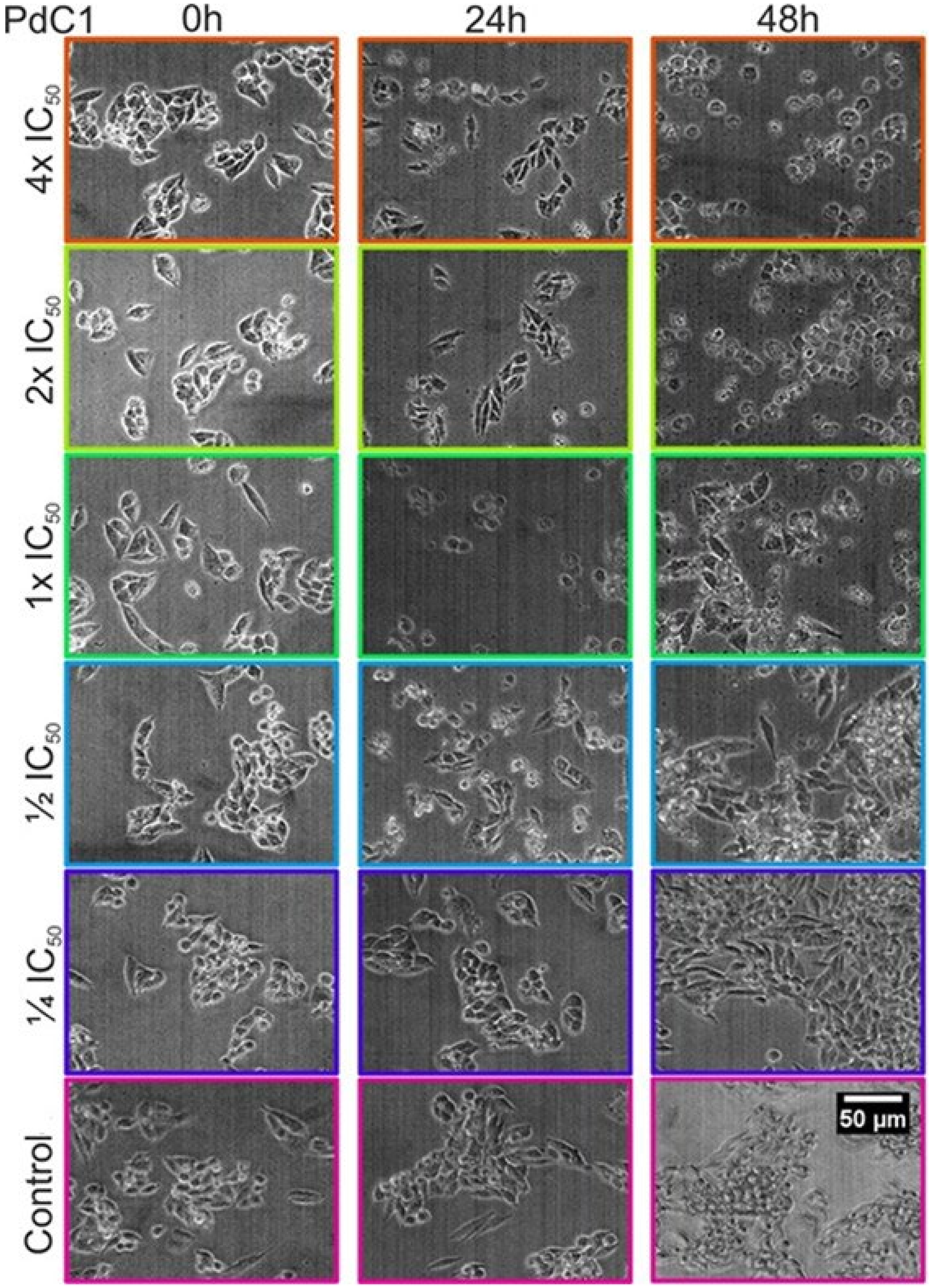

Figure S70. Microscopy images of the morphological evaluation assay for the **PdC1** complex at different concentrations, in the A2780cis cell line and different times.

Cell migration assay

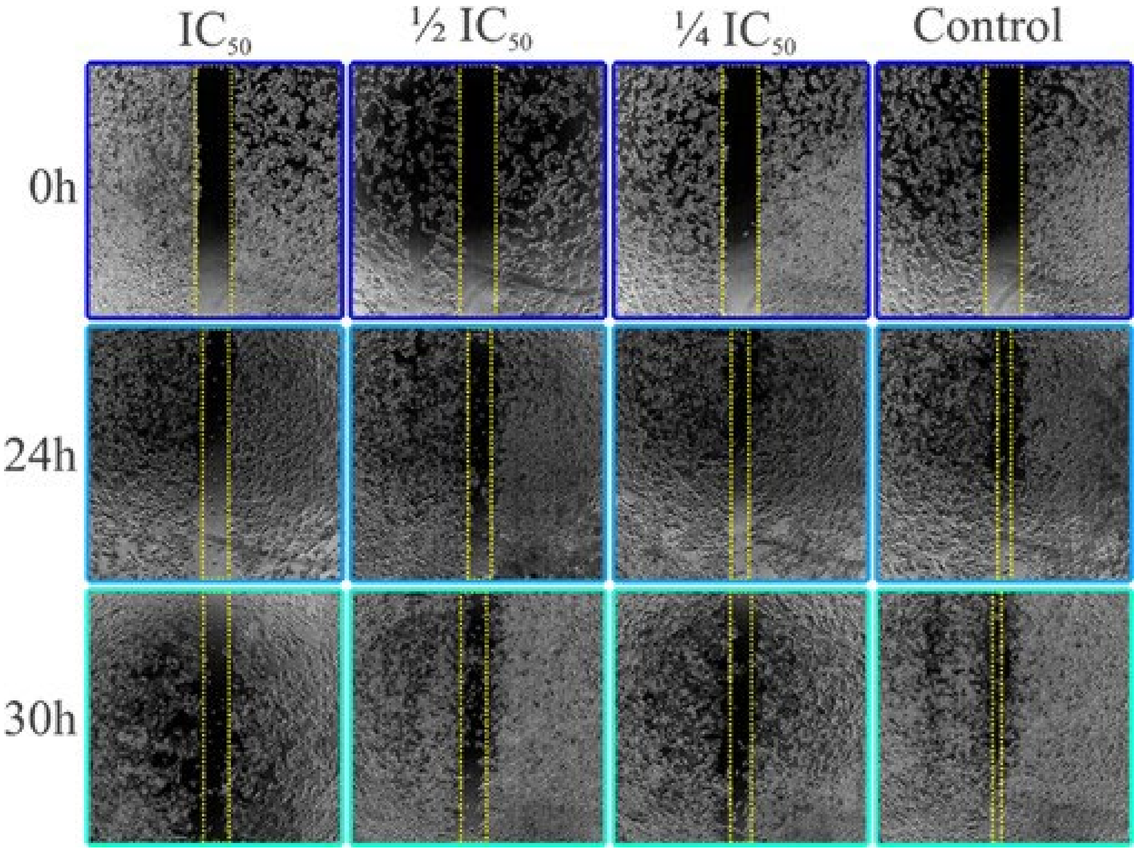

Figure S71. Microscopy images of the cell migration assay at times 0 h and 30 h after the addition of the compound **PdC1** at different concentrations, in the A2780cis cell line. Images obtained using a Nikon Eclipse TS 100 microscope with 4× zoom.
